# A faecal microbiota signature with high specificity for pancreatic cancer

**DOI:** 10.1136/gutjnl-2021-324755

**Published:** 2022-03-08

**Authors:** Ece Kartal, Thomas S B Schmidt, Esther Molina-Montes, Sandra Rodríguez-Perales, Jakob Wirbel, Oleksandr M Maistrenko, Wasiu A Akanni, Bilal Alashkar Alhamwe, Renato J Alves, Alfredo Carrato, Hans-Peter Erasmus, Lidia Estudillo, Fabian Finkelmeier, Anthony Fullam, Anna M Glazek, Paulina Gómez-Rubio, Rajna Hercog, Ferris Jung, Stefanie Kandels, Stephan Kersting, Melanie Langheinrich, Mirari Márquez, Xavier Molero, Askarbek Orakov, Thea Van Rossum, Raul Torres-Ruiz, Anja Telzerow, Konrad Zych, MAGIC Study investigators, PanGenEU Study investigators, Vladimir Benes, Georg Zeller, Jonel Trebicka, Francisco X Real, Nuria Malats, Peer Bork

**Affiliations:** 1 Structural and Computational Biology Unit, European Molecular Biology Laboratory, Heidelberg, Germany; 2 Collaboration for joint PhD degree, European Molecular Biology Laboratory and Heidelberg University, Heidelberg, Germany; 3 Genetic and Molecular Epidemiology Group, Spanish National Cancer Research Centre (CNIO), Madrid, Spain; 4 Centro de Investigación Biomédica en Red de Oncología (CIBERONC), Madrid, Spain; 5 Molecular Cytogenetics Unit, Spanish National Cancer Research Centre (CNIO), Madrid, Spain; 6 Member of the German Center for Lung Research (DZL) and the Universities of Giessen and Marburg Lung School (UGMLC), Philipps University Marburg Faculty of Medicine, Marburg, Germany; 7 Medical Oncology Department of Oncology, Hospital Ramón y Cajal, Madrid, Spain; 8 University of Alcala de Henares, Alcala de Henares, Spain; 9 Translational Hepatology Department of Internal Medicine I, Goethe-Universitat Frankfurt am Main, Frankfurt am Main, Germany; 10 Frankfurt Cancer Institute, Goethe University Frankfurt, Frankfurt am Main, Hessen, Germany; 11 Genomic Core Facility, European Molecular Biology Laboratory, Heidelberg, Germany; 12 Department of Surgery, Erlangen University Hospital, Erlangen, Germany; 13 Department of Surgery, University of Greifswald, Greifswald, Germany; 14 Hospital Universitari Vall d’Hebron, Institut de Recerca (VHIR), Barcelona, Spain; 15 Universitat Autònoma de Barcelona, Barcelona, Spain; 16 Centro de Investigación Biomédica en Red de Enfermedades Hepáticas y Digestivas (CIBEREHD), Madrid, Spain; 17 EF Clif, European Foundation for the Study of Chronic Liver Failure, Barcelona, Spain; 18 Epithelial Carcinogenesis Group, Spanish National Cancer Research Centre (CNIO), Madrid, Spain; 19 Departament de Ciències Experimentals i de la Salut, Universitat Pompeu Fabra, Barcelona, Spain; 20 Department of Bioinformatics, Biocenter, University of Würzburg, Würzburg, Germany; 21 Yonsei Frontier Lab (YFL), Yonsei University, Seoul, South Korea; 22 Max Delbrück Centre for Molecular Medicine, Berlin, Germany

**Keywords:** pancreatic cancer, intestinal microbiology, cancer prevention, pancreatic tumours, screening

## Abstract

**Background:**

Recent evidence suggests a role for the microbiome in pancreatic ductal adenocarcinoma (PDAC) aetiology and progression.

**Objective:**

To explore the faecal and salivary microbiota as potential diagnostic biomarkers.

**Methods:**

We applied shotgun metagenomic and 16S rRNA amplicon sequencing to samples from a Spanish case–control study (n=136), including 57 cases, 50 controls, and 29 patients with chronic pancreatitis in the discovery phase, and from a German case–control study (n=76), in the validation phase.

**Results:**

Faecal metagenomic classifiers performed much better than saliva-based classifiers and identified patients with PDAC with an accuracy of up to 0.84 area under the receiver operating characteristic curve (AUROC) based on a set of 27 microbial species, with consistent accuracy across early and late disease stages. Performance further improved to up to 0.94 AUROC when we combined our microbiome-based predictions with serum levels of carbohydrate antigen (CA) 19–9, the only current non-invasive, Food and Drug Administration approved, low specificity PDAC diagnostic biomarker. Furthermore, a microbiota-based classification model confined to PDAC-enriched species was highly disease-specific when validated against 25 publicly available metagenomic study populations for various health conditions (n=5792). Both microbiome-based models had a high prediction accuracy on a German validation population (n=76). Several faecal PDAC marker species were detectable in pancreatic tumour and non-tumour tissue using 16S rRNA sequencing and fluorescence in situ hybridisation.

**Conclusion:**

Taken together, our results indicate that non-invasive, robust and specific faecal microbiota-based screening for the early detection of PDAC is feasible.

**Significance of this study**

**What is already known about this subject?**

- Pancreatic ductal adenocarcinoma (PDAC) is on the rise worldwide, posing a high disease burden and mortality rate, yet accurate, non-invasive diagnostic options remain unavailable.
- Alterations in the oral, faecal and pancreatic microbiome composition have been associated with an increased risk of PDAC.

**What are the new findings?**

- Stool microbiota-based classifiers are described that predict PDAC with high accuracy and specificity, independent of disease stage, with potential as agents for non-invasive diagnostics.
- A faecal metagenomic classifier identified PDAC with an accuracy of 0.84 area under the receiver operating characteristic curve (AUROC) in a Spanish cohort, based on 27 species. The accuracy improved to up to 0.94 AUROC when combined with the less specific carbohydrate antigen (CA) 19–9 serum marker.
- The classifier was validated in an independent German PDAC cohort (0.83 AUROC), and PDAC disease specificity was confirmed against 25 publicly available metagenomic study populations with various health conditions (n=5792).
- The presence of marker taxa enriched in faecal samples (*Veillonella*, *Streptococcus*, *Akkermansia*) and also taxa with differential abundance in healthy and tumour pancreatic tissues (*Bacteroides*, *Lactobacillus*, *Bifidobacterium*) was validated by fluorescence *in situ* hybridisation.

**Significance of this study**

**How might it impact on clinical practice in the foreseeable future?**

- Faecal microbiome-based detection of PDAC may provide a non-invasive, cost-effective and robust approach to early PDAC diagnosis.
- The presented PDAC-specific microbiome signatures, including links between microbial populations across tissues, provide novel microbiome-related hypotheses regarding disease aetiology, prevention and possible therapeutic intervention.

## Introduction

Pancreatic ductal adenocarcinoma (PDAC) is the most common form of pancreatic cancer and a major cause of cancer-related deaths despite relatively low incidence rates.^1 2^ The high lethality of PDAC is a consequence of both late diagnosis and limited therapeutic options^3^: symptoms are unspecific and often emerge only during late disease stages, at which point tumours can be either locally non-resectable or present as metastatic disease. At present, PDAC is diagnosed using imaging tests.^4^ Sensitive and affordable tests for an early detection of PDAC could therefore improve outcome. PDAC markers have been explored in pancreatic tissue,^5^ urine^6 7^ and serum.^8 9^ Yet to date, the sole Food and Drug Administration (FDA)-approved PDAC biomarker remains serum carbohydrate antigen (CA) 19-9. CA19-9 has limited disease specificity as levels can be elevated in several other concomitant conditions (eg, biliary obstruction) and is therefore mostly used as a marker for PDAC surveillance, rather than screening or diagnosis.^10–14^

PDAC has a complex aetiology, with established risk factors that include age, chronic pancreatitis, diabetes mellitus, obesity, asthma, blood group and lifestyle (eg, smoking and heavy alcohol consumption).^15 16^ The role of these risk factors in PDAC aetiology may also be complemented—or sometimes indeed mediated—by alterations in the microbiome. For example, poor oral hygiene and periodontitis have been associated with an increased PDAC risk,^17^ an observation that also extends to periodontitis- and caries-associated microbial species.^18 19^ Shifts in these species are sometimes part of wider compositional changes in the oral microbiome^20 21^ or have been explored as PDAC risk factors in their own right.^22^ Similarly, microbial composition in the gut^23–25^ and duodenum,^26 27^ quantified via 16S rRNA amplicon sequencing, have previously been linked to PDAC risk.

The human pancreas harbours a microbiome that shares species with the mouth and the gut,^25 28–32^ although its exact composition has remained elusive owing to the challenges associated with contamination control in low bacterial biomass samples.^33^ In murine models, microbes originating from the intestine can contribute to carcinogenesis in the pancreatic duct,^25 30^ suggesting a role for the microbiome in PDAC aetiology and progression that was recently extended to fungi.^34^ Moreover, the pancreatic tumour microbiome may also be associated with disease progression and long-term survival in patients with PDAC.^31^

However, the translation of these advances into PDAC-specific microbiome signatures for clinical applications has so far remained largely unexplored. Here, we present the identification of robust, specific microbial PDAC signatures based on a metagenomic survey of a Spanish (ES) study population of 57 newly diagnosed and treatment-naïve patients with PDAC, 29 patients with chronic pancreatitis (CP), and 50 matched controls. We sampled saliva, faeces, pancreatic normal and tumour tissue and assessed microbial composition using whole-genome shotgun metagenomics, 16S rRNA amplicon sequencing, and fluorescence *in situ* hybridisation (FISH) assays. The best discrimination between patients with PDAC and non-PDAC subjects was achieved by statistical models based on a set of 27 faecal microbial species that could be quantified in a targeted manner in a diagnostic setting. The prediction accuracy of microbiome-based models was confirmed in an independent German (DE) PDAC validation population including 44 patients with PDAC and 32 controls and was further improved when combined with serum levels of CA19-9. We further validated the disease specificity of these models against existing data from 25 studies (n=5792) of nine diseases.^35–59^ Several of the PDAC-enriched species were also detected in cancer tissue, with possible links to oral and intestinal populations, supporting their potential role in PDAC pathogenesis, as previously reported.^25 30 31 34^

## Methods

### Subject recruitment and sample collection

A case–control design was applied. Subjects were prospectively recruited between 2016 and 2019 from the Hospital Ramón y Cajal in Madrid and Hospital Vall d’Hebron in Barcelona, Spain, using the same protocols for biological sample collection, processing and storage. Subjects with newly diagnosed PDAC (n=57), aged >18 years, were identified prior to any cancer treatment. Subjects in whom PDAC was suspected were recruited, and sampling was done before any treatment. Patients with chronic pancreatitis (CP, n=29) were recruited from the same hospitals. Controls matched for age, gender and hospital were selected from inpatients with a primary diagnosis for hospital admission not related to PDAC risk factors. Participants incapable of participating in the study owing to impairment of physical ability were excluded. Institutional review board ethical approval (CEI PI 26 2015-v7) and written informed consent were obtained from participating centres and study participants, respectively. Epidemiological and lifestyle data were collected by trained monitors during face-to-face interviews through a structured questionnaire. Clinical data, including stage of the diseases and follow-up data, were retrieved from hospital charts by the same monitors, likewise using structured questionnaires. Recorded jaundice status was additionally confirmed and extended by direct bilirubin measurements from blood samples in CNIO, Madrid. All data were entered, edited and managed using REDCap. Missing lifestyle and medication values in the metadata (missing overall in 3.1%) were imputed using a random forest-based algorithm for missing data imputation called missForest (n=100 trees).^60^ The imputation accuracy was high according to the imputation error estimate (mean out-of-bag error=0.12). Serum CA19-9 levels were analysed by electrochemiluminescence immunoassay (ECLIA, Roche Diagnostics, Germany) following the manufacturer's instructions in the Institute of Laboratory Medicine and Pathobiochemistry, Marburg, Germany. Each sample was assayed in duplicate, with positive controls assayed in each plate (online supplemental table S1).

10.1136/gutjnl-2021-324755.supp2 Supplementary data

Stool and saliva (mouthwash) samples were preserved in RNALater and stored at 4°C immediately for 12 hours, then transferred to −20°C for another 24 hours, and then stored at −80°C until DNA extraction. Tumour and non-affected tissue samples were collected during surgery for a subset of individuals, immediately flash-frozen in liquid nitrogen after pathological assessment, and preserved at −80°C. All the samples were shipped on dry ice.

An independent validation population was recruited at the Department of Surgery, University Hospital of Erlangen (32 PDAC and 32 control samples) and Section for Translational Hepatology, Department of Internal Medicine I, Goethe University Clinic, Frankfurt (12 PDAC samples) using the same protocols for biological sample collection, processing and storage. Matched controls were selected from inpatients with a primary diagnosis for hospital admission not related to PDAC risk factors. The study was approved by the local ethics committees (SGI-3–2019, 451_18 B), and written informed consent from study participants was obtained. Clinical data, including disease stage and follow-up data, were retrieved from the clinical records of the hospital charts of the respective patients (online supplemental table S2). Serum CA19-9 levels were analysed by a routine immunoassay (Roche Diagnostics, Germany) following the manufacturer's instructions. Stool samples were preserved in OMNIgene-Gut OM-200 vials (Steinbrenner Laborsysteme GmbH, Germany) and stored at −80°C immediately until DNA extraction.

### Sample processing

Faecal and salivary samples were thawed on ice, aliquoted, and genomic DNA was extracted using the Qiagen Allprep PowerFecal DNA/RNA kit according to the manufacturer’s instructions (Qiagen, Hilden, Germany). Genomic DNA from pancreatic tumorous and non-tumoral tissue samples was extracted using the Qiagen DNeasy blood and tissue kit in a protocol modified from Del Castillo *et al* 26: cells were lysed mechanically (with 5 mm stainless steel beads at 25 Hz for 150 s), followed by lysozyme treatment (20 mg/mL) and protease and RNAse digestion (56°C for 2 h). All samples were randomly assigned to extraction batches. To account for potential bacterial contamination of extraction, polymerase chain reaction (PCR) and sequencing kits, we included negative controls (extraction blanks) with each tissue DNA extraction batch (online supplemental figure 1).

10.1136/gutjnl-2021-324755.supp1 Supplementary data

### 16S rRNA amplicon sequencing

Pancreatic tissue DNA was enriched for 16S rRNA in a preamplification PCR using primers 331F (5’-TCCTACGGGAGGCAGCAGT-3’)^61^ and 979R (5’-GGTTCTKCGCGTTGCWTC-3’).^62^ The cycling conditions consisted of an initial template denaturation at 98°C for 2 min, followed by 30 cycles of denaturation at 98°C for 10 s, annealing at 65°C for 20 s, extension at 72°C for 30 s and a final extension at 72°C for 10 min. This was followed by a size-selective cleanup using SPRIselect magnetic beads (0.8 left-sized; Beckman Coulter, Brea, California, USA). Faecal and salivary DNA were not preamplified.

Targeted amplification of the 16S rRNA V4 region (primer sequences F515 5’-GTGCCAGCMGCCGCGGTAA-3’ and R806 5’-GGACTACHVGGGTWTCTAAT-3’),^63^ was performed using the KAPA HiFi HotStart PCR mix (Roche, Basel, Switzerland) in a two-step barcoded PCR protocol (NEXTflex 16S V4 Amplicon-Seq Kit; Bioo Scientific, Austin, Texas, USA) with minor modifications from the manufacturer’s instructions. PCR products were pooled, purified using size-selective SPRIselect magnetic beads (0.8 left-sized) and then sequenced at 2×250 bp on an Illumina MiSeq (Illumina, San Diego, California, USA) at the Genomics Core Facility, European Molecular Biology Laboratory, Heidelberg.

### 16S rRNA amplicon data processing

Raw reads were quality trimmed, denoised and filtered against chimeric PCR artefacts using DADA2.^64^ The resulting exact amplicon sequence variants (ASVs) were taxonomically classified and mapped to a reference set of operational taxonomic units (OTUs) at 98% sequence similarity using MAPseq.^65^ Reads that did not confidently map to the reference were aligned to bacterial and archaeal secondary structure-aware small subunit rRNA models using Infernal^66^ and clustered into OTUs with 98% average linkage using HPC-CLUST,^67^ as described previously.^68^ As a result, we obtained taxa tables at two resolutions: 100% identical ASVs and 98% open-reference OTUs; unless otherwise indicated, analyses in the main text refer to OTUs.

Count tables were noise filtered by removing samples retaining less than 500 reads and taxa observed in fewer than five samples; this removed 2.5% of total reads from the dataset. For 18 salivary samples, technical replicates were merged after confirming that they strongly correlated with community composition. For pancreatic tissue and tumour samples, ASVs observed in negative control samples were removed, as were reads mapping to known reagent kit contaminants.^33^ After these steps, we retained 308 16S rRNA amplicon samples from 143 subjects for further analyses (130 salivary, 118 faecal, 20 of unaffected pancreatic tissue, 23 of tumour tissue with 17 matching PDAC tissue samples).

### Shotgun metagenomic sequencing

Metagenomic libraries for 212 faecal and 100 salivary samples were prepared using the NEB Ultra II and SPRI HD kits, depending on the concentration of starting material, with a targeted insert size of 350, and sequenced on an Illumina HiSeq 4000 platform (Illumina, San Diego, California, USA) in 2×150 bp paired-end setup to a target depth of 8 Gbp per sample at the Genomics Core Facility, European Molecular Biology Laboratory, Heidelberg. Sequencing statistics for each sample are provided in the associated git repository (https://github.com/psecekartal/PDAC.git). For three salivary and one faecal samples, technical replicates were merged after confirming that they strongly correlated in community composition.

### Metagenome data processing

Metagenomic data were processed using established workflows in NGLess v0.7.1.^69^ Raw reads were quality trimmed (≥45 bp at Phred score ≥25) and filtered against the human genome (version hg19, mapping at ≥90% identity across ≥45 bp). The resulting filtered reads were mapped (≥97% identity across ≥45 bp) against the representative genomes of 5306 species-level genome clusters obtained from the proGenomes database v2.^70^

Taxonomic profiles were obtained using the mOTU profiler v2.5^71^ and filtered to retain only species observed at a relative abundance ≥10^−5^ in ≥2% of samples. Gene functional profiles were obtained from mappings against a global microbioal gene catalogue (GMGCv1, Coelho *et al* 72, http://gmgc.embl.de/), by summarising read counts from eggNOG v4.5^73^ annotations to orthologous groups and KEGG modules. Features with a relative abundance of ≥10^−5^ in ≥15% of samples were retained for further analyses.

### Microbiome data statistical analyses

All data analyses were conducted in the R Statistical Computing framework v3.4 or higher.

Rarefied per-sample taxa diversity (‘alpha diversity’, averaged over 100 rarefaction iterations) was calculated as the effective number of taxa with Hill coefficients of q=0 (ie, taxa richness), q=1 (exponential of Shannon entropy) and q=2 (inverse Simpson index), and evenness measures as ratios thereof. Unless otherwise stated, results in the main text refer to taxa richness. Differences in alpha diversity were tested using analysis of variance (ANOVA) followed by post hoc tests and Benjamini-Hochberg correction, as specified in the main text.

**Figure 1:**
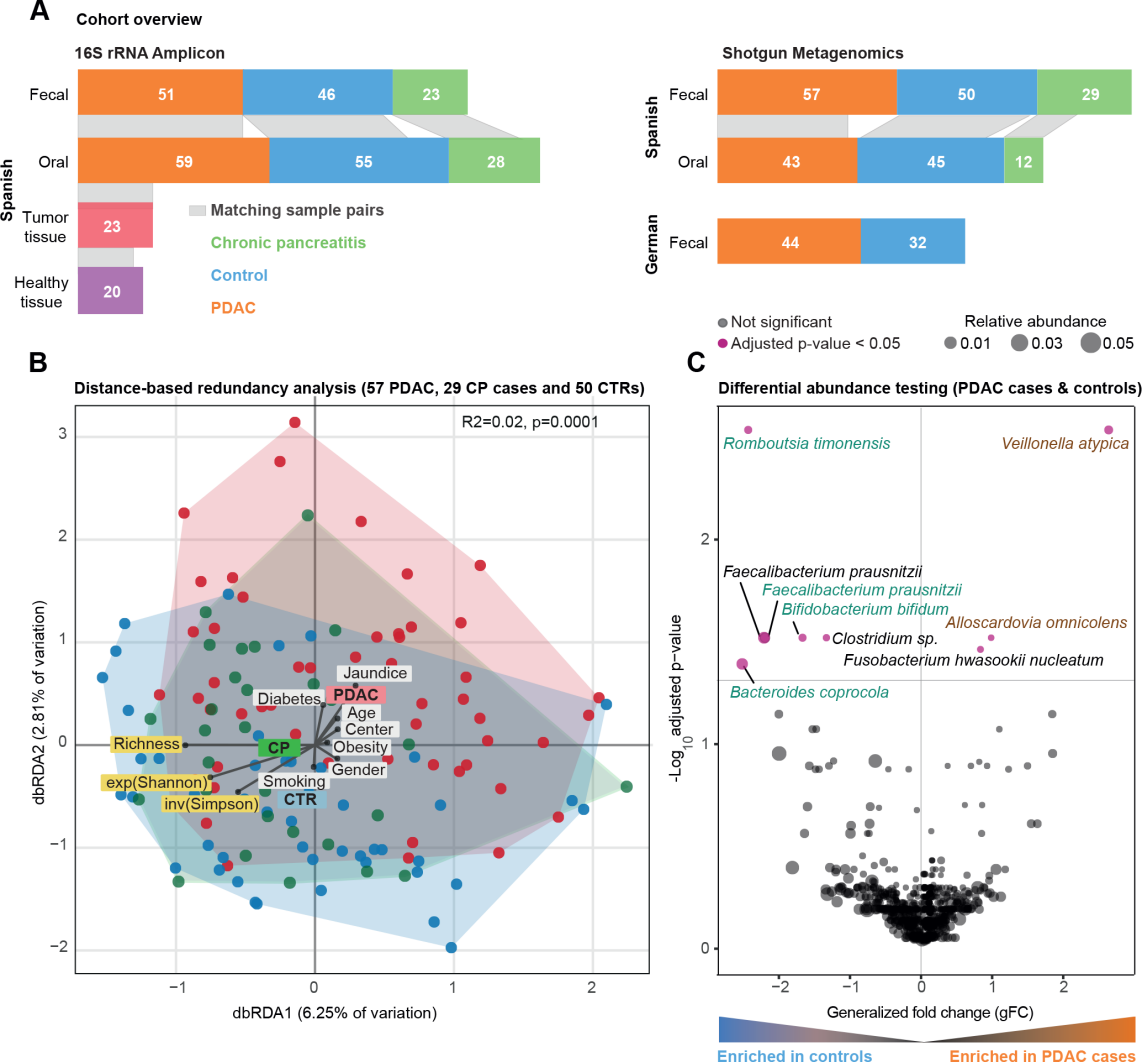
Community analysis of Spanish faecal microbiome data. (A) Study population overview. Grey bands between the bar plots indicate samples of matching body sites within individuals. (B) Bray-Curtis distance-based redundancy analysis (dbRDA) of pancreatic ductal adenocarcinoma (PDAC), chronic pancreatitis (CP) and control (CTR) faecal microbiome data in a Spanish (ES) cohort. PDAC samples are shown as red coloured circles, patients with CP as green and controls as blue. Richness, exponential Shannon (exp(Shannon)) and inverse Simpson (inv(Simpson)) diversity measures are also visualised with arrows similarly to tested metadata variables. The distance of the meta-variable from the centre represents the confounding effect size (see ‘Methods’). (C) Wilcoxon test results of ES faecal microbiome data to test enriched taxa between PDAC and control cases (see ‘Methods’). Y-axis is log10(FDR corrected p values), X-axis is generalised fold change, and dot size represents the relative abundance of a given species. Red dots represent significantly differentially abundant species in either group, while black dots show non-significant species after FDR correction. Green and brown-coloured species are selected in metagenomic model-1 as predictors of PDAC. FDR, false discovery rate.

Between-sample differences in community composition (‘beta diversity’) were quantified as Bray-Curtis dissimilarity on raw or square-root transformed counts, abundance-weighted Jaccard index, and abundance-weighted and unweighted TINA index, as described previously.^74^ Trends between these indices were generally consistent, unless otherwise stated. Results are reported for Bray-Curtis dissimilarities on non-transformed data. Associations of community composition to microbiome-external factors were quantified using the ‘adonis2’ implementation of PERMANOVA and distance-based redundancy analysis in the R package vegan v2.5.^75^ To quantify potentially confounding univariate links between the abundance of individual taxa and subject-specific variables (see main text), we performed either ANOVA or non-parametric Kruskal-Wallis tests, depending on abundance distributions (online supplemental figure 2-3 and online supplemental table S4-S5). Bilirubin levels were measured from blood samples, and jaundice status was confirmed by clinical records. Owing to missing jaundice status for several individuals, values used for further analysis were imputed from existing data (figure 1, online supplemental table S1-S3).

### Multivariable statistical modelling and model evaluation

**Figure 2:**
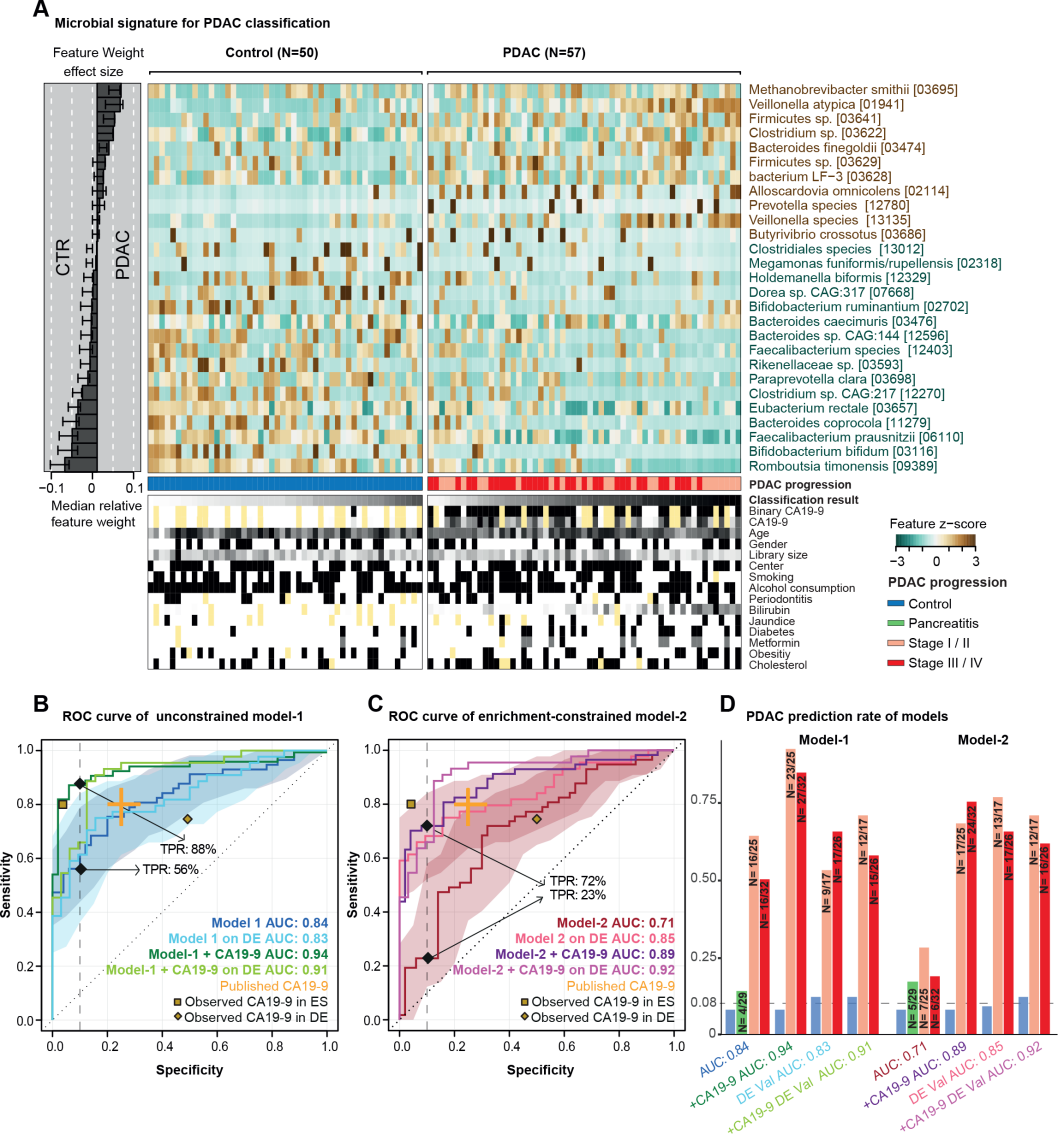
Predictive microbiome signatures of pancreatic ductal adenocarcinoma (PDAC). (A) Normalised abundance of 27 selected species in the faecal microbiome across samples shown as a heat map. The right panel represents the contribution of each selected feature to the overall model-1, and the robustness (the percentage of models in which the feature is included as predictor) of each feature is presented as percentage. Classification scores from cross-validation of each individual and condition for tested meta-variables are displayed at the bottom of the panel, yellow representing missing information. (B–D) Internal cross-validation results of unconstrained model-1 (without feature selection), enrichment-constrained model-2 (constrained to positive features) and combination of carbohydrate antigen (CA)19-9 (using a threshold of 37 μL/mL) with microbial features (see ‘Methods’) are shown as receiver operating characteristic (ROC) curve with 95% CI shaded in corresponding colour. True positive rates (TPRs) are given as a percentage at a 90% specificity cut-off. Validation of all models on an independent German (DE) PDAC test population (n=76) is represented as well. Published CA19-9 accuracy from a meta-study shown in orange. The yellow dots represent observed CA19-9 accuracies in our populations (data available for 33/50 controls (CTRs) and 44/57 patients with PDAC in the Spanish (ES) and for 8/32 CTRs and 44/44 patients with PDAC in the German (DE) population) (D) TPRs of all models at different PDAC progression stages and in addition, the false-positive rate for patients with chronic pancreatitis and controls at a 90% specificity cut-off are shown as bar plots. Stages I and II and stages III and IV are combined owing to the overall low sample size. The number of predicted cases compared with the total is also shown on the top of each bar. DE-Val, German validation population.

In order to train multivariable statistical models for the prediction of pancreatic cancer, we first removed taxa with low overall abundance and prevalence (abundance cut-off point: 0.001). Then, features were normalised by log10 transformation (to avoid infinite values from the logarithm, a pseudo-count of 1e-05 was added to all values) followed by standardisation as centred log-ratio (log.clr). Data were randomly split into test and training sets in a 10 times repeated 10-fold cross-validation. For each test fold, the remaining folds were used as training data to train an L1-regularised (LASSO) logistic regression model^76^ using the implementation within the LiblineaR R package v2.10. 77 The trained model was then used to predict the left-out test set and finally, all predictions were used to calculate the area under the receiver operating characteristics curve (AUROC) (figure 2).

In a second approach, features were filtered within the cross-validation (that is, for each training set) by first calculating the single-feature AUROC and then removing features with an AUROC <0.5, thereby selecting features enriched in PDAC (‘enrichment-constrained’ model).

In order to combine the predictions from the microbiome-based machine learning models with the CA19-9 marker, the coded CA19-9 marker (1 for positive, 0 for negative or not available) was added to the mean predictions from the repeated cross-validation runs, resulting in an OR combination. Alternatively, the AND combination was calculated by multiplying the predictions with the CA19-9 marker. ROC curves and AUROC values were calculated for both combinations using the pROC R package v1.15.^78^ The 95% CI is shaded in corresponding colour and specified in figure legends for each ROC curve.

The trained ES metagenomic classifiers for PDAC were then applied to the DE dataset after applying a data normalisation routine, which selects the same set of features and uses the same normalisation parameters (for example, the mean of a feature for standardisation by using the frozen normalisation functionality in SIAMCAT) as in the normalisation procedure from the ES pancreatic cancer dataset. For this analysis, the cut-off point for the predictions was set to a false-positive rate of 10% among controls in the initial ES PDAC study population (figure 2).

All steps of data preprocessing (filtering and normalisation), model training, predictions and model evaluation were performed using the SIAMCAT R package v.1.5.0^79^ (https://siamcat.embl.de/).

### External validation of the metagenomic classifiers

To assess the disease specificity of the trained models, we obtained predictions for samples from other gut metagenomic datasets (online supplemental table S6) for the full list, including accession numbers). We performed a literature search to identify publicly available datasets of faecal metagenomes in case–control or cohort studies for relevant diseases. For a total set of 25 studies covering 5792 samples across nine disease states, raw sequencing data were downloaded from the European Nucleotide Archive and taxonomically profiled as described above.^35–59^

**Figure 3:**
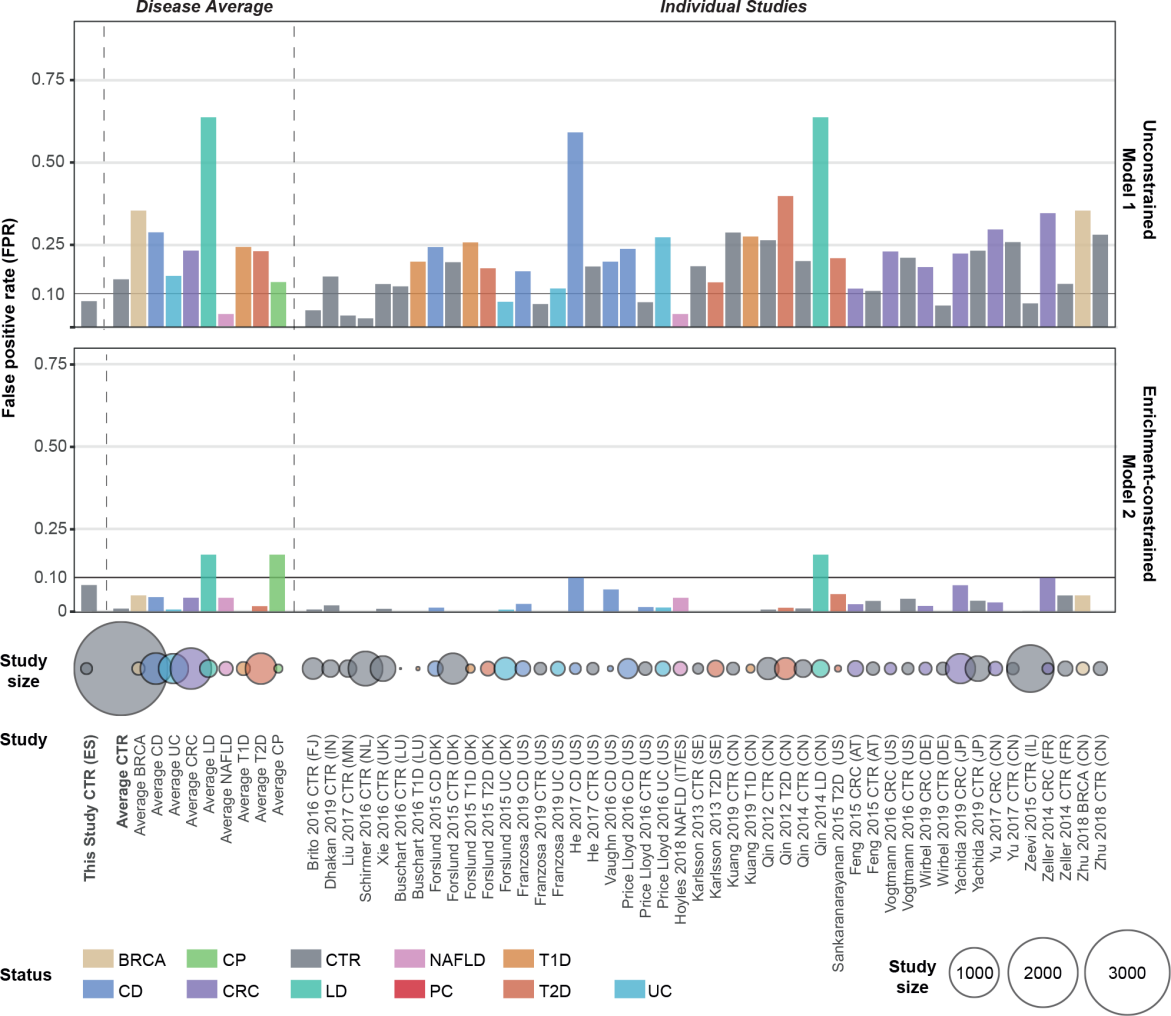
External validation of the disease specificity of pancreatic ductal adenocarcinoma (PDAC) faecal microbiome models. False positive rate (FPR) of metagenomic unconstrained model-1 and enrichment-constrained model-2 in 25 external test sets is shown as a bar plot (see online supplemental table S4 for a list of all studies included). Validation datasets were profiled and normalised in the same way as the initial dataset (see ‘Methods’). Each study was stratified according to health status and models were tested to predict in the given group at a 90% specificity cut-off. A low FPR on metagenomes from patients with other disorders and healthy individuals indicates that the model is specific to PDAC. The number of subjects in each group is displayed as colour coded circles below. BRCA, breast cancer; CRC, colorectal cancer; CD, Crohn’s disease; CP, chronic pancreatitis;, CTR, controls; LD, liver disease; NAFLD, non-alcoholic fatty liver disease; PC, pancreatic cancer; T1D, type 1 diabetes; T2D, type 2 diabetes; UC, ulcerative colitis; ES, Spanish; DE, German.

The trained metagenomic classifiers for PDAC were then applied to each external dataset after applying a data normalisation routine which selects the same set of features and uses the same normalisation parameters (for example, the mean of a feature for standardisation by using frozen normalisation functionality in SIAMCAT) as in the normalisation procedure from the pancreatic cancer dataset. Then, predictions were assessed for disease specificity because high prediction scores for samples from other disease samples would indicate that the classifier relies on general features of dysbiosis in contrast to signals specific to pancreatic cancer, which would not result in elevated false-positive rates on samples from other diseases. For this analysis, the cut-off point for the predictions was set at a false-positive rate of 10% among controls in the initial PDAC study population (figure 3). The effect of age, sex and sequencing depth of 25 populations on prediction score were tested by using the cor.test function (Spearman method) in the car R package v3.0–3.

### Subspecies and strain-level analyses

Metagenomic reads were mapped against species-representative genomes from the proGenomes v1 database^80^ (see above). Microbial single nucleotide variants were called from uniquely mapping reads using metaSNV,^81^ and within-species allele distances between samples were calculated as described previously.^82^ Associations between allele distance and PDAC disease state were quantified using PERMANOVA after stratifying for potential confounders (including sampled body site).

Oral-intestinal transmission of strains was quantified as described previously.^83^ In short, the overlap between microbial single nucleotide variants in salivary and faecal samples within subjects was contrasted with a between-subject background to compute a quantitative oral-faecal transmission score and p value. Associations of species- and subject-specific transmission scores with clinical factors were tested using ANOVA and *post hoc* tests, followed by a Benjamini-Hochberg correction for multiple tests.

### Fluorescence *in situ* hybridisation microscopy

**Figure 4:**
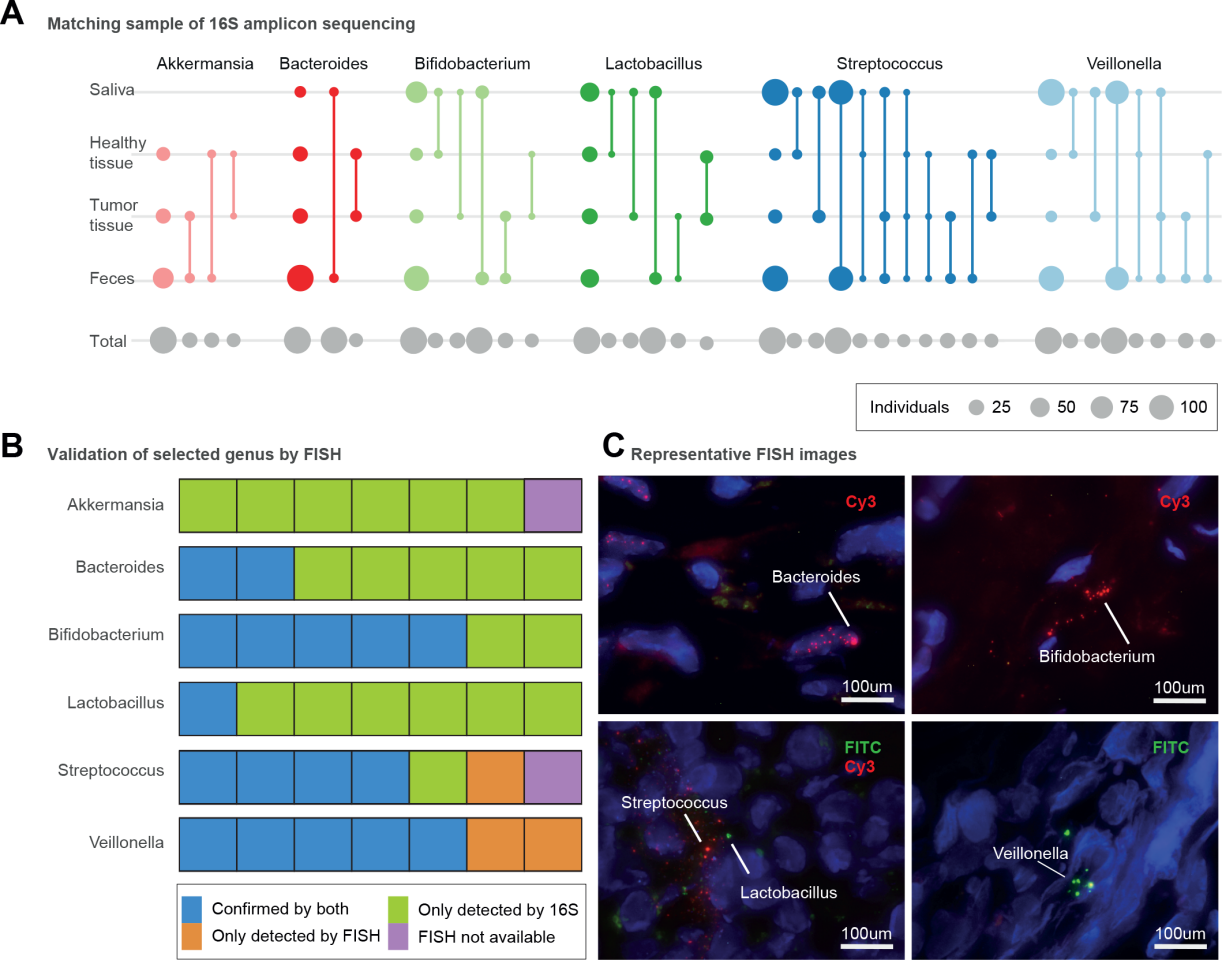
Presence of microbiomes in different sections of the pancreas with different conditions. (A) Presence of different genera in four different body sites including faecal, saliva, pancreatic tumour and healthy tissue samples, as inferred by 16S amplicon data. Circle size corresponds to the total number of subjects available for each comparison (grey, bottom row) or with intra-individually matched amplicon sequence variants (coloured); matched sample types are connected by lines. The first column shows the total number of samples per site in which the genus was detected. (B) Seven selected pancreatic tissue samples (five tumour and two non-tumour) to show bacterial presence/absence with both 16S amplicon and fluorescence *in situ* hybridisation (FISH) methods. Validation of bacterial presence with both 16S amplicon sequencing and FISH is shown in blue. Samples showing bacterial presence according to 16S only are displayed in green. Bacterial presence validated only by FISH is shown in orange, and samples not subjected to FISH validation owing to lack of tissue material are shown in purple. (C) Representative microscopy images for *Bacteroides* (intranuclear, tumour tissue), *Bifidobacterium* (extranuclear, tumour tissue), *Lactobacillus* (extranuclear, non-tumour tissue), *Streptococcus* (extranuclear, non-tumour tissue), *Veillonella* (extranuclear, tumour tissue). Fluorescein isothiocyanate (FITC) and Cy3 fluorescent dyes were used as indicated, and DAPI (4',6,-diamidino-2-phenylindole; blue) was used to label the nucleus.

FISH analyses were performed using probes specifically targeting the 16S rRNA sequence unique to a particular taxon of bacteria (figure 4). All probes were selected based on a literature search and the corresponding taxa are displayed in online supplemental table S7).

Pancreatic tumour and normal pancreas samples were obtained from the pathology department and immediately frozen in liquid nitrogen within less than 30 min of surgical excision. Sterile material was used to dissect the different samples. The minimum size of tissue for freezing was approximately 0.125 cm^3^ (0.5×0.5×0.5 cm). Samples were transferred from the temporary liquid nitrogen transport container and kept in a locked freezer at –80°C. Before analysis they were transported on dry ice, moved to an optimal cutting temperature mould in liquid nitrogen and immediately cut on a cryotome to obtain 10 sections of 3–5 µm each. All material was sterilised with ethanol after each sample handling.

Tissue sections of 5 µm thickness were mounted on positively charged slides (SuperFrost, Thermo Scientific). Briefly, tissues were postfixed in freshly prepared 4% paraformaldehyde. After enhancement of the bacteria wall permeabilisation by lysozyme treatment (10 g/L Tris HCl 6.5M), samples were hybridised for 1 hour at 45°C in the presence of the specific probe in a hybridiser machine (DAKO). Hybridisation was done in 20 µL of hybridisation buffer (20 nM Tris, pH 8.0. 0.9 M NaCl, 0.02% sodium dodecyl sulfate, 30% formamide) added to 100 ng of the probe. Finally, the tissues were washed in washing solution (70% formamide, 10 mM Tris pH7.2 and 01% bovine serum albumin), dehydrated in a series of ethanol samples, air-dried and stained with 0.5 µg/mL DAPI (4',6,-diamidino-2-phenylindole)/antifade solution (Palex Medical). FISH images were captured using a Leica DM5500B microscope with a CCD camera (Photometrics SenSys) connected to a PC running the CytoVision software 7.2 image analysis system (Applied Imaging). Images were analysed blind and scored based on the intensity of the probe signal.

## Results

### PDAC is associated with moderate shifts in microbiome composition when controlling for confounding factors in shotgun metagenomic data

We studied 57 newly diagnosed, treatment-naïve patients with PDAC, 29 patients with chronic pancreatitis (CP), and 50 controls matched for age, gender and hospital. Participants were prospectively recruited from two hospitals in Barcelona and Madrid, Spain, between 2016 and 2018, using the same standards (see subject characteristics in figure 1A and online supplemental table S1-S3 for the clinical data for each subject). We obtained faecal shotgun metagenomes for all subjects and salivary metagenomes for 45 patients with PDAC, 12 with CP, and 43 controls (see ‘Methods’). The analysis workflow is detailed in online supplemental figure 1.

As several PDAC risk factors, such as tobacco smoking, alcohol consumption, obesity or diabetes, are themselves associated with microbiome composition^84^, we first sought to establish potential confounders of microbiome signatures in our study population, in order to adjust analyses accordingly. For a total of 26 demographic and clinical variables, we quantified marginal effects on microbiome community-level diversity (online supplemental table S4). Faecal and salivary microbiome richness (as a proxy for alpha diversity) were not univariately associated with any tested variable, or with PDAC status, when accounting for the most common PDAC risk factors and applying a false discovery rate threshold of 0.05 (online supplemental figure 2, online supplemental table S4).

Microbiome community composition, in contrast, varied with age at diagnosis (PERMANOVA on between-sample Bray-Curtis dissimilarities, R2=0.01, Benjamini-Hochberg-corrected p=0.03), diabetes (R2=0.01, p=0.04) and jaundice status (R2=0.02, p=0.009) in faeces, and with aspirin/paracetamol use (R2=0.02, p=0.04) in saliva, albeit at very low effect sizes (online supplemental table S5). Even though cases and controls were matched for age and sex, we included these factors as strata for subsequent analyses. Under such adjustment, subject disease status was mildly but statistically significantly associated with community composition in faeces (R2=0.02, p=0.001), but not in saliva (R2=0.01, p=0.5) (figure 1B, online supplemental figure 3–4, online supplemental table S5). Indeed, the faecal microbiome composition of patients with PDAC differed from that of both controls (R2=0.02, p≤0.0001) and patients with CP (R2=0.02, p=0.003), although likewise at very small effect sizes.

### High-accuracy metagenomic classifiers capture specific faecal microbiome signatures in patients with PDAC

Having established the presence of a gut microbiome signal for PDAC at the coarse level of overall community composition, we next identified nine species with disease-specific univariate associations (Wilcoxon test of relative abundances in PDAC cases vs controls, Benjamini-Hochberg-corrected p<0.05; see figure 1c). Most prominently, *Veillonella atypica*, *Fusobacterium nucleatum/hwasookii* and *Alloscardovia omnicolens* were enriched in faeces of patients with PDAC, whereas *Romboutsia timonensis, Faecalibacterium prausnitzii, Bacteroides coprocola* and *Bifidobacterium bifidum* species clusters were depleted. In contrast, we did not detect any species with significantly differential abundance in the salivary microbiome when correcting for multiple tests, including previously reported associations, such as *Porphyromonas gingivalis, Aggregatibacter actinomycetemcomitans,* 22 *Neisseria elongata* or *Streptococcus mitis* 18 (online supplemental figure 5).

Among the univariately associated faecal species, several were by themselves moderately predictive of PDAC state (online supplemental figure 5). To coalesce such individual signals into an overarching model, we next built multispecies metagenomic classifiers by fitting LASSO logistic regression models in 10-fold cross-validation (see ‘Methods’). When applying no further constraints, the obtained model discriminated between patients with PDAC and controls with high accuracy in our study population (‘model-1’; AUROC=0.84; Figure 2). The most prominent positive marker species in the model were *Methanobrevibacter smithii*, *Alloscardovia omnicolens*, *Veillonella atypica* and *Bacteroides finegoldii*. We note that by design, LASSO regression selects representative features among inter-correlated sets; therefore, these species may be representatives of larger species sets with highly correlated abundances. None of the 26 demographic and epidemiological variables describing our study population were selected as predictive features by the model, and the microbiome signature was more informative than any other feature (see online supplemental figure 6 and 7). Further, none of these variables were individually associated with the microbial species represented in the model, ruling them out as potential confounders. This indicates that the classifier captured a diagnostic gut microbiome signature of PDAC that is probably independent of other disease risk factors and potential confounders.

An analogous model built to differentiate patients with CP from controls had no predictive power (AUROC=0.5; online supplemental figure 8), consistent with the observation that these groups were compositionally largely indistinguishable. Similarly, no robust PDAC signature was detected for the salivary microbiome (AUROC=0.48; online supplemental figure 9). However, a faecal model to distinguish patients with PDAC from those with CP performed better with an AUC of 0.75, but model robustness was limited by the low sample size in the group with CP (online supplemental figure 8). We further explored predictive associations at the higher resolution of functional microbiome profiles. Models based on the abundances of KEGG modules (online supplemental figure 10) achieved an accuracy of up to AUROC=0.74, but feature selection was likewise not robust across validation folds, as a consequence of fitting a high number of variables (modules) against a limited set of samples. We therefore pursued the species-based classifiers, as they provided stable models.

The initial gut microbiome-based classifier included several species depleted in PDAC relative to controls, such as *Faecalibacterium prausnitzii, Bacteroides coprocola, Bifidobacterium bifidum* or *Romboutsia timonensis* (figure 2B). For some of these species, it was previously suggested that depletion is linked to intestinal inflammation, in general, rather than to specific diseases.^85^ We therefore retrained a classifier with the constraint that positively associated (enriched) microbial features were exclusively selected in each cross-validation fold. The resulting enrichment-constrained model (model-2) discerned patients with PDAC with an accuracy of AUROC=0.71. The difference with the unconstrained model, model-1, was mostly attributable to a penalty on sensitivity—that is, a decrease in confident detections of patients with PDAC, in line with expectations when training on sparse data.

### Combination of metagenomic classifiers with antigen CA19-9 levels increases accuracy

Blood serum levels of the antigen CA19-9 are routinely used to monitor PDAC progress,^86 87^ but have also been suggested as a potential marker for early diagnosis of PDAC, although with moderate reported sensitivity (0.80, 95% CI 0.72 to 0.86) and specificity (0.75, 95% CI 0.68 to 0.80).^12^ CA19-9 serum levels were available for a subset of 77 individuals (33/50 controls and 44/57 patients with PDAC) in our Spanish population (online supplemental figure S11). Given that CA19-9 is directly secreted by tumours, we hypothesised that the readouts provided by CA19-9 serum levels and by our microbiome classifiers were complementary, and that their combination could improve the accuracy of PDAC prediction. Indeed, accounting for CA19-9 increased the accuracy of our unconstrained model-1 from AUROC=0.84 to 0.94, driven mostly by an increase in sensitivity (figure 2B). More strikingly, when we amended the enrichment-constrained model-2 with CA19-9 information, we observed a large increase in accuracy from AUC=0.71 to 0.89, likewise driven by a significant improvement in sensitivity, thereby essentially abolishing the performance penalty relative to model-1 (figure 2C, online supplemental figure S11). There was no significant bias towards higher CA19-9 levels in later disease stages in either the ES or DE populations (online supplemental figure S11).

Our Spanish study population included 25 patients with PDAC in early disease stages (T1, T2) and 32 subjects in later stages (T3, T4). Disease stage did not affect the performance of either microbiome-based model (figure 2D); in particular, recall was not biased towards later stages.

### Performance of metagenome-based classifiers generalises to independent validation cohorts

To test whether the observed microbiome signatures generalise beyond our focal Spanish study population, we next challenged our models in two validation scenarios. First, we tested prediction accuracy in an independent study population of 44 patients with PDAC and 32 matched controls, recruited from two hospitals in Erlangen and Frankfurt am Main, Germany (see figure 1, Methods and online supplemental table S3), with the samples being processed identically to those of the Spanish population. On this DE validation population, both the unconstrained model-1 (figure 2B) and the enrichment-constrained model-2 (figure 2C) performed with comparable or indeed superior accuracies to the training population, both with and without complementation by CA19-9 levels, and with similar trends across disease stages (figure 2D).

Next, to confirm that our metagenomic classifiers captured PDAC-specific signatures, rather than unspecific, more general disease-associated variation, we further validated them against independent, external metagenomic datasets on various health conditions. In total, we classified 5792 publicly available gut metagenomes from 25 studies across 18 countries, including subjects with CP (this study), type 1 or type 2 diabetes, colorectal cancer, breast cancer, liver diseases, non-alcoholic fatty liver disease, including Crohn’s disease and ulcerative colitis, as well as healthy controls (figure 3 and online supplemental table S6).

When tuned to 90% specificity (allowing for 10% false positive predictions) in our focal ES study population, the unconstrained model-1 showed a recall of 56% of patients with PDAC in the ES population and 48% in the DE validation population (with 6% false-positive rate), and up to 64% when complemented with information on CA19-9 levels (available for 8/32 controls and 43/44 patients with cases in the DE cohort). The disease specificity of model-1, however, was limited, with predictions of PDAC state for 15% of control subjects on average across all external datasets. Most of these false positive calls were observed in two Chinese populations of patients with Crohn’s disease^48^ or liver cirrhosis.^44^ Crohn’s disease has been associated with depletion signatures similar to those observed in our model (in particular of *F. prausnitzii,* 88) whereas liver diseases share some physiological characteristics with impaired pancreas function. However, all other liver disease and Crohn's disease sets showed lower false detection rates, indicating that the effect was probably attributable, in part, to technical and demographic effects between studies. Indeed, we note that subjects in these two Chinese study populations were significantly younger than our populations (50±11 years for Qin_2014; 28.5±8 years for He_2017; 70±12 y ears for our ES population). This age effect was systematic: across all validation sets, PDAC prediction scores were associated with subject age (ANOVA p=0.007; ρ_Spearman_ = 0.16), as well as with the sex of the subject (p<10^-6;^) and sequencing depth (p=0.0008; ρ_Spearman_ = 0.1) (online supplemental figure S12, online supplemental table S6).

The enrichment-constrained model-2 showed lower detection rates in patients with PDAC in both populations, although recall was reinstated for CA19-9 combined models. Model-2 was highly specific for PDAC with, on average, just 0–5% PDAC predictions in almost all external populations, at a maximum of 17% predictions among the aforementioned^44^ population with liver disease. In particular, the detected microbiome signatures were also robust against misclassification of patients with type 2 diabetes (<2% false-positive rate); this is relevant to potential screening applications, as these patients are a major PDAC risk group (figure 3).

### PDAC harbours characteristic bacteria, consistent with oral and gut microbiome communities

Alterations in pancreatic secretion, as a consequence of tumour growth in the pancreatic duct, can affect digestive function and may thus plausibly underlie characteristic gut microbiome signatures, such as those described above. This would imply that PDAC progression can indirectly cause microbiome shifts (ie, reverse causation). In addition, the pancreatic duct directly communicates with the duodenum, providing an anatomical link for bacteria^25 30 89^ and fungi^34^ to colonise the pancreas and contribute to carcinogenesis.^31^

We therefore hypothesised that several gut microbial taxa associated with PDAC should be detectable in pancreatic tumours. We taxonomically profiled all faecal and salivary samples, as well as biopsies of tumours (n=23) and adjacent healthy pancreatic tissue (n=20) of patients with PDAC from our study population using 16S rRNA amplicon sequencing, applying strict filters to exclude putative reagent contaminants often seen in samples of low bacterial biomass^33 90^ (see ‘Methods’). We observed a surprisingly rich and diverse pancreas microbiome, with at least 13 bacterial genera present in ≥25% of samples, prominently including taxa with characteristic PDAC signatures in the faecal microbiome^91^ (figure 4 A, online supplemental figure 13). Among these, *Lactobacillus* spp, *Akkermansia muciniphila* and *Bacteroides* spp were enriched in tumours relative to non-tumour pancreatic tissue (Wilcoxon test, false discovery rate-corrected p<0.006).

In a subset of five tumour and two non-tumoral pancreatic tissue samples, we could further verify the prevalence of *Akkermansia* spp, *Lactobacillus* spp, *Bifidobacterium* spp, *Veillonella* spp, *Bacteroides* spp and *Streptococcus* spp using FISH assays with genus-specific primers (online supplemental figure 4, online supplemental table S7). Generally, amplicon and FISH data were concordant, though amplicon-based detection appeared more sensitive probably due to the amount of tissue analysed. Intriguingly, however, *Akkermansia* spp, although observed by amplicon sequencing in 26/30 subjects, were not detectable using FISH in any of the tested samples (figure 4B–C, online supplemental figure 14).

### Links between oral, intestinal and pancreatic microbiomes

We next traced exact amplicon sequence variants (ASVs) across salivary, faecal, tumour and healthy tissue samples within subjects (figure 4A), at the highest taxonomic resolution attainable using 16S rRNA data. *Veillonella* spp, characteristically enriched in stool of patients with PDAC, were highly prevalent in both salivary (100% of subjects) and faecal (87.5%) samples across the entire study population, while oral and faecal types also matched tumour and non-tumour tissue ASVs. Interestingly, we found no intraindividual match in *Veillonella* ASVs between tumour and adjacent tissue samples, indicating that tumor-dwelling *Veillonella* spp may be distinct from those in healthy tissue. In addition, our data confirm previous reports that *Lactobacillus* spp^26^ and *Bifidobacterium* spp^25^ are present in both PDAC tumour and non-tumour tissue. For both genera, we found that tumour types corresponded to either oral or faecal ASVs, but not both, whereas no ASVs from healthy tissue were matched with faecal samples, indicating that distinct pancreatic subpopulations may be linked to the mouth and the gut.

Using paired salivary and faecal shotgun metagenomes, we further confirmed that strains of faecal PDAC-associated microbes may be sourced from the oral cavity (online supplemental results).

## Discussion

Early detection of PDAC remains a formidable challenge, at the heart of ongoing efforts to mitigate the burden of this cancer. Currently, the sole FDA-approved biomarker for PDAC is serum CA19-9, mostly used for disease monitoring rather than screening, due to inherent limits of sensitivity and specificity: CA19-9 levels can be elevated in several conditions unrelated to pancreatic cancer, while subjects lacking the Lewis-A antigen do not produce CA19-9 at all.^10–12^ Small-scale studies have proposed PDAC markers based on pancreatic tissue,^5^ urine^6 7^ and blood serum^8 9^ with limited applicability. Yet there are currently no screening tools for PDAC in the clinic—in particular, for early disease stages.

In a prospectively recruited study population of newly diagnosed, treatment-naïve patients and matched controls for whom oral, faecal and tissue microbiomes were analysed (figure 1A), we developed metagenomic classifiers that robustly and accurately predict PDAC solely based on characteristic faecal microbial species (figure 2). PDAC signatures captured by our multispecies models were orthogonal to well-established PDAC risk factors (figures 1B and 2A). This suggests that, in practice, the faecal microbiome may be used to screen for PDAC, complementary to other testable markers, with added diagnostic accuracy in combined tests, as has been proposed for colorectal cancer.^39^ Indeed, a combination of our microbiome classifiers with CA19-9 data, available for a subset of our population, significantly enhanced the accuracy of PDAC detection (figure 2B–D).

Previous studies have explored links between PDAC and the oral^18–22 26 92 93^ or faecal^23 24^ microbiome at the limited taxonomic resolution of 16S rRNA sequencing, but provided conflicting reports regarding the association patterns of individual taxa, probably due to heterogeneous experimental and analytical approaches. The non-availability of raw sequence and patient-level clinical data for several PDAC datasets has made comparisons between studies challenging, and thus a consensus on PDAC-associated microbiome signatures has so far failed to emerge. Several previously reported univariate PDAC associations of oral taxa including *P. gingivalis, A. actinomycetemcomitans*, *S. thermophilus* and *Fusobacterium* spp were not confirmed in our study population (online supplemental figure 4); we generally did not observe any salivary PDAC signature either for individual species or for multispecies models.

We carefully checked our analyses for demographic, lifestyle, and clinical confounders, as these can show stronger microbiome associations than disease states.^84^ We moreover validated our metagenomic classifiers against the independently sampled, yet consistently processed, DE population (figure 2B–D) and against external populations of various health states from 25 different studies (n=5792)^35–59^ (figure 3). Both confounder control and external validation are essential when assessing the disease specificity of predictive models, in particular for diseases like PDAC with low incidence in the general population. This was confirmed in our analyses: among our two metagenomic classifiers, model-1 showed a high accuracy of AUROC=0.84 in our ES study population, driven by a high recall of patients with PDAC. However, model-1 showed only limited disease specificity in external validations, capturing non-specific species depletion signals discriminative between cases and controls in our population, but also shared by subjects with other diseases. These included generic inflammation signatures—for example, a depletion of *F. prausnitzii, E. rectale* or *B. bifidum*. Published metagenomic classifiers for various diseases, and in particular previously reported signatures for PDAC, share similar limitations: highly tuned accuracy on the focal population, but non-specific features shared with other diseases. This lack of specificity limits their translation into clinical practice. In contrast, our model-2, constrained to PDAC-enriched features, achieved only moderate accuracy within our populations (AUC=0.71 on ES, AUC=0.85 on DE) due to a penalty on sensitivity, but was highly PDAC-specific with very low false prediction rates in external populations, including known PDAC risk groups such as those with type 2 diabetes. In particular, PDAC-enriched features in both model-1 and model-2 showed little overlap with characteristic faecal microbiome features for other cancer types, such as colorectal cancer, indicating that a combination of our microbiome models with CA19-9 levels (highly sensitive, but not specific to PDAC) is promising. We note that the residual false positive rate among external populations may partly be due to technical heterogeneity, as all external populations were sampled and processed using independent protocols, and that univariate PDAC associations of individual species may be informative, but not disease-specific (Supplementary Discussion). The panel of PDAC-enriched species in model-2 thus shows potential for microbiome-based PDAC screening, given that a combination with complementary information on serum CA19-9 significantly increased accuracy (AUC=0.89 and 0.92).

Our models showed comparable performance across PDAC disease stages, with no bias towards later stages (figure 2B–D). This indicates that characteristic microbiome signatures emerge early during progression of the disease and that the faecal microbiome can serve for the early detection of PDAC.

Our data are strictly observational and cross-sectional. Nevertheless, there are strong indications that the identified faecal microbiome shifts are not merely a consequence of impaired pancreatic function or systemic effects thereof, although indirect effects cannot be ruled out. Several taxa could be traced between the gut and pancreas, with univariate enrichment in tumours relative to adjacent healthy tissue, indicating direct associations of PDAC with the gut microbiome. We confirmed previous observations^25 30 31 89 91^ that the human pancreas harbours a microbiome, both by amplicon sequencing, and by FISH for the most comprehensive panel of taxa to date (figure 4). Pancreatic tissue and tumours contain only low bacterial biomass and are therefore prone to contamination in 16S rRNA amplicon data^33^, whereas FISH testing requires specific hypotheses, so a comprehensive cataloguing of the healthy and diseased pancreatic microbiome composition is still emerging. In our study, we carefully filtered our dataset against known kit contaminants and confirmed the presence of various key genera using FISH assays. We moreover observed an intraindividual overlap of exact amplicon sequence variants between oral, faecal and tissue samples, confirming a shared presence across multiple sites for several species at the highest attainable taxonomic resolution for amplicon data.

Faecal populations of characteristic PDAC-associated taxa could thus be traced back to pancreatic tumours. Similarly, we observed significantly increased levels of oral-intestinal strain transmission in patients with PDAC, in particular of PDAC signature taxa, indicating that these may be sourced intraindividually, from the oral cavity (online supplemental results). These findings suggest that the oral, intestinal and pancreatic microbiomes may be intricately linked, and that multibody site study designs such as presented here will be necessary to disentangle their respective roles and interactions in PDAC aetiology.

In summary, the described faecal microbiome signatures enabled robust metagenomic classifiers for PDAC detection at high disease specificity, complementary to existing markers, and with potential towards cost-effective PDAC screening and monitoring. Furthermore, in view of previous reports on microbe-mediated pancreatic carcinogenesis in murine models and humans,^25 30 94^ we believe that the presented panel of PDAC-associated bacterial species may be relevant beyond their use for diagnosis, providing promising future entry points for disease prevention and therapeutic intervention.

## Data Availability

Data are available in a public, open access repository. All data relevant to the study are included in the article or uploaded as supplementary information. The raw sequencing data for the samples are made available in the European Nucleotide Archive (ENA) under the study identifiers PRJEB38625 and PRJEB42013. Metadata for these samples are available as Supplementary Tables S1 and S2. Filtered taxonomic and functional profiles used as input for the statistical modelling pipeline are available in Supplementary Data S1 and S2. Analysis code and results available under https://github.com/psecekartal/PDAC.git.
