# Supplementary material for: A faecal microbiota signature with high specificity for pancreatic cancer
Source: Gut. 2022 Mar 8;71(7):1359–72. doi: 10.1136/gutjnl-2021-324755 (PMC9185815; doi:10.1136/gutjnl-2021-324755)
Supplement: Supplementary data [file gutjnl-2021-324755supp002.pdf]

Supplementary Table S1: ES Cohort Details

sample_alias	experiment_name	experiment_type	instrument_model	replicate	insert_size	read_length	read_count	environment_material	timepoint
MMPC-3103102-ST-0	MMPC35551931ST	metagenome	Illumina HiSeq 4000	0	350	150	11417135	feces [ENVO:00002003]	0
MMPC-3103103-ST-0	MMPC41376408ST	metagenome	Illumina HiSeq 4000	0	350	150	22532306	feces [ENVO:00002003]	0
MMPC-3103106-ST-0	MMPC59659730ST	metagenome	Illumina HiSeq 4000	0	350	150	22296806	feces [ENVO:00002003]	0
MMPC-3103107-ST-0	MMPC96048560ST	metagenome	Illumina HiSeq 4000	0	350	150	15812641	feces [ENVO:00002003]	0
MMPC-3103107-ST-0	MMPW24306397ST	metagenome	Illumina HiSeq 4000	0	350	150	562381156	feces [ENVO:00002003]	0
MMPC-3103108-ST-0	MMPC42296680ST	metagenome	Illumina HiSeq 4000	0	350	150	12998378	feces [ENVO:00002003]	0
MMPC-3103109-ST-0	MMPC49776422ST	metagenome	Illumina HiSeq 4000	0	350	150	24362813	feces [ENVO:00002003]	0
MMPC-3103109-ST-0	MMPW76972986ST	metagenome	Illumina HiSeq 4000	0	350	150	597499733	feces [ENVO:00002003]	0
MMPC-3103111-ST-0	MMPC19228810ST	metagenome	Illumina HiSeq 4000	0	350	150	15320194	feces [ENVO:00002003]	0
MMPC-3103112-ST-0	MMPW28983031ST	metagenome	Illumina HiSeq 4000	0	350	150	514088417	feces [ENVO:00002003]	0
MMPC-3103112-ST-0	MMPC45272194ST	metagenome	Illumina HiSeq 4000	0	350	150	19734216	feces [ENVO:00002003]	0
MMPC-3103114-ST-0	MMPC36793964ST	metagenome	Illumina HiSeq 4000	0	350	150	12388494	feces [ENVO:00002003]	0
MMPC-3103115-ST-0	MMPC92286162ST	metagenome	Illumina HiSeq 4000	0	350	150	12858226	feces [ENVO:00002003]	0
MMPC-3103116-ST-0	MMPC23673679ST	metagenome	Illumina HiSeq 4000	0	350	150	13475213	feces [ENVO:00002003]	0
MMPC-3103116-ST-0	MMPW31043630ST	metagenome	Illumina HiSeq 4000	0	350	150	540784922	feces [ENVO:00002003]	0
MMPC-3103117-ST-0	MMPW39199901ST	metagenome	Illumina HiSeq 4000	0	350	150	551836962	feces [ENVO:00002003]	0
MMPC-3103117-ST-0	MMPC53747560ST	metagenome	Illumina HiSeq 4000	0	350	150	19414746	feces [ENVO:00002003]	0
MMPC-3103118-ST-0	MMPC76278395ST	metagenome	Illumina HiSeq 4000	0	350	150	23770034	feces [ENVO:00002003]	0
MMPC-3103119-ST-0	MMPC92856474ST	metagenome	Illumina HiSeq 4000	0	350	150	24862116	feces [ENVO:00002003]	0
MMPC-3103120-ST-0	MMPC41385323ST	metagenome	Illumina HiSeq 4000	0	350	150	17450417	feces [ENVO:00002003]	0
MMPC-3103121-ST-0	MMPC12800445ST	metagenome	Illumina HiSeq 4000	0	350	150	20514446	feces [ENVO:00002003]	0
MMPC-3103124-ST-0	MMPC10431544ST	metagenome	Illumina HiSeq 4000	0	350	150	34540047	feces [ENVO:00002003]	0
MMPC-3103124-ST-0	MMPW97100755ST	metagenome	Illumina HiSeq 4000	0	350	150	616136180	feces [ENVO:00002003]	0
MMPC-3103125-ST-0	MMPC29682684ST	metagenome	Illumina HiSeq 4000	0	350	150	29767506	feces [ENVO:00002003]	0
MMPC-3103126-ST-0	MMPC51115713ST	metagenome	Illumina HiSeq 4000	0	350	150	38401273	feces [ENVO:00002003]	0
MMPC-3103127-ST-0	MMPC99615156ST	metagenome	Illumina HiSeq 4000	0	350	150	11813347	feces [ENVO:00002003]	0
MMPC-3103129-ST-0	MMPC45114789ST	metagenome	Illumina HiSeq 4000	0	350	150	43078526	feces [ENVO:00002003]	0
MMPC-3103202-ST-0	MMPC52359973ST	metagenome	Illumina HiSeq 4000	0	350	150	18061157	feces [ENVO:00002003]	0
MMPC-3103203-ST-0	MMPW21548424ST	metagenome	Illumina HiSeq 4000	0	350	150	542893761	feces [ENVO:00002003]	0
MMPC-3103203-ST-0	MMPC73667956ST	metagenome	Illumina HiSeq 4000	0	350	150	18138792	feces [ENVO:00002003]	0
MMPC-3103206-ST-0	MMPC47668641ST	metagenome	Illumina HiSeq 4000	0	350	150	28939256	feces [ENVO:00002003]	0
MMPC-3103207-ST-0	MMPC69211422ST	metagenome	Illumina HiSeq 4000	0	350	150	19540088	feces [ENVO:00002003]	0
MMPC-3103208-ST-0	MMPC78378767ST	metagenome	Illumina HiSeq 4000	0	350	150	18782020	feces [ENVO:00002003]	0
MMPC-3103209-ST-0	MMPC75057555ST	metagenome	Illumina HiSeq 4000	0	350	150	41063683	feces [ENVO:00002003]	0
MMPC-3103211-ST-0	MMPC63233635ST	metagenome	Illumina HiSeq 4000	0	350	150	34235593	feces [ENVO:00002003]	0
MMPC-3103211-ST-0	MMPW67722444ST	metagenome	Illumina HiSeq 4000	0	350	150	577789449	feces [ENVO:00002003]	0
MMPC-3103212-ST-0	MMPC25016485ST	metagenome	Illumina HiSeq 4000	0	350	150	13684247	feces [ENVO:00002003]	0
MMPC-3103214-ST-0	MMPC82779175ST	metagenome	Illumina HiSeq 4000	0	350	150	15530149	feces [ENVO:00002003]	0
MMPC-3103215-ST-0	MMPC64434180ST	metagenome	Illumina HiSeq 4000	0	350	150	28178046	feces [ENVO:00002003]	0
MMPC-3103216-ST-0	MMPC37499694ST	metagenome	Illumina HiSeq 4000	0	350	150	35725071	feces [ENVO:00002003]	0
MMPC-3103217-ST-0	MMPC59151585ST	metagenome	Illumina HiSeq 4000	0	350	150	47396825	feces [ENVO:00002003]	0
MMPC-3103218-ST-0	MMPC12849395ST	metagenome	Illumina HiSeq 4000	0	350	150	48769925	feces [ENVO:00002003]	0
MMPC-3103219-ST-0	MMPC96275284ST	metagenome	Illumina HiSeq 4000	0	350	150	21659974	feces [ENVO:00002003]	0
MMPC-3103220-ST-0	MMPC32834082ST	metagenome	Illumina HiSeq 4000	0	350	150	17111873	feces [ENVO:00002003]	0
MMPC-3103221-ST-0	MMPC69837834ST	metagenome	Illumina HiSeq 4000	0	350	150	9569359	feces [ENVO:00002003]	0
MMPC-3103223-ST-0	MMPC36232351ST	metagenome	Illumina HiSeq 4000	0	350	150	59219214	feces [ENVO:00002003]	0
MMPC-3103224-ST-0	MMPC21449543ST	metagenome	Illumina HiSeq 4000	0	350	150	31704480	feces [ENVO:00002003]	0
MMPC-3103225-ST-0	MMPC63481302ST	metagenome	Illumina HiSeq 4000	0	350	150	38774804	feces [ENVO:00002003]	0
MMPC-3103226-ST-0	MMPC22175846ST	metagenome	Illumina HiSeq 4000	0	350	150	44695903	feces [ENVO:00002003]	0
MMPC-3103227-ST-0	MMPC17251241ST	metagenome	Illumina HiSeq 4000	0	350	150	44644402	feces [ENVO:00002003]	0
MMPC-3103228-ST-0	MMPC16651671ST	metagenome	Illumina HiSeq 4000	0	350	150	45156079	feces [ENVO:00002003]	0
MMPC-3103229-ST-0	MMPC59362655ST	metagenome	Illumina HiSeq 4000	0	350	150	42996294	feces [ENVO:00002003]	0
MMPC-3103302-ST-0	MMPC88343716ST	metagenome	Illumina HiSeq 4000	0	350	150	26171451	feces [ENVO:00002003]	0
MMPC-3103303-ST-0	MMPC67317135ST	metagenome	Illumina HiSeq 4000	0	350	150	41987974	feces [ENVO:00002003]	0
MMPC-3103306-ST-0	MMPC75070829ST	metagenome	Illumina HiSeq 4000	0	350	150	46219614	feces [ENVO:00002003]	0
MMPC-3103307-ST-0	MMPC93358150ST	metagenome	Illumina HiSeq 4000	0	350	150	41266766	feces [ENVO:00002003]	0
MMPC-3103308-ST-0	MMPC95108539ST	metagenome	Illumina HiSeq 4000	0	350	150	51296849	feces [ENVO:00002003]	0
MMPC-3103309-ST-0	MMPC68695449ST	metagenome	Illumina HiSeq 4000	0	350	150	16713617	feces [ENVO:00002003]	0
MMPC-3103311-ST-0	MMPC79169108ST	metagenome	Illumina HiSeq 4000	0	350	150	29917194	feces [ENVO:00002003]	0
MMPC-3103312-ST-0	MMPC95991337ST	metagenome	Illumina HiSeq 4000	0	350	150	40108122	feces [ENVO:00002003]	0
MMPC-3103314-ST-0	MMPC82784238ST	metagenome	Illumina HiSeq 4000	0	350	150	49888210	feces [ENVO:00002003]	0
MMPC-3103315-ST-0	MMPC35131016ST	metagenome	Illumina HiSeq 4000	0	350	150	39883458	feces [ENVO:00002003]	0
MMPC-3103316-ST-0	MMPC52740890ST	metagenome	Illumina HiSeq 4000	0	350	150	33343541	feces [ENVO:00002003]	0
MMPC-3103317-ST-0	MMPC48152369ST	metagenome	Illumina HiSeq 4000	0	350	150	48281105	feces [ENVO:00002003]	0
MMPC-3103318-ST-0	MMPC93983194ST	metagenome	Illumina HiSeq 4000	0	350	150	38098451	feces [ENVO:00002003]	0
MMPC-3103319-ST-0	MMPC11113549ST	metagenome	Illumina HiSeq 4000	0	350	150	40759382	feces [ENVO:00002003]	0
MMPC-3103319-ST-0	MMPC50507232ST	metagenome	Illumina HiSeq 4000	0	350	150	48108766	feces [ENVO:00002003]	0
MMPC-3103320-ST-0	MMPC77686266ST	metagenome	Illumina HiSeq 4000	0	350	150	41439816	feces [ENVO:00002003]	0
MMPC-3103321-ST-0	MMPC56485647ST	metagenome	Illumina HiSeq 4000	0	350	150	55325188	feces [ENVO:00002003]	0
MMPC-3103324-ST-0	MMPC71307280ST	metagenome	Illumina HiSeq 4000	0	350	150	28830324	feces [ENVO:00002003]	0
MMPC-3103325-ST-0	MMPC99058548ST	metagenome	Illumina HiSeq 4000	0	350	150	31073638	feces [ENVO:00002003]	0
MMPC-3103326-ST-0	MMPC16382508ST	metagenome	Illumina HiSeq 4000	0	350	150	34469441	feces [ENVO:00002003]	0
MMPC-3103327-ST-0	MMPC23660755ST	metagenome	Illumina HiSeq 4000	0	350	150	41089562	feces [ENVO:00002003]	0
MMPC-3103329-ST-0	MMPC29268878ST	metagenome	Illumina HiSeq 4000	0	350	150	50747715	feces [ENVO:00002003]	0
MMPC-3103330-ST-0	MMPC96910089ST	metagenome	Illumina HiSeq 4000	0	350	150	15562457	feces [ENVO:00002003]	0
MMPC-3109101-ST-0	MMPC14211565ST	metagenome	Illumina HiSeq 4000	0	350	150	74174779	feces [ENVO:00002003]	0
MMPC-3109101-ST-0	MMPW29397139ST	metagenome	Illumina HiSeq 4000	0	350	150	468087742	feces [ENVO:00002003]	0
MMPC-3109102-ST-0	MMPC20689138ST	metagenome	Illumina HiSeq 4000	0	350	150	42313248	feces [ENVO:00002003]	0
MMPC-3109103-ST-0	MMPW82409611ST	metagenome	Illumina HiSeq 4000	0	350	150	448012729	feces [ENVO:00002003]	0
MMPC-3109103-ST-0	MMPC61634104ST	metagenome	Illumina HiSeq 4000	0	350	150	28553975	feces [ENVO:00002003]	0
MMPC-3109104-ST-0	MMPC53642802ST	metagenome	Illumina HiSeq 4000	0	350	150	12302431	feces [ENVO:00002003]	0
MMPC-3109105-ST-0	MMPC83679680ST	metagenome	Illumina HiSeq 4000	0	350	150	18019953	feces [ENVO:00002003]	0
MMPC-3109106-ST-0	MMPC21059026ST	metagenome	Illumina HiSeq 4000	0	350	150	15660594	feces [ENVO:00002003]	0
MMPC-3109108-ST-0	MMPC29596062ST	metagenome	Illumina HiSeq 4000	0	350	150	15449487	feces [ENVO:00002003]	0
MMPC-3109109-ST-0	MMPC79962166ST	metagenome	Illumina HiSeq 4000	0	350	150	45702011	feces [ENVO:00002003]	0
MMPC-3109110-ST-0	MMPC84979047ST	metagenome	Illumina HiSeq 4000	0	350	150	51915806	feces [ENVO:00002003]	0
MMPC-3109111-ST-0	MMPC54944306ST	metagenome	Illumina HiSeq 4000	0	350	150	9093837	feces [ENVO:00002003]	0
MMPC-3109112-ST-0	MMPC36137610ST	metagenome	Illumina HiSeq 4000	0	350	150	11591475	feces [ENVO:00002003]	0

MMPC-3109114-ST-0	MMPC36075929ST	metagenome	Illumina HiSeq 4000	0	350	150	29343109	feces [ENVO:00002003]	0
MMPC-3109115-ST-0	MMPC40565253ST	metagenome	Illumina HiSeq 4000	0	350	150	24643317	feces [ENVO:00002003]	0
MMPC-3109116-ST-0	MMPC67417777ST	metagenome	Illumina HiSeq 4000	0	350	150	45871936	feces [ENVO:00002003]	0
MMPC-3109117-ST-0	MMPW14699153ST	metagenome	Illumina HiSeq 4000	0	350	150	560238359	feces [ENVO:00002003]	0
MMPC-3109117-ST-0	MMPC49013463ST	metagenome	Illumina HiSeq 4000	0	350	150	23300387	feces [ENVO:00002003]	0
MMPC-3109118-ST-0	MMPC65662501ST	metagenome	Illumina HiSeq 4000	0	350	150	15601130	feces [ENVO:00002003]	0
MMPC-3109119-ST-0	MMPC40914520ST	metagenome	Illumina HiSeq 4000	0	350	150	22964094	feces [ENVO:00002003]	0
MMPC-3109120-ST-0	MMPC47063504ST	metagenome	Illumina HiSeq 4000	0	350	150	49317362	feces [ENVO:00002003]	0
MMPC-3109121-ST-0	MMPC17245919ST	metagenome	Illumina HiSeq 4000	0	350	150	16398595	feces [ENVO:00002003]	0
MMPC-3109123-ST-0	MMPW92389414ST	metagenome	Illumina HiSeq 4000	0	350	150	623483187	feces [ENVO:00002003]	0
MMPC-3109123-ST-0	MMPC68480658ST	metagenome	Illumina HiSeq 4000	0	350	150	13327091	feces [ENVO:00002003]	0
MMPC-3109124-ST-0	MMPC19165288ST	metagenome	Illumina HiSeq 4000	0	350	150	30229110	feces [ENVO:00002003]	0
MMPC-3109125-ST-0	MMPC57469621ST	metagenome	Illumina HiSeq 4000	0	350	150	39790710	feces [ENVO:00002003]	0
MMPC-3109126-ST-0	MMPC79996713ST	metagenome	Illumina HiSeq 4000	0	350	150	45709125	feces [ENVO:00002003]	0
MMPC-3109127-ST-0	MMPC37043483ST	metagenome	Illumina HiSeq 4000	0	350	150	7234175	feces [ENVO:00002003]	0
MMPC-3109128-ST-0	MMPC81211466ST	metagenome	Illumina HiSeq 4000	0	350	150	58854083	feces [ENVO:00002003]	0
MMPC-3109129-ST-0	MMPC24594620ST	metagenome	Illumina HiSeq 4000	0	350	150	76431268	feces [ENVO:00002003]	0
MMPC-3109130-ST-0	MMPC96182596ST	metagenome	Illumina HiSeq 4000	0	350	150	76577158	feces [ENVO:00002003]	0
MMPC-3109132-ST-0	MMPC77094060ST	metagenome	Illumina HiSeq 4000	0	350	150	36697241	feces [ENVO:00002003]	0
MMPC-3109134-ST-0	MMPC19619725ST	metagenome	Illumina HiSeq 4000	0	350	150	69280835	feces [ENVO:00002003]	0
MMPC-3109135-ST-0	MMPC16706723ST	metagenome	Illumina HiSeq 4000	0	350	150	32414314	feces [ENVO:00002003]	0
MMPC-3109136-ST-0	MMPC97506376ST	metagenome	Illumina HiSeq 4000	0	350	150	31971411	feces [ENVO:00002003]	0
MMPC-3109137-ST-0	MMPC54830782ST	metagenome	Illumina HiSeq 4000	0	350	150	38208164	feces [ENVO:00002003]	0
MMPC-3109138-ST-0	MMPC91449240ST	metagenome	Illumina HiSeq 4000	0	350	150	30254506	feces [ENVO:00002003]	0
MMPC-3109139-ST-0	MMPC55161862ST	metagenome	Illumina HiSeq 4000	0	350	150	43855433	feces [ENVO:00002003]	0
MMPC-3109140-ST-0	MMPC83312436ST	metagenome	Illumina HiSeq 4000	0	350	150	33084701	feces [ENVO:00002003]	0
MMPC-3109141-ST-0	MMPC27779234ST	metagenome	Illumina HiSeq 4000	0	350	150	44147402	feces [ENVO:00002003]	0
MMPC-3109201-ST-0	MMPC56144240ST	metagenome	Illumina HiSeq 4000	0	350	150	26869755	feces [ENVO:00002003]	0
MMPC-3109203-ST-0	MMPC13726847ST	metagenome	Illumina HiSeq 4000	0	350	150	21663895	feces [ENVO:00002003]	0
MMPC-3109204-ST-0	MMPC22312907ST	metagenome	Illumina HiSeq 4000	0	350	150	41893983	feces [ENVO:00002003]	0
MMPC-3109205-ST-0	MMPC30948868ST	metagenome	Illumina HiSeq 4000	0	350	150	21480386	feces [ENVO:00002003]	0
MMPC-3109206-ST-0	MMPC30623640ST	metagenome	Illumina HiSeq 4000	0	350	150	13694714	feces [ENVO:00002003]	0
MMPC-3109209-ST-0	MMPC97173638ST	metagenome	Illumina HiSeq 4000	0	350	150	19516074	feces [ENVO:00002003]	0
MMPC-3109210-ST-0	MMPC21135377ST	metagenome	Illumina HiSeq 4000	0	350	150	16258379	feces [ENVO:00002003]	0
MMPC-3109211-ST-0	MMPC19217531ST	metagenome	Illumina HiSeq 4000	0	350	150	53969453	feces [ENVO:00002003]	0
MMPC-3109212-ST-0	MMPC94907966ST	metagenome	Illumina HiSeq 4000	0	350	150	13649260	feces [ENVO:00002003]	0
MMPC-3109213-ST-0	MMPC60840390ST	metagenome	Illumina HiSeq 4000	0	350	150	13085767	feces [ENVO:00002003]	0
MMPC-3109215-ST-0	MMPC37485778ST	metagenome	Illumina HiSeq 4000	0	350	150	35733187	feces [ENVO:00002003]	0
MMPC-3109216-ST-0	MMPC11119852ST	metagenome	Illumina HiSeq 4000	0	350	150	14931356	feces [ENVO:00002003]	0
MMPC-3109217-ST-0	MMPC34174031ST	metagenome	Illumina HiSeq 4000	0	350	150	12765922	feces [ENVO:00002003]	0
MMPC-3109218-ST-0	MMPC52379257ST	metagenome	Illumina HiSeq 4000	0	350	150	50336511	feces [ENVO:00002003]	0
MMPC-3109219-ST-0	MMPC63364113ST	metagenome	Illumina HiSeq 4000	0	350	150	11273985	feces [ENVO:00002003]	0
MMPC-3109221-ST-0	MMPC27749512ST	metagenome	Illumina HiSeq 4000	0	350	150	22832202	feces [ENVO:00002003]	0
MMPC-3109222-ST-0	MMPC44022233ST	metagenome	Illumina HiSeq 4000	0	350	150	22174223	feces [ENVO:00002003]	0
MMPC-3109223-ST-0	MMPW83144761ST	metagenome	Illumina HiSeq 4000	0	350	150	623360223	feces [ENVO:00002003]	0
MMPC-3109223-ST-0	MMPC43926327ST	metagenome	Illumina HiSeq 4000	0	350	150	25244238	feces [ENVO:00002003]	0
MMPC-3109225-ST-0	MMPC12345974ST	metagenome	Illumina HiSeq 4000	0	350	150	85252767	feces [ENVO:00002003]	0
MMPC-3109226-ST-0	MMPC93332686ST	metagenome	Illumina HiSeq 4000	0	350	150	24901051	feces [ENVO:00002003]	0
MMPC-3109227-ST-0	MMPC92143124ST	metagenome	Illumina HiSeq 4000	0	350	150	23379659	feces [ENVO:00002003]	0
MMPC-3109228-ST-0	MMPC34385278ST	metagenome	Illumina HiSeq 4000	0	350	150	57769402	feces [ENVO:00002003]	0
MMPC-3109232-ST-0	MMPC36947286ST	metagenome	Illumina HiSeq 4000	0	350	150	31537988	feces [ENVO:00002003]	0
MMPC-3109233-ST-0	MMPC71968369ST	metagenome	Illumina HiSeq 4000	0	350	150	117568149	feces [ENVO:00002003]	0
MMPC-3109234-ST-0	MMPC74548980ST	metagenome	Illumina HiSeq 4000	0	350	150	35408139	feces [ENVO:00002003]	0
MMPC-3109236-ST-0	MMPC34834469ST	metagenome	Illumina HiSeq 4000	0	350	150	35375340	feces [ENVO:00002003]	0
MMPC-3109237-ST-0	MMPC50966165ST	metagenome	Illumina HiSeq 4000	0	350	150	52246735	feces [ENVO:00002003]	0
MMPC-3109238-ST-0	MMPC99183360ST	metagenome	Illumina HiSeq 4000	0	350	150	37883668	feces [ENVO:00002003]	0
MMPC-3109301-ST-0	MMPC35249031ST	metagenome	Illumina HiSeq 4000	0	350	150	43745786	feces [ENVO:00002003]	0
MMPC-3109305-ST-0	MMPC42290635ST	metagenome	Illumina HiSeq 4000	0	350	150	89100466	feces [ENVO:00002003]	0
MMPC-3109306-ST-0	MMPC42177786ST	metagenome	Illumina HiSeq 4000	0	350	150	22749462	feces [ENVO:00002003]	0
MMPC-3109315-ST-0	MMPC21236273ST	metagenome	Illumina HiSeq 4000	0	350	150	42079811	feces [ENVO:00002003]	0
MMPC-3109319-ST-0	MMPC11597832ST	metagenome	Illumina HiSeq 4000	0	350	150	47040985	feces [ENVO:00002003]	0
MMPC-3109324-ST-0	MMPC81505326ST	metagenome	Illumina HiSeq 4000	0	350	150	82601665	feces [ENVO:00002003]	0
MMPC-3103102-SA-0	MMPC54012223OR	metagenome	Illumina HiSeq 4000	0	350	150	4639773	saliva [ENVO:02000036]	0
MMPC-3103103-SA-0	MMPC71823580OR	metagenome	Illumina HiSeq 4000	0	350	150	10568615	saliva [ENVO:02000036]	0
MMPC-3103106-SA-0	MMPC76793553OR	metagenome	Illumina HiSeq 4000	0	350	150	5174708	saliva [ENVO:02000036]	0
MMPC-3103107-SA-0	MMPC33950978OR	metagenome	Illumina HiSeq 4000	0	350	150	3047069	saliva [ENVO:02000036]	0
MMPC-3103108-SA-0	MMPC72235353OR	metagenome	Illumina HiSeq 4000	0	350	150	24305625	saliva [ENVO:02000036]	0
MMPC-3103109-SA-0	MMPC84070936OR	metagenome	Illumina HiSeq 4000	0	350	150	22754410	saliva [ENVO:02000036]	0
MMPC-3103111-SA-0	MMPC74186942OR	metagenome	Illumina HiSeq 4000	0	350	150	13359112	saliva [ENVO:02000036]	0
MMPC-3103112-SA-0	MMPC51170653OR	metagenome	Illumina HiSeq 4000	0	350	150	9668483	saliva [ENVO:02000036]	0
MMPC-3103114-SA-0	MMPC26639644OR	metagenome	Illumina HiSeq 4000	0	350	150	26536006	saliva [ENVO:02000036]	0
MMPC-3103115-SA-0	MMPC24238465OR	metagenome	Illumina HiSeq 4000	0	350	150	1190050	saliva [ENVO:02000036]	0
MMPC-3103116-SA-0	MMPC48007504OR	metagenome	Illumina HiSeq 4000	0	350	150	27468049	saliva [ENVO:02000036]	0
MMPC-3103117-SA-0	MMPC24667695OR	metagenome	Illumina HiSeq 4000	0	350	150	5172735	saliva [ENVO:02000036]	0
MMPC-3103118-SA-0	MMPC38043539OR	metagenome	Illumina HiSeq 4000	0	350	150	20782971	saliva [ENVO:02000036]	0
MMPC-3103119-SA-0	MMPC11188431OR	metagenome	Illumina HiSeq 4000	0	350	150	25870866	saliva [ENVO:02000036]	0
MMPC-3103120-SA-0	MMPC90304226OR	metagenome	Illumina HiSeq 4000	0	350	150	19302260	saliva [ENVO:02000036]	0
MMPC-3103121-SA-0	MMPC32262645OR	metagenome	Illumina HiSeq 4000	0	350	150	19336162	saliva [ENVO:02000036]	0
MMPC-3103124-SA-0	MMPC36945431OR	metagenome	Illumina HiSeq 4000	0	350	150	2761113	saliva [ENVO:02000036]	0
MMPC-3103125-SA-0	MMPC55889507OR	metagenome	Illumina HiSeq 4000	0	350	150	10636750	saliva [ENVO:02000036]	0
MMPC-3103126-SA-0	MMPC17325940OR	metagenome	Illumina HiSeq 4000	0	350	150	9278513	saliva [ENVO:02000036]	0
MMPC-3103127-SA-0	MMPC81782599OR	metagenome	Illumina HiSeq 4000	0	350	150	1725271	saliva [ENVO:02000036]	0
MMPC-3103129-SA-0	MMPC52574889OR	metagenome	Illumina HiSeq 4000	0	350	150	4074564	saliva [ENVO:02000036]	0
MMPC-3103202-SA-0	MMPC36095950OR	metagenome	Illumina HiSeq 4000	0	350	150	2719346	saliva [ENVO:02000036]	0
MMPC-3103203-SA-0	MMPC28881424OR	metagenome	Illumina HiSeq 4000	0	350	150	22060600	saliva [ENVO:02000036]	0
MMPC-3103206-SA-0	MMPC96912492OR	metagenome	Illumina HiSeq 4000	0	350	150	29036267	saliva [ENVO:02000036]	0
MMPC-3103207-SA-0	MMPC19824023OR	metagenome	Illumina HiSeq 4000	0	350	150	24225787	saliva [ENVO:02000036]	0
MMPC-3103208-SA-0	MMPC90619750OR	metagenome	Illumina HiSeq 4000	0	350	150	3644416	saliva [ENVO:02000036]	0
MMPC-3103209-SA-0	MMPC19288609OR	metagenome	Illumina HiSeq 4000	0	350	150	5173307	saliva [ENVO:02000036]	0
MMPC-3103211-SA-0	MMPC58110896OR	metagenome	Illumina HiSeq 4000	0	350	150	14608140	saliva [ENVO:02000036]	0

MMPC-3103212-SA-0	MMPC73162206OR	metagenome	Illumina HiSeq 4000	0	350	150	13760338	saliva [ENVO:02000036]	0
MMPC-3103214-SA-0	MMPC93850203OR	metagenome	Illumina HiSeq 4000	0	350	150	23079043	saliva [ENVO:02000036]	0
MMPC-3103215-SA-0	MMPC24307334OR	metagenome	Illumina HiSeq 4000	0	350	150	24381582	saliva [ENVO:02000036]	0
MMPC-3103216-SA-0	MMPC19845294OR	metagenome	Illumina HiSeq 4000	0	350	150	586889	saliva [ENVO:02000036]	0
MMPC-3103217-SA-0	MMPC34133423OR	metagenome	Illumina HiSeq 4000	0	350	150	11088793	saliva [ENVO:02000036]	0
MMPC-3103218-SA-0	MMPC71556090OR	metagenome	Illumina HiSeq 4000	0	350	150	23124397	saliva [ENVO:02000036]	0
MMPC-3103219-SA-0	MMPC91688118OR	metagenome	Illumina HiSeq 4000	0	350	150	3228394	saliva [ENVO:02000036]	0
MMPC-3103220-SA-0	MMPC62647302OR	metagenome	Illumina HiSeq 4000	0	350	150	21615248	saliva [ENVO:02000036]	0
MMPC-3103221-SA-0	MMPC65407889OR	metagenome	Illumina HiSeq 4000	0	350	150	9669442	saliva [ENVO:02000036]	0
MMPC-3103223-SA-0	MMPC31969297OR	metagenome	Illumina HiSeq 4000	0	350	150	10392474	saliva [ENVO:02000036]	0
MMPC-3103224-SA-0	MMPC56597853OR	metagenome	Illumina HiSeq 4000	0	350	150	6055196	saliva [ENVO:02000036]	0
MMPC-3103225-SA-0	MMPC56843559OR	metagenome	Illumina HiSeq 4000	0	350	150	7623136	saliva [ENVO:02000036]	0
MMPC-3103226-SA-0	MMPC64507639OR	metagenome	Illumina HiSeq 4000	0	350	150	19496040	saliva [ENVO:02000036]	0
MMPC-3103227-SA-0	MMPC64423720OR	metagenome	Illumina HiSeq 4000	0	350	150	6255265	saliva [ENVO:02000036]	0
MMPC-3103228-SA-0	MMPC60219907OR	metagenome	Illumina HiSeq 4000	0	350	150	15063547	saliva [ENVO:02000036]	0
MMPC-3103229-SA-0	MMPC71889536OR	metagenome	Illumina HiSeq 4000	0	350	150	5309775	saliva [ENVO:02000036]	0
MMPC-3103302-SA-0	MMPC68638114OR	metagenome	Illumina HiSeq 4000	0	350	150	21593638	saliva [ENVO:02000036]	0
MMPC-3103303-SA-0	MMPC85565182OR	metagenome	Illumina HiSeq 4000	0	350	150	27146282	saliva [ENVO:02000036]	0
MMPC-3103307-SA-0	MMPC78869687OR	metagenome	Illumina HiSeq 4000	0	350	150	2896177	saliva [ENVO:02000036]	0
MMPC-3103309-SA-0	MMPC52849207OR	metagenome	Illumina HiSeq 4000	0	350	150	4140770	saliva [ENVO:02000036]	0
MMPC-3103315-SA-0	MMPC83526018OR	metagenome	Illumina HiSeq 4000	0	350	150	3405407	saliva [ENVO:02000036]	0
MMPC-3103318-SA-0	MMPC57142773OR	metagenome	Illumina HiSeq 4000	0	350	150	21599119	saliva [ENVO:02000036]	0
MMPC-3103319-SA-0	MMPC54160426OR	metagenome	Illumina HiSeq 4000	0	350	150	15712615	saliva [ENVO:02000036]	0
MMPC-3103330-SA-0	MMPC75414130OR	metagenome	Illumina HiSeq 4000	0	350	150	13142288	saliva [ENVO:02000036]	0
MMPC-3109101-SA-0	MMPC49718201OR	metagenome	Illumina HiSeq 4000	0	350	150	10271280	saliva [ENVO:02000036]	0
MMPC-3109102-SA-0	MMPC95809433OR	metagenome	Illumina HiSeq 4000	0	350	150	2270393	saliva [ENVO:02000036]	0
MMPC-3109103-SA-0	MMPC16794525OR	metagenome	Illumina HiSeq 4000	0	350	150	9038542	saliva [ENVO:02000036]	0
MMPC-3109104-SA-0	MMPC19392645OR	metagenome	Illumina HiSeq 4000	0	350	150	3568912	saliva [ENVO:02000036]	0
MMPC-3109105-SA-0	MMPC90169023OR	metagenome	Illumina HiSeq 4000	0	350	150	7204089	saliva [ENVO:02000036]	0
MMPC-3109106-SA-0	MMPC86976445OR	metagenome	Illumina HiSeq 4000	0	350	150	10768021	saliva [ENVO:02000036]	0
MMPC-3109107-SA-0	MMPC44731247OR	metagenome	Illumina HiSeq 4000	0	350	150	8048004	saliva [ENVO:02000036]	0
MMPC-3109108-SA-0	MMPC65660081OR	metagenome	Illumina HiSeq 4000	0	350	150	21617221	saliva [ENVO:02000036]	0
MMPC-3109109-SA-0	MMPC41147837OR	metagenome	Illumina HiSeq 4000	0	350	150	6344692	saliva [ENVO:02000036]	0
MMPC-3109110-SA-0	MMPC41118143OR	metagenome	Illumina HiSeq 4000	0	350	150	4560687	saliva [ENVO:02000036]	0
MMPC-3109111-SA-0	MMPC66171614OR	metagenome	Illumina HiSeq 4000	0	350	150	3102631	saliva [ENVO:02000036]	0
MMPC-3109112-SA-0	MMPC96133527OR	metagenome	Illumina HiSeq 4000	0	350	150	2593028	saliva [ENVO:02000036]	0
MMPC-3109113-SA-0	MMPC93366499OR	metagenome	Illumina HiSeq 4000	0	350	150	1364850	saliva [ENVO:02000036]	0
MMPC-3109114-SA-0	MMPC65022621OR	metagenome	Illumina HiSeq 4000	0	350	150	16802842	saliva [ENVO:02000036]	0
MMPC-3109115-SA-0	MMPC78563626OR	metagenome	Illumina HiSeq 4000	0	350	150	10555862	saliva [ENVO:02000036]	0
MMPC-3109116-SA-0	MMPC53407274OR	metagenome	Illumina HiSeq 4000	0	350	150	5335597	saliva [ENVO:02000036]	0
MMPC-3109117-SA-0	MMPC53148798OR	metagenome	Illumina HiSeq 4000	0	350	150	3146036	saliva [ENVO:02000036]	0
MMPC-3109118-SA-0	MMPC56085636OR	metagenome	Illumina HiSeq 4000	0	350	150	8008994	saliva [ENVO:02000036]	0
MMPC-3109119-SA-0	MMPC90053642OR	metagenome	Illumina HiSeq 4000	0	350	150	12510374	saliva [ENVO:02000036]	0
MMPC-3109121-SA-0	MMPC60116897OR	metagenome	Illumina HiSeq 4000	0	350	150	9130757	saliva [ENVO:02000036]	0
MMPC-3109122-SA-0	MMPC21941683OR	metagenome	Illumina HiSeq 4000	0	350	150	1074483	saliva [ENVO:02000036]	0
MMPC-3109123-SA-0	MMPC32965142OR	metagenome	Illumina HiSeq 4000	0	350	150	5930486	saliva [ENVO:02000036]	0
MMPC-3109201-SA-0	MMPC95669500OR	metagenome	Illumina HiSeq 4000	0	350	150	8322995	saliva [ENVO:02000036]	0
MMPC-3109202-SA-0	MMPC31897883OR	metagenome	Illumina HiSeq 4000	0	350	150	26509835	saliva [ENVO:02000036]	0
MMPC-3109203-SA-0	MMPC46886173OR	metagenome	Illumina HiSeq 4000	0	350	150	24073587	saliva [ENVO:02000036]	0
MMPC-3109204-SA-0	MMPC55860177OR	metagenome	Illumina HiSeq 4000	0	350	150	6325821	saliva [ENVO:02000036]	0
MMPC-3109205-SA-0	MMPC39189434OR	metagenome	Illumina HiSeq 4000	0	350	150	14245117	saliva [ENVO:02000036]	0
MMPC-3109206-SA-0	MMPC54455576OR	metagenome	Illumina HiSeq 4000	0	350	150	7426412	saliva [ENVO:02000036]	0
MMPC-3109207-SA-0	MMPC76436299OR	metagenome	Illumina HiSeq 4000	0	350	150	15891919	saliva [ENVO:02000036]	0
MMPC-3109208-SA-0	MMPC48555115OR	metagenome	Illumina HiSeq 4000	0	350	150	1067722	saliva [ENVO:02000036]	0
MMPC-3109208-SA-0	MMPC95862719OR	metagenome	Illumina HiSeq 4000	0	350	150	3806358	saliva [ENVO:02000036]	0
MMPC-3109209-SA-0	MMPC61999432OR	metagenome	Illumina HiSeq 4000	0	350	150	1080307	saliva [ENVO:02000036]	0
MMPC-3109210-SA-0	MMPC92345363OR	metagenome	Illumina HiSeq 4000	0	350	150	8191798	saliva [ENVO:02000036]	0
MMPC-3109210-SA-0	MMPC87948870OR	metagenome	Illumina HiSeq 4000	0	350	150	9477616	saliva [ENVO:02000036]	0
MMPC-3109211-SA-0	MMPC31618027OR	metagenome	Illumina HiSeq 4000	0	350	150	3968655	saliva [ENVO:02000036]	0
MMPC-3109212-SA-0	MMPC14824749OR	metagenome	Illumina HiSeq 4000	0	350	150	13866065	saliva [ENVO:02000036]	0
MMPC-3109213-SA-0	MMPC15910261OR	metagenome	Illumina HiSeq 4000	0	350	150	6374672	saliva [ENVO:02000036]	0
MMPC-3109214-SA-0	MMPC27383418OR	metagenome	Illumina HiSeq 4000	0	350	150	10627159	saliva [ENVO:02000036]	0
MMPC-3109215-SA-0	MMPC12870707OR	metagenome	Illumina HiSeq 4000	0	350	150	2480791	saliva [ENVO:02000036]	0
MMPC-3109216-SA-0	MMPC15441403OR	metagenome	Illumina HiSeq 4000	0	350	150	5975117	saliva [ENVO:02000036]	0
MMPC-3109217-SA-0	MMPC73436744OR	metagenome	Illumina HiSeq 4000	0	350	150	1084350	saliva [ENVO:02000036]	0
MMPC-3109218-SA-0	MMPC63628582OR	metagenome	Illumina HiSeq 4000	0	350	150	5800566	saliva [ENVO:02000036]	0
MMPC-3109219-SA-0	MMPC13132715OR	metagenome	Illumina HiSeq 4000	0	350	150	9675003	saliva [ENVO:02000036]	0
MMPC-3109220-SA-0	MMPC23272164OR	metagenome	Illumina HiSeq 4000	0	350	150	4438841	saliva [ENVO:02000036]	0
MMPC-3109221-SA-0	MMPC32315383OR	metagenome	Illumina HiSeq 4000	0	350	150	19454442	saliva [ENVO:02000036]	0
MMPC-3109222-SA-1	MMPC57477276OR	metagenome	Illumina HiSeq 4000	0	350	150	10220171	saliva [ENVO:02000036]	1
MMPC-3109222-SA-1	MMPC33706732OR	metagenome	Illumina HiSeq 4000	0	350	150	4735876	saliva [ENVO:02000036]	1
MMPC-3109223-SA-0	MMPC41357154OR	metagenome	Illumina HiSeq 4000	0	350	150	38849239	saliva [ENVO:02000036]	0
MMPC-3109301-SA-0	MMPC93150325OR	metagenome	Illumina HiSeq 4000	0	350	150	16728492	saliva [ENVO:02000036]	0
MMPC-3109315-SA-0	MMPC96783701OR	metagenome	Illumina HiSeq 4000	0	350	150	11039995	saliva [ENVO:02000036]	0
MMPC-3109319-SA-0	MMPC96263337OR	metagenome	Illumina HiSeq 4000	0	350	150	6969069	saliva [ENVO:02000036]	0
MMPC-3103106129-DU-0	MMPC89983827DU	16S	Illumina MiSeq	0	292	292	54547	duodenum [UBERON:0002114]	0
MMPC-3103107129-DU-0	MMPC73658283DU	16S	Illumina MiSeq	0	292	292	64250	duodenum [UBERON:0002114]	0
MMPC-3103108129-DU-0	MMPC33709560DU	16S	Illumina MiSeq	0	292	292	96936	duodenum [UBERON:0002114]	0
MMPC-3103111129-DU-0	MMPC57647850DU	16S	Illumina MiSeq	0	292	292	73326	duodenum [UBERON:0002114]	0
MMPC-3103118129-DU-0	MMPC69746296DU	16S	Illumina MiSeq	0	292	292	120850	duodenum [UBERON:0002114]	0
MMPC-3103119129-DU-0	MMPC80849866DU	16S	Illumina MiSeq	0	292	292	130442	duodenum [UBERON:0002114]	0
MMPC-3103119129-DU-c	MMPC43438216DU	16S	Illumina MiSeq	0	292	292	112252	duodenum [UBERON:0002114]	0
MMPC-3103120129-DU-0	MMPC72924201DU	16S	Illumina MiSeq	0	292	292	86565	duodenum [UBERON:0002114]	0
MMPC-3103120129-DU-c	MMPC14537843DU	16S	Illumina MiSeq	0	292	292	86185	duodenum [UBERON:0002114]	0
MMPC-3103121129-DU-0	MMPC36189783DU	16S	Illumina MiSeq	0	292	292	37643	duodenum [UBERON:0002114]	0
MMPC-3103125129-DU-0	MMPC80414181DU	16S	Illumina MiSeq	0	292	292	39130	duodenum [UBERON:0002114]	0
MMPC-3103129129-DU-0	MMPC53972105DU	16S	Illumina MiSeq	0	292	292	85409	duodenum [UBERON:0002114]	0
MMPC-3103302129-DU-0	MMPC19587595DU	16S	Illumina MiSeq	0	292	292	104839	duodenum [UBERON:0002114]	0
MMPC-3103307129-DU-0	MMPC68318389DU	16S	Illumina MiSeq	0	292	292	71624	duodenum [UBERON:0002114]	0
MMPC-3103307129-DU-0	MMPC71903222DU	16S	Illumina MiSeq	0	292	292	58134	duodenum [UBERON:0002114]	0

MMPC-3103102-ST-0	MMPC94299916ST	16S	Illumina MiSeq	0	292	292	NA	feces [ENVO:00002003]	0
MMPC-3103103-ST-0	MMPC24623808ST	16S	Illumina MiSeq	0	292	292	NA	feces [ENVO:00002003]	0
MMPC-3103106-ST-0	MMPC64014581ST	16S	Illumina MiSeq	0	292	292	NA	feces [ENVO:00002003]	0
MMPC-3103107-ST-0	MMPC82135016ST	16S	Illumina MiSeq	0	292	292	NA	feces [ENVO:00002003]	0
MMPC-3103108-ST-0	MMPC45547467ST	16S	Illumina MiSeq	0	292	292	NA	feces [ENVO:00002003]	0
MMPC-3103109-ST-0	MMPC66174878ST	16S	Illumina MiSeq	0	292	292	NA	feces [ENVO:00002003]	0
MMPC-3103111-ST-0	MMPC91082775ST	16S	Illumina MiSeq	0	292	292	NA	feces [ENVO:00002003]	0
MMPC-3103112-ST-0	MMPC29677848ST	16S	Illumina MiSeq	0	292	292	NA	feces [ENVO:00002003]	0
MMPC-3103114-ST-0	MMPC39575354ST	16S	Illumina MiSeq	0	292	292	NA	feces [ENVO:00002003]	0
MMPC-3103115-ST-0	MMPC50889135ST	16S	Illumina MiSeq	0	292	292	NA	feces [ENVO:00002003]	0
MMPC-3103116-ST-0	MMPC50185623ST	16S	Illumina MiSeq	0	292	292	NA	feces [ENVO:00002003]	0
MMPC-3103117-ST-0	MMPC85685924ST	16S	Illumina MiSeq	0	292	292	NA	feces [ENVO:00002003]	0
MMPC-3103118-ST-0	MMPC59183318ST	16S	Illumina MiSeq	0	292	292	NA	feces [ENVO:00002003]	0
MMPC-3103119-ST-0	MMPC71791865ST	16S	Illumina MiSeq	0	292	292	NA	feces [ENVO:00002003]	0
MMPC-3103120-ST-0	MMPC21711187ST	16S	Illumina MiSeq	0	292	292	NA	feces [ENVO:00002003]	0
MMPC-3103121-ST-0	MMPC21060483ST	16S	Illumina MiSeq	0	292	292	NA	feces [ENVO:00002003]	0
MMPC-3103124-ST-0	MMPC82177922ST	16S	Illumina MiSeq	0	292	292	NA	feces [ENVO:00002003]	0
MMPC-3103125-ST-0	MMPC54152785ST	16S	Illumina MiSeq	0	292	292	NA	feces [ENVO:00002003]	0
MMPC-3103126-ST-0	MMPC84720270ST	16S	Illumina MiSeq	0	292	292	NA	feces [ENVO:00002003]	0
MMPC-3103127-ST-0	MMPC61075350ST	16S	Illumina MiSeq	0	292	292	NA	feces [ENVO:00002003]	0
MMPC-3103129-ST-0	MMPC76947975ST	16S	Illumina MiSeq	0	292	292	NA	feces [ENVO:00002003]	0
MMPC-3103202-ST-0	MMPC17827720ST	16S	Illumina MiSeq	0	292	292	NA	feces [ENVO:00002003]	0
MMPC-3103203-ST-0	MMPC43730151ST	16S	Illumina MiSeq	0	292	292	NA	feces [ENVO:00002003]	0
MMPC-3103206-ST-0	MMPC62596168ST	16S	Illumina MiSeq	0	292	292	NA	feces [ENVO:00002003]	0
MMPC-3103207-ST-0	MMPC62530950ST	16S	Illumina MiSeq	0	292	292	NA	feces [ENVO:00002003]	0
MMPC-3103208-ST-0	MMPC57166568ST	16S	Illumina MiSeq	0	292	292	NA	feces [ENVO:00002003]	0
MMPC-3103209-ST-0	MMPC15725313ST	16S	Illumina MiSeq	0	292	292	NA	feces [ENVO:00002003]	0
MMPC-3103211-ST-0	MMPC84441239ST	16S	Illumina MiSeq	0	292	292	NA	feces [ENVO:00002003]	0
MMPC-3103212-ST-0	MMPC56144255ST	16S	Illumina MiSeq	0	292	292	NA	feces [ENVO:00002003]	0
MMPC-3103214-ST-0	MMPC99601038ST	16S	Illumina MiSeq	0	292	292	NA	feces [ENVO:00002003]	0
MMPC-3103215-ST-0	MMPC75618331ST	16S	Illumina MiSeq	0	292	292	NA	feces [ENVO:00002003]	0
MMPC-3103216-ST-0	MMPC16095290ST	16S	Illumina MiSeq	0	292	292	NA	feces [ENVO:00002003]	0
MMPC-3103217-ST-0	MMPC87962373ST	16S	Illumina MiSeq	0	292	292	NA	feces [ENVO:00002003]	0
MMPC-3103218-ST-0	MMPC99264450ST	16S	Illumina MiSeq	0	292	292	NA	feces [ENVO:00002003]	0
MMPC-3103219-ST-0	MMPC66473877ST	16S	Illumina MiSeq	0	292	292	NA	feces [ENVO:00002003]	0
MMPC-3103220-ST-0	MMPC80434434ST	16S	Illumina MiSeq	0	292	292	NA	feces [ENVO:00002003]	0
MMPC-3103221-ST-0	MMPC47628612ST	16S	Illumina MiSeq	0	292	292	NA	feces [ENVO:00002003]	0
MMPC-3103223-ST-0	MMPC12333772ST	16S	Illumina MiSeq	0	292	292	NA	feces [ENVO:00002003]	0
MMPC-3103224-ST-0	MMPC50116517ST	16S	Illumina MiSeq	0	292	292	NA	feces [ENVO:00002003]	0
MMPC-3103225-ST-0	MMPC44968505ST	16S	Illumina MiSeq	0	292	292	NA	feces [ENVO:00002003]	0
MMPC-3103226-ST-0	MMPC52586872ST	16S	Illumina MiSeq	0	292	292	NA	feces [ENVO:00002003]	0
MMPC-3103227-ST-0	MMPC32976245ST	16S	Illumina MiSeq	0	292	292	NA	feces [ENVO:00002003]	0
MMPC-3103228-ST-0	MMPC95231866ST	16S	Illumina MiSeq	0	292	292	NA	feces [ENVO:00002003]	0
MMPC-3103229-ST-0	MMPC68831667ST	16S	Illumina MiSeq	0	292	292	NA	feces [ENVO:00002003]	0
MMPC-3103302-ST-0	MMPC60182036ST	16S	Illumina MiSeq	0	292	292	NA	feces [ENVO:00002003]	0
MMPC-3103303-ST-0	MMPC45406530ST	16S	Illumina MiSeq	0	292	292	NA	feces [ENVO:00002003]	0
MMPC-3103306-ST-0	MMPC99577153ST	16S	Illumina MiSeq	0	292	292	NA	feces [ENVO:00002003]	0
MMPC-3103307-ST-0	MMPC93314562ST	16S	Illumina MiSeq	0	292	292	NA	feces [ENVO:00002003]	0
MMPC-3103309-ST-0	MMPC85038078ST	16S	Illumina MiSeq	0	292	292	NA	feces [ENVO:00002003]	0
MMPC-3103311-ST-0	MMPC11696984ST	16S	Illumina MiSeq	0	292	292	NA	feces [ENVO:00002003]	0
MMPC-3103312-ST-0	MMPC41061772ST	16S	Illumina MiSeq	0	292	292	NA	feces [ENVO:00002003]	0
MMPC-3103314-ST-0	MMPC45116274ST	16S	Illumina MiSeq	0	292	292	NA	feces [ENVO:00002003]	0
MMPC-3103315-ST-0	MMPC86671846ST	16S	Illumina MiSeq	0	292	292	NA	feces [ENVO:00002003]	0
MMPC-3103316-ST-0	MMPC66358697ST	16S	Illumina MiSeq	0	292	292	NA	feces [ENVO:00002003]	0
MMPC-3103317-ST-0	MMPC34674582ST	16S	Illumina MiSeq	0	292	292	NA	feces [ENVO:00002003]	0
MMPC-3103318-ST-0	MMPC22518056ST	16S	Illumina MiSeq	0	292	292	NA	feces [ENVO:00002003]	0
MMPC-3103319-ST-0	MMPC65945502ST	16S	Illumina MiSeq	0	292	292	NA	feces [ENVO:00002003]	0
MMPC-3103319-ST-0	MMPC80934998ST	16S	Illumina MiSeq	0	292	292	NA	feces [ENVO:00002003]	0
MMPC-3103321-ST-0	MMPC48683862ST	16S	Illumina MiSeq	0	292	292	NA	feces [ENVO:00002003]	0
MMPC-3103324-ST-0	MMPC83668164ST	16S	Illumina MiSeq	0	292	292	NA	feces [ENVO:00002003]	0
MMPC-3103325-ST-0	MMPC55570441ST	16S	Illumina MiSeq	0	292	292	NA	feces [ENVO:00002003]	0
MMPC-3103326-ST-0	MMPC43042571ST	16S	Illumina MiSeq	0	292	292	NA	feces [ENVO:00002003]	0
MMPC-3103329-ST-0	MMPC60345757ST	16S	Illumina MiSeq	0	292	292	NA	feces [ENVO:00002003]	0
MMPC-3103330-ST-0	MMPC29021039ST	16S	Illumina MiSeq	0	292	292	NA	feces [ENVO:00002003]	0
MMPC-3109101-ST-0	MMPC24977504ST	16S	Illumina MiSeq	0	292	292	NA	feces [ENVO:00002003]	0
MMPC-3109102-ST-0	MMPC62392110ST	16S	Illumina MiSeq	0	292	292	NA	feces [ENVO:00002003]	0
MMPC-3109103-ST-0	MMPC45287801ST	16S	Illumina MiSeq	0	292	292	NA	feces [ENVO:00002003]	0
MMPC-3109104-ST-0	MMPC40826089ST	16S	Illumina MiSeq	0	292	292	NA	feces [ENVO:00002003]	0
MMPC-3109105-ST-0	MMPC80964294ST	16S	Illumina MiSeq	0	292	292	NA	feces [ENVO:00002003]	0
MMPC-3109106-ST-0	MMPC78341196ST	16S	Illumina MiSeq	0	292	292	NA	feces [ENVO:00002003]	0
MMPC-3109108-ST-0	MMPC62146737ST	16S	Illumina MiSeq	0	292	292	NA	feces [ENVO:00002003]	0
MMPC-3109109-ST-0	MMPC69770767ST	16S	Illumina MiSeq	0	292	292	NA	feces [ENVO:00002003]	0
MMPC-3109110-ST-0	MMPC31154145ST	16S	Illumina MiSeq	0	292	292	NA	feces [ENVO:00002003]	0
MMPC-3109111-ST-0	MMPC76934936ST	16S	Illumina MiSeq	0	292	292	NA	feces [ENVO:00002003]	0
MMPC-3109112-ST-0	MMPC91853026ST	16S	Illumina MiSeq	0	292	292	NA	feces [ENVO:00002003]	0
MMPC-3109114-ST-0	MMPC31444727ST	16S	Illumina MiSeq	0	292	292	NA	feces [ENVO:00002003]	0
MMPC-3109115-ST-0	MMPC73932880ST	16S	Illumina MiSeq	0	292	292	NA	feces [ENVO:00002003]	0
MMPC-3109116-ST-0	MMPC59288570ST	16S	Illumina MiSeq	0	292	292	NA	feces [ENVO:00002003]	0
MMPC-3109117-ST-0	MMPC36926728ST	16S	Illumina MiSeq	0	292	292	NA	feces [ENVO:00002003]	0
MMPC-3109118-ST-0	MMPC96496937ST	16S	Illumina MiSeq	0	292	292	NA	feces [ENVO:00002003]	0
MMPC-3109119-ST-0	MMPC74128230ST	16S	Illumina MiSeq	0	292	292	NA	feces [ENVO:00002003]	0
MMPC-3109120-ST-0	MMPC74940396ST	16S	Illumina MiSeq	0	292	292	NA	feces [ENVO:00002003]	0
MMPC-3109121-ST-0	MMPC30256497ST	16S	Illumina MiSeq	0	292	292	NA	feces [ENVO:00002003]	0
MMPC-3109122-ST-0	MMPC21699325ST	16S	Illumina MiSeq	0	292	292	NA	feces [ENVO:00002003]	0
MMPC-3109122-ST-0	## MMPC46833664ST	16S	Illumina MiSeq	0	292	292	NA	feces [ENVO:00002003]	0
MMPC-3109123-ST-0	MMPC91722361ST	16S	Illumina MiSeq	0	292	292	NA	feces [ENVO:00002003]	0
MMPC-3109124-ST-0	MMPC94188621ST	16S	Illumina MiSeq	0	292	292	NA	feces [ENVO:00002003]	0
MMPC-3109125-ST-0	MMPC91811211ST	16S	Illumina MiSeq	0	292	292	NA	feces [ENVO:00002003]	0
MMPC-3109126-ST-0	MMPC90395905ST	16S	Illumina MiSeq	0	292	292	NA	feces [ENVO:00002003]	0
MMPC-3109136-ST-0	MMPC22960059ST	16S	Illumina MiSeq	0	292	292	NA	feces [ENVO:00002003]	0

MMPC-3109137-ST-0	MMPC61697353ST	16S	Illumina MiSeq	0	292	292	NA	feces [ENVO:00002003]	0
MMPC-3109138-ST-0	MMPC82855966ST	16S	Illumina MiSeq	0	292	292	NA	feces [ENVO:00002003]	0
MMPC-3109139-ST-0	MMPC29844214ST	16S	Illumina MiSeq	0	292	292	NA	feces [ENVO:00002003]	0
MMPC-3109140-ST-0	MMPC50483831ST	16S	Illumina MiSeq	0	292	292	NA	feces [ENVO:00002003]	0
MMPC-3109141-ST-0	MMPC48709788ST	16S	Illumina MiSeq	0	292	292	NA	feces [ENVO:00002003]	0
MMPC-3109201-ST-0	MMPC84981989ST	16S	Illumina MiSeq	0	292	292	NA	feces [ENVO:00002003]	0
MMPC-3109203-ST-0	MMPC94959046ST	16S	Illumina MiSeq	0	292	292	NA	feces [ENVO:00002003]	0
MMPC-3109204-ST-0	MMPC30552564ST	16S	Illumina MiSeq	0	292	292	NA	feces [ENVO:00002003]	0
MMPC-3109205-ST-0	MMPC80314763ST	16S	Illumina MiSeq	0	292	292	NA	feces [ENVO:00002003]	0
MMPC-3109206-ST-0	MMPC99362803ST	16S	Illumina MiSeq	0	292	292	NA	feces [ENVO:00002003]	0
MMPC-3109209-ST-0	MMPC74755332ST	16S	Illumina MiSeq	0	292	292	NA	feces [ENVO:00002003]	0
MMPC-3109210-ST-0	MMPC94006070ST	16S	Illumina MiSeq	0	292	292	NA	feces [ENVO:00002003]	0
MMPC-3109211-ST-0	MMPC61453521ST	16S	Illumina MiSeq	0	292	292	NA	feces [ENVO:00002003]	0
MMPC-3109212-ST-0	MMPC13718212ST	16S	Illumina MiSeq	0	292	292	NA	feces [ENVO:00002003]	0
MMPC-3109213-ST-0	MMPC29098777ST	16S	Illumina MiSeq	0	292	292	NA	feces [ENVO:00002003]	0
MMPC-3109215-ST-0	MMPC31684537ST	16S	Illumina MiSeq	0	292	292	NA	feces [ENVO:00002003]	0
MMPC-3109216-ST-0	MMPC52834855ST	16S	Illumina MiSeq	0	292	292	NA	feces [ENVO:00002003]	0
MMPC-3109217-ST-0	MMPC45463255ST	16S	Illumina MiSeq	0	292	292	NA	feces [ENVO:00002003]	0
MMPC-3109218-ST-0	MMPC66427657ST	16S	Illumina MiSeq	0	292	292	NA	feces [ENVO:00002003]	0
MMPC-3109219-ST-0	MMPC83101778ST	16S	Illumina MiSeq	0	292	292	NA	feces [ENVO:00002003]	0
MMPC-3109221-ST-0	MMPC45481781ST	16S	Illumina MiSeq	0	292	292	NA	feces [ENVO:00002003]	0
MMPC-3109222-ST-0	MMPC28617437ST	16S	Illumina MiSeq	0	292	292	NA	feces [ENVO:00002003]	0
MMPC-3109223-ST-0	MMPC49582267ST	16S	Illumina MiSeq	0	292	292	NA	feces [ENVO:00002003]	0
MMPC-3109226-ST-0	MMPC57772499ST	16S	Illumina MiSeq	0	292	292	NA	feces [ENVO:00002003]	0
MMPC-3109232-ST-0	MMPC92950288ST	16S	Illumina MiSeq	0	292	292	NA	feces [ENVO:00002003]	0
MMPC-3109234-ST-0	MMPC35779891ST	16S	Illumina MiSeq	0	292	292	NA	feces [ENVO:00002003]	0
MMPC-3109236-ST-0	MMPC19331607ST	16S	Illumina MiSeq	0	292	292	NA	feces [ENVO:00002003]	0
MMPC-3109237-ST-0	MMPC55213614ST	16S	Illumina MiSeq	0	292	292	NA	feces [ENVO:00002003]	0
MMPC-3109238-ST-0	MMPC25677890ST	16S	Illumina MiSeq	0	292	292	NA	feces [ENVO:00002003]	0
MMPC-3109301-ST-0	MMPC43182434ST	16S	Illumina MiSeq	0	292	292	NA	feces [ENVO:00002003]	0
MMPC-3109315-ST-0	MMPC38663896ST	16S	Illumina MiSeq	0	292	292	NA	feces [ENVO:00002003]	0
MMPC-3109319-ST-0	MMPC66201737ST	16S	Illumina MiSeq	0	292	292	NA	feces [ENVO:00002003]	0
MMPC-3103102-SA-0	MMPC68158800OR	16S	Illumina MiSeq	0	292	292	NA	saliva [ENVO:02000036]	0
MMPC-3103103-SA-0	MMPC55436260OR	16S	Illumina MiSeq	0	292	292	NA	saliva [ENVO:02000036]	0
MMPC-3103106-SA-0	MMPC88921841OR	16S	Illumina MiSeq	0	292	292	NA	saliva [ENVO:02000036]	0
MMPC-3103107-SA-0	MMPC71486420OR	16S	Illumina MiSeq	0	292	292	NA	saliva [ENVO:02000036]	0
MMPC-3103108-SA-0	MMPC46739872OR	16S	Illumina MiSeq	0	292	292	NA	saliva [ENVO:02000036]	0
MMPC-3103109-SA-0	MMPC31622432OR	16S	Illumina MiSeq	0	292	292	NA	saliva [ENVO:02000036]	0
MMPC-3103111-SA-0	MMPC51692793OR	16S	Illumina MiSeq	0	292	292	NA	saliva [ENVO:02000036]	0
MMPC-3103112-SA-0	MMPC12922693OR	16S	Illumina MiSeq	0	292	292	NA	saliva [ENVO:02000036]	0
MMPC-3103114-SA-0	MMPC22137064OR	16S	Illumina MiSeq	0	292	292	NA	saliva [ENVO:02000036]	0
MMPC-3103115-SA-0	MMPC67978674OR	16S	Illumina MiSeq	0	292	292	NA	saliva [ENVO:02000036]	0
MMPC-3103116-SA-0	MMPC65303959OR	16S	Illumina MiSeq	0	292	292	NA	saliva [ENVO:02000036]	0
MMPC-3103117-SA-0	MMPC63118595OR	16S	Illumina MiSeq	0	292	292	NA	saliva [ENVO:02000036]	0
MMPC-3103118-SA-0	MMPC11179595OR	16S	Illumina MiSeq	0	292	292	NA	saliva [ENVO:02000036]	0
MMPC-3103119-SA-0	MMPC20954519OR	16S	Illumina MiSeq	0	292	292	NA	saliva [ENVO:02000036]	0
MMPC-3103120-SA-0	MMPC21775404OR	16S	Illumina MiSeq	0	292	292	NA	saliva [ENVO:02000036]	0
MMPC-3103121-SA-0	MMPC23514621OR	16S	Illumina MiSeq	0	292	292	NA	saliva [ENVO:02000036]	0
MMPC-3103124-SA-0	MMPC42192127OR	16S	Illumina MiSeq	0	292	292	NA	saliva [ENVO:02000036]	0
MMPC-3103125-SA-0	MMPC45339999OR	16S	Illumina MiSeq	0	292	292	NA	saliva [ENVO:02000036]	0
MMPC-3103126-SA-0	MMPC68048817OR	16S	Illumina MiSeq	0	292	292	NA	saliva [ENVO:02000036]	0
MMPC-3103127-SA-0	MMPC62601303OR	16S	Illumina MiSeq	0	292	292	NA	saliva [ENVO:02000036]	0
MMPC-3103129-SA-0	MMPC31367206OR	16S	Illumina MiSeq	0	292	292	NA	saliva [ENVO:02000036]	0
MMPC-3103202-SA-0	MMPC42991028OR	16S	Illumina MiSeq	0	292	292	NA	saliva [ENVO:02000036]	0
MMPC-3103203-SA-0	MMPC74059481OR	16S	Illumina MiSeq	0	292	292	NA	saliva [ENVO:02000036]	0
MMPC-3103206-SA-0	MMPC74490281OR	16S	Illumina MiSeq	0	292	292	NA	saliva [ENVO:02000036]	0
MMPC-3103207-SA-0	MMPC53231545OR	16S	Illumina MiSeq	0	292	292	NA	saliva [ENVO:02000036]	0
MMPC-3103208-SA-0	MMPC48136943OR	16S	Illumina MiSeq	0	292	292	NA	saliva [ENVO:02000036]	0
MMPC-3103209-SA-0	MMPC20195220OR	16S	Illumina MiSeq	0	292	292	NA	saliva [ENVO:02000036]	0
MMPC-3103211-SA-0	MMPC45318061OR	16S	Illumina MiSeq	0	292	292	NA	saliva [ENVO:02000036]	0
MMPC-3103212-SA-0	MMPC37399343OR	16S	Illumina MiSeq	0	292	292	NA	saliva [ENVO:02000036]	0
MMPC-3103214-SA-0	MMPC38394840OR	16S	Illumina MiSeq	0	292	292	NA	saliva [ENVO:02000036]	0
MMPC-3103215-SA-0	MMPC84970403OR	16S	Illumina MiSeq	0	292	292	NA	saliva [ENVO:02000036]	0
MMPC-3103216-SA-0	MMPC56379457OR	16S	Illumina MiSeq	0	292	292	NA	saliva [ENVO:02000036]	0
MMPC-3103217-SA-0	MMPC40967934OR	16S	Illumina MiSeq	0	292	292	NA	saliva [ENVO:02000036]	0
MMPC-3103218-SA-0	MMPC73927114OR	16S	Illumina MiSeq	0	292	292	NA	saliva [ENVO:02000036]	0
MMPC-3103219-SA-0	MMPC97872292OR	16S	Illumina MiSeq	0	292	292	NA	saliva [ENVO:02000036]	0
MMPC-3103220-SA-0	MMPC68758730OR	16S	Illumina MiSeq	0	292	292	NA	saliva [ENVO:02000036]	0
MMPC-3103221-SA-0	MMPC54458294OR	16S	Illumina MiSeq	0	292	292	NA	saliva [ENVO:02000036]	0
MMPC-3103223-SA-0	MMPC55525481OR	16S	Illumina MiSeq	0	292	292	NA	saliva [ENVO:02000036]	0
MMPC-3103224-SA-0	MMPC90470189OR	16S	Illumina MiSeq	0	292	292	NA	saliva [ENVO:02000036]	0
MMPC-3103225-SA-0	MMPC85390604OR	16S	Illumina MiSeq	0	292	292	NA	saliva [ENVO:02000036]	0
MMPC-3103226-SA-0	MMPC87516576OR	16S	Illumina MiSeq	0	292	292	NA	saliva [ENVO:02000036]	0
MMPC-3103227-SA-0	MMPC50847145OR	16S	Illumina MiSeq	0	292	292	NA	saliva [ENVO:02000036]	0
MMPC-3103228-SA-0	MMPC24608714OR	16S	Illumina MiSeq	0	292	292	NA	saliva [ENVO:02000036]	0
MMPC-3103229-SA-0	MMPC58829082OR	16S	Illumina MiSeq	0	292	292	NA	saliva [ENVO:02000036]	0
MMPC-3103302-SA-0	MMPC63553475OR	16S	Illumina MiSeq	0	292	292	NA	saliva [ENVO:02000036]	0
MMPC-3103303-SA-0	MMPC27266285OR	16S	Illumina MiSeq	0	292	292	NA	saliva [ENVO:02000036]	0
MMPC-3103306-SA-0	MMPC85705244OR	16S	Illumina MiSeq	0	292	292	NA	saliva [ENVO:02000036]	0
MMPC-3103307-SA-0	MMPC57892266OR	16S	Illumina MiSeq	0	292	292	NA	saliva [ENVO:02000036]	0
MMPC-3103308-SA-0	MMPC58558599OR	16S	Illumina MiSeq	0	292	292	NA	saliva [ENVO:02000036]	0
MMPC-3103309-SA-0	MMPC55133623OR	16S	Illumina MiSeq	0	292	292	NA	saliva [ENVO:02000036]	0
MMPC-3103311-SA-0	MMPC37416843OR	16S	Illumina MiSeq	0	292	292	NA	saliva [ENVO:02000036]	0
MMPC-3103312-SA-0	MMPC50998073OR	16S	Illumina MiSeq	0	292	292	NA	saliva [ENVO:02000036]	0
MMPC-3103314-SA-0	MMPC35036620OR	16S	Illumina MiSeq	0	292	292	NA	saliva [ENVO:02000036]	0
MMPC-3103315-SA-0	MMPC79959139OR	16S	Illumina MiSeq	0	292	292	NA	saliva [ENVO:02000036]	0
MMPC-3103316-SA-0	MMPC65969751OR	16S	Illumina MiSeq	0	292	292	NA	saliva [ENVO:02000036]	0
MMPC-3103317-SA-0	MMPC85634415OR	16S	Illumina MiSeq	0	292	292	NA	saliva [ENVO:02000036]	0
MMPC-3103318-SA-0	MMPC93112149OR	16S	Illumina MiSeq	0	292	292	NA	saliva [ENVO:02000036]	0
MMPC-3103319-SA-0	MMPC12461225OR	16S	Illumina MiSeq	0	292	292	NA	saliva [ENVO:02000036]	0

MMPC-3103320-SA-0	MMPC34498596OR	16S	Illumina MiSeq	0	292	292	NA	saliva [ENVO:02000036]	0
MMPC-3103321-SA-0	MMPC56325888OR	16S	Illumina MiSeq	0	292	292	NA	saliva [ENVO:02000036]	0
MMPC-3103324-SA-0	MMPC99338054OR	16S	Illumina MiSeq	0	292	292	NA	saliva [ENVO:02000036]	0
MMPC-3103325-SA-0	MMPC96167252OR	16S	Illumina MiSeq	0	292	292	NA	saliva [ENVO:02000036]	0
MMPC-3103326-SA-0	MMPC55035531OR	16S	Illumina MiSeq	0	292	292	NA	saliva [ENVO:02000036]	0
MMPC-3103327-SA-0	MMPC81837161OR	16S	Illumina MiSeq	0	292	292	NA	saliva [ENVO:02000036]	0
MMPC-3103329-SA-0	MMPC42646817OR	16S	Illumina MiSeq	0	292	292	NA	saliva [ENVO:02000036]	0
MMPC-3103330-SA-0	MMPC16231550OR	16S	Illumina MiSeq	0	292	292	NA	saliva [ENVO:02000036]	0
MMPC-3109101-SA-0	MMPC91896521OR	16S	Illumina MiSeq	0	292	292	NA	saliva [ENVO:02000036]	0
MMPC-3109102-SA-0	MMPC91354261OR	16S	Illumina MiSeq	0	292	292	NA	saliva [ENVO:02000036]	0
MMPC-3109103-SA-0	MMPC49489785OR	16S	Illumina MiSeq	0	292	292	NA	saliva [ENVO:02000036]	0
MMPC-3109104-SA-0	MMPC78236242OR	16S	Illumina MiSeq	0	292	292	NA	saliva [ENVO:02000036]	0
MMPC-3109105-SA-0	MMPC46123748OR	16S	Illumina MiSeq	0	292	292	NA	saliva [ENVO:02000036]	0
MMPC-3109106-SA-0	MMPC95372332OR	16S	Illumina MiSeq	0	292	292	NA	saliva [ENVO:02000036]	0
MMPC-3109107-SA-0	MMPC42247540OR	16S	Illumina MiSeq	0	292	292	NA	saliva [ENVO:02000036]	0
MMPC-3109108-SA-0	MMPC22594900OR	16S	Illumina MiSeq	0	292	292	NA	saliva [ENVO:02000036]	0
MMPC-3109109-SA-0	MMPC42369256OR	16S	Illumina MiSeq	0	292	292	NA	saliva [ENVO:02000036]	0
MMPC-3109110-SA-0	MMPC52559088OR	16S	Illumina MiSeq	0	292	292	NA	saliva [ENVO:02000036]	0
MMPC-3109111-SA-0	MMPC48186797OR	16S	Illumina MiSeq	0	292	292	NA	saliva [ENVO:02000036]	0
MMPC-3109112-SA-0	MMPC35162108OR	16S	Illumina MiSeq	0	292	292	NA	saliva [ENVO:02000036]	0
MMPC-3109113-SA-0	MMPC77190173OR	16S	Illumina MiSeq	0	292	292	NA	saliva [ENVO:02000036]	0
MMPC-3109114-SA-0	MMPC91664155OR	16S	Illumina MiSeq	0	292	292	NA	saliva [ENVO:02000036]	0
MMPC-3109115-SA-0	MMPC86145124OR	16S	Illumina MiSeq	0	292	292	NA	saliva [ENVO:02000036]	0
MMPC-3109116-SA-0	MMPC28815814OR	16S	Illumina MiSeq	0	292	292	NA	saliva [ENVO:02000036]	0
MMPC-3109117-SA-0	MMPC55730016OR	16S	Illumina MiSeq	0	292	292	NA	saliva [ENVO:02000036]	0
MMPC-3109118-SA-0	MMPC73361074OR	16S	Illumina MiSeq	0	292	292	NA	saliva [ENVO:02000036]	0
MMPC-3109119-SA-0	MMPC99082893OR	16S	Illumina MiSeq	0	292	292	NA	saliva [ENVO:02000036]	0
MMPC-3109120-SA-0	MMPC34568026OR	16S	Illumina MiSeq	0	292	292	NA	saliva [ENVO:02000036]	0
MMPC-3109121-SA-0	MMPC91033830OR	16S	Illumina MiSeq	0	292	292	NA	saliva [ENVO:02000036]	0
MMPC-3109122-SA-0	MMPC75634766OR	16S	Illumina MiSeq	0	292	292	NA	saliva [ENVO:02000036]	0
MMPC-3109123-SA-0	MMPC78765773OR	16S	Illumina MiSeq	0	292	292	NA	saliva [ENVO:02000036]	0
MMPC-3109124-SA-0	MMPC42978442OR	16S	Illumina MiSeq	0	292	292	NA	saliva [ENVO:02000036]	0
MMPC-3109125-SA-0	MMPC13169976OR	16S	Illumina MiSeq	0	292	292	NA	saliva [ENVO:02000036]	0
MMPC-3109126-SA-0	MMPC21250627OR	16S	Illumina MiSeq	0	292	292	NA	saliva [ENVO:02000036]	0
MMPC-3109127-SA-0	MMPC89348908OR	16S	Illumina MiSeq	0	292	292	NA	saliva [ENVO:02000036]	0
MMPC-3109128-SA-0	MMPC41437470OR	16S	Illumina MiSeq	0	292	292	NA	saliva [ENVO:02000036]	0
MMPC-3109129-SA-0	MMPC68946435OR	16S	Illumina MiSeq	0	292	292	NA	saliva [ENVO:02000036]	0
MMPC-3109130-SA-0	MMPC10578010OR	16S	Illumina MiSeq	0	292	292	NA	saliva [ENVO:02000036]	0
MMPC-3109132-SA-0	MMPC58837755OR	16S	Illumina MiSeq	0	292	292	NA	saliva [ENVO:02000036]	0
MMPC-3109134-SA-0	MMPC42441792OR	16S	Illumina MiSeq	0	292	292	NA	saliva [ENVO:02000036]	0
MMPC-3109135-SA-0	MMPC88095540OR	16S	Illumina MiSeq	0	292	292	NA	saliva [ENVO:02000036]	0
MMPC-3109136-SA-0	MMPC67321556OR	16S	Illumina MiSeq	0	292	292	NA	saliva [ENVO:02000036]	0
MMPC-3109137-SA-0	MMPC25056624OR	16S	Illumina MiSeq	0	292	292	NA	saliva [ENVO:02000036]	0
MMPC-3109138-SA-0	MMPC29668362OR	16S	Illumina MiSeq	0	292	292	NA	saliva [ENVO:02000036]	0
MMPC-3109139-SA-0	MMPC80846477OR	16S	Illumina MiSeq	0	292	292	NA	saliva [ENVO:02000036]	0
MMPC-3109140-SA-0	MMPC92702195OR	16S	Illumina MiSeq	0	292	292	NA	saliva [ENVO:02000036]	0
MMPC-3109141-SA-0	MMPC42107863OR	16S	Illumina MiSeq	0	292	292	NA	saliva [ENVO:02000036]	0
MMPC-3109201-SA-0	MMPC85802458OR	16S	Illumina MiSeq	0	292	292	NA	saliva [ENVO:02000036]	0
MMPC-3109202-SA-0	MMPC27037046OR	16S	Illumina MiSeq	0	292	292	NA	saliva [ENVO:02000036]	0
MMPC-3109203-SA-0	MMPC42326903OR	16S	Illumina MiSeq	0	292	292	NA	saliva [ENVO:02000036]	0
MMPC-3109204-SA-0	MMPC96578111OR	16S	Illumina MiSeq	0	292	292	NA	saliva [ENVO:02000036]	0
MMPC-3109205-SA-0	MMPC67377108OR	16S	Illumina MiSeq	0	292	292	NA	saliva [ENVO:02000036]	0
MMPC-3109206-SA-0	MMPC24821675OR	16S	Illumina MiSeq	0	292	292	NA	saliva [ENVO:02000036]	0
MMPC-3109207-SA-0	MMPC60572150OR	16S	Illumina MiSeq	0	292	292	NA	saliva [ENVO:02000036]	0
MMPC-3109208-SA-0	MMPC27025376OR	16S	Illumina MiSeq	0	292	292	NA	saliva [ENVO:02000036]	0
MMPC-3109208-SA-0	MMPC62405084OR	16S	Illumina MiSeq	0	292	292	NA	saliva [ENVO:02000036]	0
MMPC-3109209-SA-0	MMPC19177783OR	16S	Illumina MiSeq	0	292	292	NA	saliva [ENVO:02000036]	0
MMPC-3109210-SA-0	MMPC24845793OR	16S	Illumina MiSeq	0	292	292	NA	saliva [ENVO:02000036]	0
MMPC-3109210-SA-0	MMPC47372291OR	16S	Illumina MiSeq	0	292	292	NA	saliva [ENVO:02000036]	0
MMPC-3109211-SA-0	MMPC89522066OR	16S	Illumina MiSeq	0	292	292	NA	saliva [ENVO:02000036]	0
MMPC-3109212-SA-0	MMPC73773857OR	16S	Illumina MiSeq	0	292	292	NA	saliva [ENVO:02000036]	0
MMPC-3109213-SA-0	MMPC37783467OR	16S	Illumina MiSeq	0	292	292	NA	saliva [ENVO:02000036]	0
MMPC-3109214-SA-0	MMPC39636267OR	16S	Illumina MiSeq	0	292	292	NA	saliva [ENVO:02000036]	0
MMPC-3109215-SA-0	MMPC40536708OR	16S	Illumina MiSeq	0	292	292	NA	saliva [ENVO:02000036]	0
MMPC-3109216-SA-0	MMPC43503506OR	16S	Illumina MiSeq	0	292	292	NA	saliva [ENVO:02000036]	0
MMPC-3109217-SA-0	MMPC22439119OR	16S	Illumina MiSeq	0	292	292	NA	saliva [ENVO:02000036]	0
MMPC-3109218-SA-0	MMPC93571858OR	16S	Illumina MiSeq	0	292	292	NA	saliva [ENVO:02000036]	0
MMPC-3109219-SA-0	MMPC45008410OR	16S	Illumina MiSeq	0	292	292	NA	saliva [ENVO:02000036]	0
MMPC-3109220-SA-0	MMPC29277640OR	16S	Illumina MiSeq	0	292	292	NA	saliva [ENVO:02000036]	0
MMPC-3109221-SA-0	MMPC32291597OR	16S	Illumina MiSeq	0	292	292	NA	saliva [ENVO:02000036]	0
MMPC-3109222-SA-1	MMPC99658636OR	16S	Illumina MiSeq	0	292	292	NA	saliva [ENVO:02000036]	1
MMPC-3109222-SA-1	MMPC13427371OR	16S	Illumina MiSeq	0	292	292	NA	saliva [ENVO:02000036]	1
MMPC-3109223-SA-0	MMPC48873249OR	16S	Illumina MiSeq	0	292	292	NA	saliva [ENVO:02000036]	0
MMPC-3109225-SA-0	MMPC37580697OR	16S	Illumina MiSeq	0	292	292	NA	saliva [ENVO:02000036]	0
MMPC-3109226-SA-0	MMPC61992937OR	16S	Illumina MiSeq	0	292	292	NA	saliva [ENVO:02000036]	0
MMPC-3109227-SA-0	MMPC67918047OR	16S	Illumina MiSeq	0	292	292	NA	saliva [ENVO:02000036]	0
MMPC-3109228-SA-0	MMPC35478733OR	16S	Illumina MiSeq	0	292	292	NA	saliva [ENVO:02000036]	0
MMPC-3109232-SA-0	MMPC54640065OR	16S	Illumina MiSeq	0	292	292	NA	saliva [ENVO:02000036]	0
MMPC-3109233-SA-0	MMPC94081974OR	16S	Illumina MiSeq	0	292	292	NA	saliva [ENVO:02000036]	0
MMPC-3109234-SA-0	MMPC29658279OR	16S	Illumina MiSeq	0	292	292	NA	saliva [ENVO:02000036]	0
MMPC-3109236-SA-0	MMPC40797909OR	16S	Illumina MiSeq	0	292	292	NA	saliva [ENVO:02000036]	0
MMPC-3109237-SA-0	MMPC47432795OR	16S	Illumina MiSeq	0	292	292	NA	saliva [ENVO:02000036]	0
MMPC-3109238-SA-0	MMPC81538982OR	16S	Illumina MiSeq	0	292	292	NA	saliva [ENVO:02000036]	0
MMPC-3109301-SA-0	MMPC62882996OR	16S	Illumina MiSeq	0	292	292	NA	saliva [ENVO:02000036]	0
MMPC-3109305-SA-0	MMPC59454227OR	16S	Illumina MiSeq	0	292	292	NA	saliva [ENVO:02000036]	0
MMPC-3109305-SA-0	MMPC63021713OR	16S	Illumina MiSeq	0	292	292	NA	saliva [ENVO:02000036]	0
MMPC-3109306-SA-0	MMPC33663665OR	16S	Illumina MiSeq	0	292	292	NA	saliva [ENVO:02000036]	0
MMPC-3109315-SA-0	MMPC59175809OR	16S	Illumina MiSeq	0	292	292	NA	saliva [ENVO:02000036]	0
MMPC-3109319-SA-0	MMPC52031211OR	16S	Illumina MiSeq	0	292	292	NA	saliva [ENVO:02000036]	0
MMPC-3109324-SA-0	MMPC11599193OR	16S	Illumina MiSeq	0	292	292	NA	saliva [ENVO:02000036]	0

MMPC-3103102123-TU-0	MMPC52294238TU	16S	Illumina MiSeq	0	292	292	16762	pancreas [UBERON:0001264]	0
MMPC-3103102125-HT-0	MMPC96437096HT	16S	Illumina MiSeq	0	292	292	118983	pancreas [UBERON:0001264]	0
MMPC-3103106123-TU-0	MMPC22271503TU	16S	Illumina MiSeq	0	292	292	27346	pancreas [UBERON:0001264]	0
MMPC-3103107123-TU-0	MMPC87955180TU	16S	Illumina MiSeq	0	292	292	50932	pancreas [UBERON:0001264]	0
MMPC-3103108123-TU-0	MMPC54922715TU	16S	Illumina MiSeq	0	292	292	31245	pancreas [UBERON:0001264]	0
MMPC-3103108125-HT-0	MMPC24466339HT	16S	Illumina MiSeq	0	292	292	127835	pancreas [UBERON:0001264]	0
MMPC-3103111125-HT-0	MMPC80734101HT	16S	Illumina MiSeq	0	292	292	95924	pancreas [UBERON:0001264]	0
MMPC-3103112123-TU-0	MMPC83770412TU	16S	Illumina MiSeq	0	292	292	21764	pancreas [UBERON:0001264]	0
MMPC-3103112125-HT-0	MMPC40334378HT	16S	Illumina MiSeq	0	292	292	91218	pancreas [UBERON:0001264]	0
MMPC-3103114123-TU-0	MMPC48644619TU	16S	Illumina MiSeq	0	292	292	17849	pancreas [UBERON:0001264]	0
MMPC-3103114125-HT-0	MMPC35209818HT	16S	Illumina MiSeq	0	292	292	114214	pancreas [UBERON:0001264]	0
MMPC-3103116123-TU-0	MMPC75019385TU	16S	Illumina MiSeq	0	292	292	38264	pancreas [UBERON:0001264]	0
MMPC-3103116125-HT-0	MMPC89754577HT	16S	Illumina MiSeq	0	292	292	84488	pancreas [UBERON:0001264]	0
MMPC-3103116125-HT-c	MMPC76929964HT	16S	Illumina MiSeq	0	292	292	99306	pancreas [UBERON:0001264]	0
MMPC-3103118123-TU-0	MMPC53213093TU	16S	Illumina MiSeq	0	292	292	19403	pancreas [UBERON:0001264]	0
MMPC-3103119123-TU-0	MMPC77465766TU	16S	Illumina MiSeq	0	292	292	35083	pancreas [UBERON:0001264]	0
MMPC-3103119123-TU-c	MMPC24078588TU	16S	Illumina MiSeq	0	292	292	71331	pancreas [UBERON:0001264]	0
MMPC-3103119125-HT-0	MMPC37714156HT	16S	Illumina MiSeq	0	292	292	85195	pancreas [UBERON:0001264]	0
MMPC-3103119125-HT-c	MMPC49102349HT	16S	Illumina MiSeq	0	292	292	111138	pancreas [UBERON:0001264]	0
MMPC-3103120125-HT-0	MMPC36983914HT	16S	Illumina MiSeq	0	292	292	73461	pancreas [UBERON:0001264]	0
MMPC-3103121123-TU-0	MMPC90323932TU	16S	Illumina MiSeq	0	292	292	32825	pancreas [UBERON:0001264]	0
MMPC-3103121123-TU-c	MMPC58485046TU	16S	Illumina MiSeq	0	292	292	140836	pancreas [UBERON:0001264]	0
MMPC-3103125123-TU-0	MMPC49164313TU	16S	Illumina MiSeq	0	292	292	50097	pancreas [UBERON:0001264]	0
MMPC-3103125127-HT-0	MMPC86673187HT	16S	Illumina MiSeq	0	292	292	96102	pancreas [UBERON:0001264]	0
MMPC-3103129123-TU-0	MMPC80997236TU	16S	Illumina MiSeq	0	292	292	64881	pancreas [UBERON:0001264]	0
MMPC-3103129125-HT-0	MMPC49203482HT	16S	Illumina MiSeq	0	292	292	110366	pancreas [UBERON:0001264]	0
MMPC-3103302125-HT-0	MMPC25083626HT	16S	Illumina MiSeq	0	292	292	87920	pancreas [UBERON:0001264]	0
MMPC-3103302127-HT-0	MMPC02321546HT	16S	Illumina MiSeq	0	292	292	94815	ncreatic duct [UBERON:00073	0
MMPC-3103307123-TU-0	MMPC84306689TU	16S	Illumina MiSeq	0	292	292	35125	pancreas [UBERON:0001264]	0
MMPC-3103307123-TU-c	MMPC63721423TU	16S	Illumina MiSeq	0	292	292	101573	pancreas [UBERON:0001264]	0
MMPC-3103307125-HT-0	MMPC74146850HT	16S	Illumina MiSeq	0	292	292	87273	pancreas [UBERON:0001264]	0
MMPC-3103309123-TU-0	MMPC53390762TU	16S	Illumina MiSeq	0	292	292	25942	pancreas [UBERON:0001264]	0
MMPC-3103309125-HT-0	MMPC33872709HT	16S	Illumina MiSeq	0	292	292	93994	pancreas [UBERON:0001264]	0
MMPC-3103330125-HT-0	MMPC35081045HT	16S	Illumina MiSeq	0	292	292	75059	pancreas [UBERON:0001264]	0
MMPC-3109101123-TU-0	MMPC82234530TU	16S	Illumina MiSeq	0	292	292	63990	pancreas [UBERON:0001264]	0
MMPC-3109102123-TU-0	MMPC26308927TU	16S	Illumina MiSeq	0	292	292	63118	pancreas [UBERON:0001264]	0
MMPC-3109102125-HT-0	MMPC83100372HT	16S	Illumina MiSeq	0	292	292	97384	pancreas [UBERON:0001264]	0
MMPC-3109103123-TU-0	MMPC79418921TU	16S	Illumina MiSeq	0	292	292	92173	pancreas [UBERON:0001264]	0
MMPC-3109105123-TU-0	MMPC96387591TU	16S	Illumina MiSeq	0	292	292	86922	pancreas [UBERON:0001264]	0
MMPC-3109105125-HT-0	MMPC33126922HT	16S	Illumina MiSeq	0	292	292	96057	pancreas [UBERON:0001264]	0
MMPC-3109107123-TU-0	MMPC80047993TU	16S	Illumina MiSeq	0	292	292	78692	pancreas [UBERON:0001264]	0
MMPC-3109107125-HT-0	MMPC65170291HT	16S	Illumina MiSeq	0	292	292	66822	pancreas [UBERON:0001264]	0
MMPC-3109111123-TU-0	MMPC23067667TU	16S	Illumina MiSeq	0	292	292	8	pancreas [UBERON:0001264]	0
MMPC-3109111125-HT-0	MMPC80978814HT	16S	Illumina MiSeq	0	292	292	150064	pancreas [UBERON:0001264]	0
MMPC-3109111125-HT-c	MMPC42159796HT	16S	Illumina MiSeq	0	292	292	116464	pancreas [UBERON:0001264]	0
MMPC-3109113123-TU-0	MMPC42025082TU	16S	Illumina MiSeq	0	292	292	91603	pancreas [UBERON:0001264]	0
MMPC-3109113125-HT-0	MMPC93630826HT	16S	Illumina MiSeq	0	292	292	93581	pancreas [UBERON:0001264]	0
MMPC-3109117123-TU-0	MMPC44512561TU	16S	Illumina MiSeq	0	292	292	101695	pancreas [UBERON:0001264]	0
MMPC-3109117125-HT-0	MMPC58544120HT	16S	Illumina MiSeq	0	292	292	76970	pancreas [UBERON:0001264]	0
MMPC-3109117127-HT-0	MMPC66546739HT	16S	Illumina MiSeq	0	292	292	72952	ncreatic duct [UBERON:00073	0
MMPC-3109140123-TU-0	MMPC57986041TU	16S	Illumina MiSeq	0	292	292	137586	pancreas [UBERON:0001264]	0

gender	age_years	ollection_dat	ct_disease_s	tax_id	smoker	center	alcohol_statu	alldiab	diabcat	obese	panctype	asthma	allacid
male	79	2015-2018	PC	408170	smoker	2	0	0	0	0	0	0	0
male	62	2015-2018	PC	408170	ex-smoker	2	1	0	0	0	0	0	0
female	69	2015-2018	PC	408170	smoker	2	0	0	0	0	0	0	1
male	54	2015-2018	PC	408170	non-smoker	2	1	0	0	0	0	0	0
male	54	2015-2018	PC	408170	non-smoker	2	1	0	0	0	0	0	0
female	68	2015-2018	PC	408170	non-smoker	2	1	0	0	NA	0	0	0
male	68	2015-2018	PC	408170	ex-smoker	2	1	1	1	0	0	0	0
male	68	2015-2018	PC	408170	ex-smoker	2	1	1	1	0	0	0	0
male	71	2015-2018	PC	408170	non-smoker	2	0	0	0	0	0	0	0
male	84	2015-2018	PC	408170	non-smoker	2	1	1	1	0	0	0	0
male	84	2015-2018	PC	408170	non-smoker	2	1	1	1	0	0	0	0
female	69	2015-2018	PC	408170	non-smoker	2	0	0	0	0	0	1	1
male	66	2015-2018	PC	408170	smoker	2	1	0	0	0	0	0	1
male	82	2015-2018	PC	408170	ex-smoker	2	1	1	2	0	0	0	0
male	82	2015-2018	PC	408170	ex-smoker	2	1	1	2	0	0	0	0
male	70	2015-2018	PC	408170	non-smoker	2	1	1	2	1	0	0	0
male	70	2015-2018	PC	408170	non-smoker	2	1	1	2	1	0	0	0
male	60	2015-2018	PC	408170	smoker	2	1	0	0	0	2	0	0
male	63	2015-2018	PC	408170	ex-smoker	2	1	0	0	1	0	0	0
male	45	2015-2018	PC	408170	ex-smoker	2	1	0	0	0	NA	1	0
female	73	2015-2018	PC	408170	non-smoker	2	1	0	0	1	0	0	0
female	67	2015-2018	PC	408170	non-smoker	2	0	0	0	0	0	0	0
female	67	2015-2018	PC	408170	non-smoker	2	0	0	0	0	0	0	0
male	66	2015-2018	PC	408170	non-smoker	2	0	1	2	0	0	0	0
male	68	2015-2018	PC	408170	smoker	2	1	1	1	0	0	0	0
male	52	2015-2018	PC	408170	smoker	2	1	0	0	1	0	0	1
male	68	2015-2018	PC	408170	non-smoker	2	1	0	0	0	0	0	0
male	83	2015-2018	CTR	408170	ex-smoker	2	1	0	0	NA	0	0	0
male	57	2015-2018	CTR	408170	non-smoker	2	1	0	0	0	0	0	0
male	57	2015-2018	CTR	408170	non-smoker	2	1	0	0	0	0	0	0
female	70	2015-2018	CTR	408170	non-smoker	2	0	0	0	0	1	0	1
male	54	2015-2018	CTR	408170	non-smoker	2	1	0	0	0	0	0	0
female	72	2015-2018	CTR	408170	smoker	2	1	0	0	0	0	0	0
male	76	2015-2018	Pancreatitis	408170	ex-smoker	2	1	1	2	1	2	0	0
male	67	2015-2018	CTR	408170	non-smoker	2	1	1	2	0	0	0	0
male	67	2015-2018	CTR	408170	non-smoker	2	1	1	2	0	0	0	0
male	82	2015-2018	CTR	408170	non-smoker	2	0	0	0	0	0	0	0
female	64	2015-2018	CTR	408170	non-smoker	2	1	0	0	0	0	0	0
male	63	2015-2018	CTR	408170	ex-smoker	2	1	0	0	0	0	0	1
male	84	2015-2018	CTR	408170	ex-smoker	2	1	1	2	1	0	0	0
male	61	2015-2018	CTR	408170	smoker	2	1	0	0	0	0	0	0
male	53	2015-2018	CTR	408170	ex-smoker	2	1	0	0	0	0	0	0
male	57	2015-2018	CTR	408170	non-smoker	2	1	0	0	1	0	0	1
male	38	2015-2018	CTR	408170	ex-smoker	2	1	0	0	0	0	0	0
female	68	2015-2018	CTR	408170	ex-smoker	2	1	0	0	0	0	0	0
female	81	2015-2018	CTR	408170	non-smoker	2	1	0	0	0	0	0	1
female	65	2015-2018	CTR	408170	non-smoker	2	1	0	0	0	0	0	0
male	61	2015-2018	CTR	408170	non-smoker	2	0	0	0	0	0	0	0
male	59	2015-2018	CTR	408170	ex-smoker	2	1	0	0	1	0	0	0
male	56	2015-2018	CTR	408170	smoker	2	1	0	0	0	0	0	0
male	63	2015-2018	CTR	408170	smoker	2	1	0	0	0	0	0	1
male	63	2015-2018	CTR	408170	smoker	2	1	0	0	0	0	0	0
male	79	2015-2018	Pancreatitis	408170	ex-smoker	2	1	1	2	0	2	0	0
male	64	2015-2018	Pancreatitis	408170	smoker	2	1	1	2	0	2	0	0
female	62	2015-2018	Pancreatitis	408170	ex-smoker	2	1	1	2	1	2	1	0
male	54	2015-2018	Pancreatitis	408170	smoker	2	1	0	0	0	2	0	0
female	58	2015-2018	Pancreatitis	408170	smoker	2	1	1	1	0	2	0	1
male	58	2015-2018	Pancreatitis	408170	smoker	2	1	0	0	0	2	0	0
male	67	2015-2018	Pancreatitis	408170	smoker	2	1	0	0	0	2	0	0
male	76	2015-2018	Pancreatitis	408170	ex-smoker	2	1	1	2	0	2	0	0
female	72	2015-2018	Pancreatitis	408170	ex-smoker	2	1	1	2	0	2	0	0
male	58	2015-2018	Pancreatitis	408170	smoker	2	1	1	2	1	2	0	0
male	78	2015-2018	Pancreatitis	408170	ex-smoker	2	1	1	2	0	2	0	0
male	60	2015-2018	Pancreatitis	408170	ex-smoker	2	1	1	2	0	2	0	1
male	58	2015-2018	Pancreatitis	408170	smoker	2	1	1	2	0	2	0	0
male	64	2015-2018	Pancreatitis	408170	ex-smoker	2	1	1	1	1	2	0	1
male	64	2015-2018	Pancreatitis	408170	ex-smoker	2	1	1	1	1	2	0	1
male	45	2015-2018	Pancreatitis	408170	ex-smoker	2	1	0	0	0	2	0	1
female	83	2015-2018	Pancreatitis	408170	smoker	2	0	1	2	0	2	0	0
female	71	2015-2018	Pancreatitis	408170	non-smoker	2	0	0	0	0	2	1	0
male	64	2015-2018	Pancreatitis	408170	smoker	2	1	1	2	0	2	0	0
male	61	2015-2018	Pancreatitis	408170	smoker	2	1	1	2	0	2	0	0
male	55	2015-2018	Pancreatitis	408170	smoker	2	1	1	2	0	2	0	0
male	62	2015-2018	Pancreatitis	408170	smoker	2	1	1	2	0	2	0	0
female	55	2015-2018	Pancreatitis	408170	smoker	2	1	0	0	1	2	0	0
female	78	2015-2018	PC	408170	non-smoker	8	0	0	0	0	0	0	0
female	78	2015-2018	PC	408170	non-smoker	8	0	0	0	0	0	0	0
female	67	2015-2018	PC	408170	smoker	8	0	0	0	0	0	1	1
female	74	2015-2018	PC	408170	non-smoker	8	0	0	0	0	0	0	0
female	74	2015-2018	PC	408170	non-smoker	8	0	0	0	0	0	0	0
male	85	2015-2018	PC	408170	ex-smoker	8	0	0	0	0	0	0	0
female	71	2015-2018	PC	408170	non-smoker	8	0	1	1	1	0	0	1
female	68	2015-2018	PC	408170	non-smoker	8	1	1	1	0	0	0	0
female	81	2015-2018	PC	408170	non-smoker	8	1	1	1	1	0	0	0
male	86	2015-2018	PC	408170	ex-smoker	8	1	0	0	0	0	0	0
female	71	2015-2018	PC	408170	non-smoker	8	1	0	0	0	0	0	1
male	85	2015-2018	PC	408170	ex-smoker	8	1	1	2	0	0	0	0
male	85	2015-2018	PC	408170	ex-smoker	8	1	0	0	0	0	0	1

female	84	2015-2018	PC	408170	non-smoker	8	1	1	1	1	0	0	1
male	74	2015-2018	PC	408170	non-smoker	8	1	0	0	1	0	0	1
male	88	2015-2018	PC	408170	ex-smoker	8	1	0	0	0	0	0	1
female	79	2015-2018	PC	408170	non-smoker	8	0	0	0	0	0	0	1
female	79	2015-2018	PC	408170	non-smoker	8	0	0	0	0	0	0	1
male	80	2015-2018	PC	408170	non-smoker	8	1	1	2	0	0	0	1
male	49	2015-2018	PC	408170	non-smoker	8	0	0	0	0	0	1	1
male	79	2015-2018	PC	408170	smoker	8	1	0	0	0	0	0	0
female	80	2015-2018	PC	408170	non-smoker	8	0	0	0	0	0	0	0
male	86	2015-2018	PC	408170	NA	8	1	0	0	1	0	0	0
male	86	2015-2018	PC	408170	NA	8	1	0	0	1	0	0	0
male	70	2015-2018	PC	408170	ex-smoker	8	1	0	0	0	0	0	0
female	85	2015-2018	PC	408170	non-smoker	8	1	0	0	1	0	0	0
female	71	2015-2018	PC	408170	non-smoker	8	1	0	0	0	0	0	0
male	67	2015-2018	PC	408170	ex-smoker	8	1	0	0	0	0	0	0
male	69	2015-2018	PC	408170	ex-smoker	8	1	1	2	1	0	0	0
female	75	2015-2018	PC	408170	non-smoker	8	0	0	0	0	0	0	0
female	79	2015-2018	PC	408170	non-smoker	8	0	0	0	1	0	0	0
female	74	2015-2018	PC	408170	non-smoker	8	0	0	0	0	0	0	0
male	62	2015-2018	PC	408170	ex-smoker	8	1	1	2	0	1	0	1
male	83	2015-2018	PC	408170	ex-smoker	8	1	1	2	0	0	0	0
female	65	2015-2018	PC	408170	ex-smoker	8	1	0	0	0	0	0	0
male	56	2015-2018	PC	408170	ex-smoker	8	1	0	0	1	0	0	1
male	78	2015-2018	PC	408170	ex-smoker	8	1	0	0	1	0	0	0
male	45	2015-2018	PC	408170	smoker	8	1	0	0	1	0	0	1
male	81	2015-2018	PC	408170	non-smoker	8	1	1	2	1	0	0	0
male	64	2015-2018	PC	408170	smoker	8	1	1	1	0	0	0	0
female	88	2015-2018	CTR	408170	non-smoker	8	1	0	0	NA	0	0	0
female	80	2015-2018	CTR	408170	non-smoker	8	0	1	2	1	0	1	1
male	88	2015-2018	CTR	408170	ex-smoker	8	1	0	0	0	0	0	0
female	79	2015-2018	CTR	408170	non-smoker	8	1	0	0	1	0	0	0
female	59	2015-2018	CTR	408170	non-smoker	8	0	0	0	1	0	0	0
male	91	2015-2018	CTR	408170	non-smoker	8	1	0	0	0	0	0	1
female	62	2015-2018	CTR	408170	non-smoker	8	1	0	0	0	0	0	0
male	81	2015-2018	CTR	408170	ex-smoker	8	1	1	2	0	0	0	0
male	81	2015-2018	CTR	408170	ex-smoker	8	1	0	0	0	0	0	1
female	76	2015-2018	CTR	408170	non-smoker	8	0	0	0	0	0	0	0
male	71	2015-2018	CTR	408170	non-smoker	8	1	0	0	1	0	0	0
male	93	2015-2018	CTR	408170	smoker	8	1	0	0	1	0	1	0
female	85	2015-2018	CTR	408170	smoker	8	0	0	0	0	0	0	0
male	72	2015-2018	CTR	408170	smoker	8	1	0	0	1	0	0	0
male	49	2015-2018	CTR	408170	non-smoker	8	1	0	0	0	0	0	1
female	87	2015-2018	CTR	408170	non-smoker	8	0	1	2	1	0	0	0
male	87	2015-2018	CTR	408170	ex-smoker	8	1	0	0	0	0	0	0
male	78	2015-2018	CTR	408170	smoker	8	1	0	0	1	0	0	0
male	78	2015-2018	CTR	408170	smoker	8	1	0	0	1	0	0	0
female	88	2015-2018	CTR	408170	non-smoker	8	0	0	0	0	0	0	0
female	69	2015-2018	CTR	408170	ex-smoker	8	1	0	0	0	0	0	0
male	73	2015-2018	CTR	408170	ex-smoker	8	1	0	0	0	0	0	0
male	77	2015-2018	CTR	408170	non-smoker	8	1	0	0	0	0	0	0
female	85	2015-2018	CTR	408170	non-smoker	8	0	0	0	0	0	0	0
female	89	2015-2018	CTR	408170	non-smoker	8	1	NA	NA	0	0	1	0
male	69	2015-2018	CTR	408170	ex-smoker	8	1	0	0	1	0	0	1
female	58	2015-2018	CTR	408170	ex-smoker	8	1	0	0	0	0	0	0
male	59	2015-2018	CTR	408170	ex-smoker	8	0	0	0	0	0	0	0
male	71	2015-2018	CTR	408170	smoker	8	1	0	0	1	0	1	0
female	77	2015-2018	Pancreatitis	408170	non-smoker	8	1	0	0	0	2	0	1
female	51	2015-2018	Pancreatitis	408170	ex-smoker	8	1	0	0	0	2	0	1
female	56	2015-2018	Pancreatitis	408170	non-smoker	8	1	0	0	0	2	0	1
male	74	2015-2018	Pancreatitis	408170	ex-smoker	8	1	0	0	1	2	0	0
male	54	2015-2018	Pancreatitis	408170	non-smoker	8	1	1	2	0	2	0	0
male	68	2015-2018	Pancreatitis	408170	smoker	8	0	1	2	1	2	0	0
male	79	2015-2018	PC	1679718	smoker	2	0	0	0	0	0	0	0
male	62	2015-2018	PC	1679718	ex-smoker	2	1	0	0	0	0	0	0
female	69	2015-2018	PC	1679718	smoker	2	0	0	0	0	0	0	1
male	54	2015-2018	PC	1679718	non-smoker	2	1	0	0	0	0	0	0
female	68	2015-2018	PC	1679718	non-smoker	2	1	0	0	NA	0	0	0
male	68	2015-2018	PC	1679718	ex-smoker	2	1	1	1	0	0	0	0
male	71	2015-2018	PC	1679718	non-smoker	2	0	0	0	0	0	0	0
male	84	2015-2018	PC	1679718	non-smoker	2	1	1	1	0	0	0	0
female	69	2015-2018	PC	1679718	non-smoker	2	0	0	0	0	0	1	1
male	66	2015-2018	PC	1679718	smoker	2	1	0	0	0	0	0	1
male	82	2015-2018	PC	1679718	ex-smoker	2	1	1	2	0	0	0	0
male	70	2015-2018	PC	1679718	non-smoker	2	1	1	2	1	0	0	0
male	60	2015-2018	PC	1679718	smoker	2	1	0	0	0	2	0	0
male	63	2015-2018	PC	1679718	ex-smoker	2	1	0	0	1	0	0	0
male	45	2015-2018	PC	1679718	ex-smoker	2	1	0	0	0	NA	1	0
female	73	2015-2018	PC	1679718	non-smoker	2	1	0	0	1	0	0	0
female	67	2015-2018	PC	1679718	non-smoker	2	0	0	0	0	0	0	0
male	66	2015-2018	PC	1679718	non-smoker	2	0	1	2	0	0	0	0
male	68	2015-2018	PC	1679718	smoker	2	1	1	1	0	0	0	0
male	52	2015-2018	PC	1679718	smoker	2	1	0	0	1	0	0	1
male	68	2015-2018	PC	1679718	non-smoker	2	1	0	0	0	0	0	0
male	83	2015-2018	CTR	1679718	ex-smoker	2	1	0	0	NA	0	0	0
male	57	2015-2018	CTR	1679718	non-smoker	2	1	0	0	0	0	0	0
female	70	2015-2018	CTR	1679718	non-smoker	2	0	0	0	0	1	0	1
male	54	2015-2018	CTR	1679718	non-smoker	2	1	0	0	0	0	0	0
female	72	2015-2018	CTR	1679718	smoker	2	1	0	0	0	0	0	0
male	76	2015-2018	Pancreatitis	1679718	ex-smoker	2	1	1	2	1	2	0	0
male	67	2015-2018	CTR	1679718	non-smoker	2	1	1	2	0	0	0	0

male	82	2015-2018	CTR	1679718	non-smoker	2	0	0	0	0	0	0	0
female	64	2015-2018	CTR	1679718	non-smoker	2	1	0	0	0	0	0	0
male	63	2015-2018	CTR	1679718	ex-smoker	2	1	0	0	0	0	0	1
male	84	2015-2018	CTR	1679718	ex-smoker	2	1	1	2	1	0	0	0
male	61	2015-2018	CTR	1679718	smoker	2	1	0	0	0	0	0	0
male	53	2015-2018	CTR	1679718	ex-smoker	2	1	0	0	0	0	0	0
male	57	2015-2018	CTR	1679718	non-smoker	2	1	0	0	1	0	0	1
male	38	2015-2018	CTR	1679718	ex-smoker	2	1	0	0	0	0	0	0
female	68	2015-2018	CTR	1679718	ex-smoker	2	1	0	0	0	0	0	0
female	81	2015-2018	CTR	1679718	non-smoker	2	1	0	0	0	0	0	1
female	65	2015-2018	CTR	1679718	non-smoker	2	1	0	0	0	0	0	0
male	61	2015-2018	CTR	1679718	non-smoker	2	0	0	0	0	0	0	0
male	59	2015-2018	CTR	1679718	ex-smoker	2	1	0	0	1	0	0	0
male	56	2015-2018	CTR	1679718	smoker	2	1	0	0	0	0	0	0
male	63	2015-2018	CTR	1679718	smoker	2	1	0	0	0	0	0	1
male	63	2015-2018	CTR	1679718	smoker	2	1	0	0	0	0	0	0
male	79	2015-2018	Pancreatitis	1679718	ex-smoker	2	1	1	2	0	2	0	0
male	64	2015-2018	Pancreatitis	1679718	smoker	2	1	1	2	0	2	0	0
male	54	2015-2018	Pancreatitis	1679718	smoker	2	1	0	0	0	2	0	0
male	58	2015-2018	Pancreatitis	1679718	smoker	2	1	0	0	0	2	0	0
male	58	2015-2018	Pancreatitis	1679718	smoker	2	1	1	2	1	2	0	0
male	58	2015-2018	Pancreatitis	1679718	smoker	2	1	1	2	0	2	0	0
male	64	2015-2018	Pancreatitis	1679718	ex-smoker	2	1	1	1	1	2	0	1
female	55	2015-2018	Pancreatitis	1679718	smoker	2	1	0	0	1	2	0	0
female	78	2015-2018	PC	1679718	non-smoker	8	0	0	0	0	0	0	0
female	67	2015-2018	PC	1679718	smoker	8	0	0	0	0	0	1	1
female	74	2015-2018	PC	1679718	non-smoker	8	0	0	0	0	0	0	0
male	85	2015-2018	PC	1679718	ex-smoker	8	0	0	0	0	0	0	0
female	71	2015-2018	PC	1679718	non-smoker	8	0	1	1	1	0	0	1
female	68	2015-2018	PC	1679718	non-smoker	8	1	1	1	0	0	0	0
female	81	2015-2018	PC	1679718	ex-smoker	8	0	1	2	1	0	0	0
female	81	2015-2018	PC	1679718	non-smoker	8	1	1	1	1	0	0	0
male	86	2015-2018	PC	1679718	ex-smoker	8	1	0	0	0	0	0	0
female	71	2015-2018	PC	1679718	non-smoker	8	1	0	0	0	0	0	1
male	85	2015-2018	PC	1679718	ex-smoker	8	1	1	2	0	0	0	0
male	85	2015-2018	PC	1679718	ex-smoker	8	1	0	0	0	0	0	1
female	78	2015-2018	PC	1679718	non-smoker	8	0	0	0	1	0	0	0
female	84	2015-2018	PC	1679718	non-smoker	8	1	1	1	1	0	0	1
male	74	2015-2018	PC	1679718	non-smoker	8	1	0	0	1	0	0	1
male	88	2015-2018	PC	1679718	ex-smoker	8	1	0	0	0	0	0	1
female	79	2015-2018	PC	1679718	non-smoker	8	0	0	0	0	0	0	1
male	80	2015-2018	PC	1679718	non-smoker	8	1	1	2	0	0	0	1
male	49	2015-2018	PC	1679718	non-smoker	8	0	0	0	0	0	1	1
female	80	2015-2018	PC	1679718	non-smoker	8	0	0	0	0	0	0	0
male	81	2015-2018	PC	1679718	non-smoker	8	0	0	0	0	0	0	0
male	86	2015-2018	PC	1679718	NA	8	1	0	0	1	0	0	0
female	88	2015-2018	CTR	1679718	non-smoker	8	1	0	0	NA	0	0	0
female	68	2015-2018	CTR	1679718	ex-smoker	8	1	0	0	0	0	0	0
female	80	2015-2018	CTR	1679718	non-smoker	8	0	1	2	1	0	1	1
male	88	2015-2018	CTR	1679718	ex-smoker	8	1	0	0	0	0	0	0
female	79	2015-2018	CTR	1679718	non-smoker	8	1	0	0	1	0	0	0
female	59	2015-2018	CTR	1679718	non-smoker	8	0	0	0	1	0	0	0
female	77	2015-2018	CTR	1679718	non-smoker	8	1	0	0	1	0	0	0
female	84	2015-2018	CTR	1679718	non-smoker	8	0	0	0	0	0	0	0
female	84	2015-2018	CTR	1679718	non-smoker	8	0	0	0	0	0	0	0
male	91	2015-2018	CTR	1679718	non-smoker	8	1	0	0	0	0	0	1
female	62	2015-2018	CTR	1679718	non-smoker	8	1	0	0	0	0	0	0
female	62	2015-2018	CTR	1679718	non-smoker	8	1	0	0	0	0	0	0
male	81	2015-2018	CTR	1679718	ex-smoker	8	1	1	2	0	0	0	0
male	81	2015-2018	CTR	1679718	ex-smoker	8	1	0	0	0	0	0	1
female	76	2015-2018	CTR	1679718	non-smoker	8	0	0	0	0	0	0	0
female	78	2015-2018	CTR	1679718	non-smoker	8	0	1	2	0	0	0	1
male	71	2015-2018	CTR	1679718	non-smoker	8	1	0	0	1	0	0	0
male	93	2015-2018	CTR	1679718	smoker	8	1	0	0	1	0	1	0
female	85	2015-2018	CTR	1679718	smoker	8	0	0	0	0	0	0	0
male	72	2015-2018	CTR	1679718	smoker	8	1	0	0	1	0	0	0
male	49	2015-2018	CTR	1679718	non-smoker	8	1	0	0	0	0	0	1
female	78	2015-2018	CTR	1679718	smoker	8	1	NA	0	0	0	0	0
female	87	2015-2018	CTR	1679718	non-smoker	8	0	1	2	1	0	0	0
male	87	2015-2018	CTR	1679718	ex-smoker	8	1	0	0	0	0	0	0
male	87	2015-2018	CTR	1679718	ex-smoker	8	1	0	0	0	0	0	0
male	78	2015-2018	CTR	1679718	smoker	8	1	0	0	1	0	0	0
female	77	2015-2018	Pancreatitis	1679718	non-smoker	8	1	0	0	0	2	0	1
male	74	2015-2018	Pancreatitis	1679718	ex-smoker	8	1	0	0	1	2	0	0
male	54	2015-2018	Pancreatitis	1679718	non-smoker	8	1	1	2	0	2	0	0
female	68	2015-2017	PC	646099	smoker	2	0	0	0	0	0	0	1
male	54	2015-2017	PC	646099	non-smoker	2	1	0	0	0	0	0	0
female	68	2015-2017	PC	646099	non-smoker	2	1	0	0	1	0	0	0
male	71	2015-2017	PC	646099	non-smoker	2	0	0	0	0	0	0	0
male	60	2015-2017	PC	646099	smoker	2	1	0	0	0	2	0	0
male	63	2015-2017	PC	646099	ex-smoker	2	1	0	0	1	0	0	0
male	63	2015-2017	PC	646099	ex-smoker	2	1	0	0	1	0	0	0
male	45	2015-2017	PC	646099	ex-smoker	2	1	0	0	0	0	1	0
male	45	2015-2017	PC	646099	ex-smoker	2	1	0	0	0	0	1	0
female	73	2015-2017	PC	646099	non-smoker	2	1	0	0	1	0	0	0
male	66	2015-2017	PC	646099	non-smoker	2	0	1	2	0	0	0	0
male	68	2015-2017	PC	646099	non-smoker	2	1	0	0	0	0	0	0
male	79	2015-2017	Pancreatitis	646099	ex-smoker	2	1	1	2	0	2	0	0
male	54	2015-2017	Pancreatitis	646099	NA	2	1	0	0	0	1	0	0
male	54	2015-2017	Pancreatitis	646099	NA	2	1	0	0	0	1	0	0

male	79	2015-2018	PC	408170	smoker	2	0	0	0	0	0	0	0
male	62	2015-2018	PC	408170	ex-smoker	2	1	0	0	0	0	0	0
female	69	2015-2018	PC	408170	smoker	2	0	0	0	0	0	0	1
male	54	2015-2018	PC	408170	non-smoker	2	1	0	0	0	0	0	0
female	68	2015-2018	PC	408170	non-smoker	2	1	0	0	NA	0	0	0
male	68	2015-2018	PC	408170	ex-smoker	2	1	1	1	0	0	0	0
male	71	2015-2018	PC	408170	non-smoker	2	0	0	0	0	0	0	0
male	84	2015-2018	PC	408170	non-smoker	2	1	1	1	0	0	0	0
female	69	2015-2018	PC	408170	non-smoker	2	0	0	0	0	0	1	1
male	66	2015-2018	PC	408170	smoker	2	1	0	0	0	0	0	1
male	82	2015-2018	PC	408170	ex-smoker	2	1	1	2	0	0	0	0
male	70	2015-2018	PC	408170	non-smoker	2	1	1	2	1	0	0	0
male	60	2015-2018	PC	408170	smoker	2	1	0	0	0	2	0	0
male	63	2015-2018	PC	408170	ex-smoker	2	1	0	0	1	0	0	0
male	45	2015-2018	PC	408170	ex-smoker	2	1	0	0	0	NA	1	0
female	73	2015-2018	PC	408170	non-smoker	2	1	0	0	1	0	0	0
female	67	2015-2018	PC	408170	non-smoker	2	0	0	0	0	0	0	0
male	66	2015-2018	PC	408170	non-smoker	2	0	1	2	0	0	0	0
male	68	2015-2018	PC	408170	smoker	2	1	1	1	0	0	0	0
male	52	2015-2018	PC	408170	smoker	2	1	0	0	1	0	0	1
male	68	2015-2018	PC	408170	non-smoker	2	1	0	0	0	0	0	0
male	83	2015-2018	CTR	408170	ex-smoker	2	1	0	0	NA	0	0	0
male	57	2015-2018	CTR	408170	non-smoker	2	1	0	0	0	0	0	0
female	70	2015-2018	CTR	408170	non-smoker	2	0	0	0	0	1	0	1
male	54	2015-2018	CTR	408170	non-smoker	2	1	0	0	0	0	0	0
female	72	2015-2018	CTR	408170	smoker	2	1	0	0	0	0	0	0
male	76	2015-2018	Pancreatitis	408170	ex-smoker	2	1	1	2	1	2	0	0
male	67	2015-2018	CTR	408170	non-smoker	2	1	1	2	0	0	0	0
male	82	2015-2018	CTR	408170	non-smoker	2	0	0	0	0	0	0	0
female	64	2015-2018	CTR	408170	non-smoker	2	1	0	0	0	0	0	0
male	63	2015-2018	CTR	408170	ex-smoker	2	1	0	0	0	0	0	1
male	84	2015-2018	CTR	408170	ex-smoker	2	1	1	2	1	0	0	0
male	61	2015-2018	CTR	408170	smoker	2	1	0	0	0	0	0	0
male	53	2015-2018	CTR	408170	ex-smoker	2	1	0	0	0	0	0	0
male	57	2015-2018	CTR	408170	non-smoker	2	1	0	0	1	0	0	1
male	38	2015-2018	CTR	408170	ex-smoker	2	1	0	0	0	0	0	0
female	68	2015-2018	CTR	408170	ex-smoker	2	1	0	0	0	0	0	0
female	81	2015-2018	CTR	408170	non-smoker	2	1	0	0	0	0	0	1
female	65	2015-2018	CTR	408170	non-smoker	2	1	0	0	0	0	0	0
male	61	2015-2018	CTR	408170	non-smoker	2	0	0	0	0	0	0	0
male	59	2015-2018	CTR	408170	ex-smoker	2	1	0	0	1	0	0	0
male	56	2015-2018	CTR	408170	smoker	2	1	0	0	0	0	0	0
male	63	2015-2018	CTR	408170	smoker	2	1	0	0	0	0	0	1
male	63	2015-2018	CTR	408170	smoker	2	1	0	0	0	0	0	0
male	79	2015-2018	Pancreatitis	408170	ex-smoker	2	1	1	2	0	2	0	0
male	64	2015-2018	Pancreatitis	408170	smoker	2	1	1	2	0	2	0	0
female	62	2015-2018	Pancreatitis	408170	ex-smoker	2	1	1	2	1	2	1	0
male	54	2015-2018	Pancreatitis	408170	smoker	2	1	0	0	0	2	0	0
male	58	2015-2018	Pancreatitis	408170	smoker	2	1	0	0	0	2	0	0
male	67	2015-2018	Pancreatitis	408170	smoker	2	1	0	0	0	2	0	0
male	76	2015-2018	Pancreatitis	408170	ex-smoker	2	1	1	2	0	2	0	0
female	72	2015-2018	Pancreatitis	408170	ex-smoker	2	1	1	2	0	2	0	0
male	58	2015-2018	Pancreatitis	408170	smoker	2	1	1	2	1	2	0	0
male	78	2015-2018	Pancreatitis	408170	ex-smoker	2	1	1	2	0	2	0	0
male	60	2015-2018	Pancreatitis	408170	ex-smoker	2	1	1	2	0	2	0	1
male	58	2015-2018	Pancreatitis	408170	smoker	2	1	1	2	0	2	0	0
male	64	2015-2018	Pancreatitis	408170	ex-smoker	2	1	1	1	1	2	0	1
male	64	2015-2018	Pancreatitis	408170	ex-smoker	2	1	1	1	1	2	0	1
female	83	2015-2018	Pancreatitis	408170	smoker	2	0	1	2	0	2	0	0
female	71	2015-2018	Pancreatitis	408170	non-smoker	2	0	0	0	0	2	1	0
male	64	2015-2018	Pancreatitis	408170	smoker	2	1	1	2	0	2	0	0
male	61	2015-2018	Pancreatitis	408170	smoker	2	1	1	2	0	2	0	0
male	62	2015-2018	Pancreatitis	408170	smoker	2	1	1	2	0	2	0	0
female	55	2015-2018	Pancreatitis	408170	smoker	2	1	0	0	1	2	0	0
female	78	2015-2018	PC	408170	non-smoker	8	0	0	0	0	0	0	0
female	67	2015-2018	PC	408170	smoker	8	0	0	0	0	0	1	1
female	74	2015-2018	PC	408170	non-smoker	8	0	0	0	0	0	0	0
male	85	2015-2018	PC	408170	ex-smoker	8	0	0	0	0	0	0	0
female	71	2015-2018	PC	408170	non-smoker	8	0	1	1	1	0	0	1
female	68	2015-2018	PC	408170	non-smoker	8	1	1	1	0	0	0	0
female	81	2015-2018	PC	408170	non-smoker	8	1	1	1	1	0	0	0
male	86	2015-2018	PC	408170	ex-smoker	8	1	0	0	0	0	0	0
female	71	2015-2018	PC	408170	non-smoker	8	1	0	0	0	0	0	1
male	85	2015-2018	PC	408170	ex-smoker	8	1	1	2	0	0	0	0
male	85	2015-2018	PC	408170	ex-smoker	8	1	0	0	0	0	0	1
female	84	2015-2018	PC	408170	non-smoker	8	1	1	1	1	0	0	1
male	74	2015-2018	PC	408170	non-smoker	8	1	0	0	1	0	0	1
male	88	2015-2018	PC	408170	ex-smoker	8	1	0	0	0	0	0	1
female	79	2015-2018	PC	408170	non-smoker	8	0	0	0	0	0	0	1
male	80	2015-2018	PC	408170	non-smoker	8	1	1	2	0	0	0	1
male	49	2015-2018	PC	408170	non-smoker	8	0	0	0	0	0	1	1
male	79	2015-2018	PC	408170	smoker	8	1	0	0	0	0	0	0
female	80	2015-2018	PC	408170	non-smoker	8	0	0	0	0	0	0	0
male	81	2015-2018	PC	408170	non-smoker	8	0	0	0	0	0	0	0
male	81	2015-2018	PC	408170	non-smoker	8	0	0	0	0	0	0	0
male	86	2015-2018	PC	408170	NA	8	1	0	0	1	0	0	0
male	70	2015-2018	PC	408170	ex-smoker	8	1	0	0	0	0	0	0
female	85	2015-2018	PC	408170	non-smoker	8	1	0	0	1	0	0	0
female	71	2015-2018	PC	408170	non-smoker	8	1	0	0	0	0	0	0
female	65	2015-2018	PC	408170	ex-smoker	8	1	0	0	0	0	0	0

male	56	2015-2018	PC	408170	ex-smoker	8	1	0	0	1	0	0	1
male	78	2015-2018	PC	408170	ex-smoker	8	1	0	0	1	0	0	0
male	45	2015-2018	PC	408170	smoker	8	1	0	0	1	0	0	1
male	81	2015-2018	PC	408170	non-smoker	8	1	1	2	1	0	0	0
male	64	2015-2018	PC	408170	smoker	8	1	1	1	0	0	0	0
female	88	2015-2018	CTR	408170	non-smoker	8	1	0	0	NA	0	0	0
female	80	2015-2018	CTR	408170	non-smoker	8	0	1	2	1	0	1	1
male	88	2015-2018	CTR	408170	ex-smoker	8	1	0	0	0	0	0	0
female	79	2015-2018	CTR	408170	non-smoker	8	1	0	0	1	0	0	0
female	59	2015-2018	CTR	408170	non-smoker	8	0	0	0	1	0	0	0
male	91	2015-2018	CTR	408170	non-smoker	8	1	0	0	0	0	0	1
female	62	2015-2018	CTR	408170	non-smoker	8	1	0	0	0	0	0	0
male	81	2015-2018	CTR	408170	ex-smoker	8	1	1	2	0	0	0	0
male	81	2015-2018	CTR	408170	ex-smoker	8	1	0	0	0	0	0	1
female	76	2015-2018	CTR	408170	non-smoker	8	0	0	0	0	0	0	0
male	71	2015-2018	CTR	408170	non-smoker	8	1	0	0	1	0	0	0
male	93	2015-2018	CTR	408170	smoker	8	1	0	0	1	0	1	0
female	85	2015-2018	CTR	408170	smoker	8	0	0	0	0	0	0	0
male	72	2015-2018	CTR	408170	smoker	8	1	0	0	1	0	0	0
male	49	2015-2018	CTR	408170	non-smoker	8	1	0	0	0	0	0	1
female	87	2015-2018	CTR	408170	non-smoker	8	0	1	2	1	0	0	0
male	87	2015-2018	CTR	408170	ex-smoker	8	1	0	0	0	0	0	0
male	78	2015-2018	CTR	408170	smoker	8	1	0	0	1	0	0	0
female	69	2015-2018	CTR	408170	ex-smoker	8	1	0	0	0	0	0	0
female	85	2015-2018	CTR	408170	non-smoker	8	0	0	0	0	0	0	0
male	69	2015-2018	CTR	408170	ex-smoker	8	1	0	0	1	0	0	1
female	58	2015-2018	CTR	408170	ex-smoker	8	1	0	0	0	0	0	0
male	59	2015-2018	CTR	408170	ex-smoker	8	0	0	0	0	0	0	0
male	71	2015-2018	CTR	408170	smoker	8	1	0	0	1	0	1	0
female	77	2015-2018	Pancreatitis	408170	non-smoker	8	1	0	0	0	2	0	1
male	74	2015-2018	Pancreatitis	408170	ex-smoker	8	1	0	0	1	2	0	0
male	54	2015-2018	Pancreatitis	408170	non-smoker	8	1	1	2	0	2	0	0
male	79	2015-2018	PC	1679718	smoker	2	0	0	0	0	0	0	0
male	62	2015-2018	PC	1679718	ex-smoker	2	1	0	0	0	0	0	0
female	69	2015-2018	PC	1679718	smoker	2	0	0	0	0	0	0	1
male	54	2015-2018	PC	1679718	non-smoker	2	1	0	0	0	0	0	0
female	68	2015-2018	PC	1679718	non-smoker	2	1	0	0	NA	0	0	0
male	68	2015-2018	PC	1679718	ex-smoker	2	1	1	1	0	0	0	0
male	71	2015-2018	PC	1679718	non-smoker	2	0	0	0	0	0	0	0
male	84	2015-2018	PC	1679718	non-smoker	2	1	1	1	0	0	0	0
female	69	2015-2018	PC	1679718	non-smoker	2	0	0	0	0	0	1	1
male	66	2015-2018	PC	1679718	smoker	2	1	0	0	0	0	0	1
male	82	2015-2018	PC	1679718	ex-smoker	2	1	1	2	0	0	0	0
male	70	2015-2018	PC	1679718	non-smoker	2	1	1	2	1	0	0	0
male	60	2015-2018	PC	1679718	smoker	2	1	0	0	0	2	0	0
male	63	2015-2018	PC	1679718	ex-smoker	2	1	0	0	1	0	0	0
male	45	2015-2018	PC	1679718	ex-smoker	2	1	0	0	0	NA	1	0
female	73	2015-2018	PC	1679718	non-smoker	2	1	0	0	1	0	0	0
female	67	2015-2018	PC	1679718	non-smoker	2	0	0	0	0	0	0	0
male	66	2015-2018	PC	1679718	non-smoker	2	0	1	2	0	0	0	0
male	68	2015-2018	PC	1679718	smoker	2	1	1	1	0	0	0	0
male	52	2015-2018	PC	1679718	smoker	2	1	0	0	1	0	0	1
male	68	2015-2018	PC	1679718	non-smoker	2	1	0	0	0	0	0	0
male	83	2015-2018	CTR	1679718	ex-smoker	2	1	0	0	NA	0	0	0
male	57	2015-2018	CTR	1679718	non-smoker	2	1	0	0	0	0	0	0
female	70	2015-2018	CTR	1679718	non-smoker	2	0	0	0	0	1	0	1
male	54	2015-2018	CTR	1679718	non-smoker	2	1	0	0	0	0	0	0
female	72	2015-2018	CTR	1679718	smoker	2	1	0	0	0	0	0	0
male	76	2015-2018	Pancreatitis	1679718	ex-smoker	2	1	1	2	1	2	0	0
male	67	2015-2018	CTR	1679718	non-smoker	2	1	1	2	0	0	0	0
male	82	2015-2018	CTR	1679718	non-smoker	2	0	0	0	0	0	0	0
female	64	2015-2018	CTR	1679718	non-smoker	2	1	0	0	0	0	0	0
male	63	2015-2018	CTR	1679718	ex-smoker	2	1	0	0	0	0	0	1
male	84	2015-2018	CTR	1679718	ex-smoker	2	1	1	2	1	0	0	0
male	61	2015-2018	CTR	1679718	smoker	2	1	0	0	0	0	0	0
male	53	2015-2018	CTR	1679718	ex-smoker	2	1	0	0	0	0	0	0
male	57	2015-2018	CTR	1679718	non-smoker	2	1	0	0	1	0	0	1
male	38	2015-2018	CTR	1679718	ex-smoker	2	1	0	0	0	0	0	0
female	68	2015-2018	CTR	1679718	ex-smoker	2	1	0	0	0	0	0	0
female	81	2015-2018	CTR	1679718	non-smoker	2	1	0	0	0	0	0	1
female	65	2015-2018	CTR	1679718	non-smoker	2	1	0	0	0	0	0	0
male	61	2015-2018	CTR	1679718	non-smoker	2	0	0	0	0	0	0	0
male	59	2015-2018	CTR	1679718	ex-smoker	2	1	0	0	1	0	0	0
male	56	2015-2018	CTR	1679718	smoker	2	1	0	0	0	0	0	0
male	63	2015-2018	CTR	1679718	smoker	2	1	0	0	0	0	0	1
male	63	2015-2018	CTR	1679718	smoker	2	1	0	0	0	0	0	0
male	79	2015-2018	Pancreatitis	1679718	ex-smoker	2	1	1	2	0	2	0	0
male	64	2015-2018	Pancreatitis	1679718	smoker	2	1	1	2	0	2	0	0
female	62	2015-2018	Pancreatitis	1679718	ex-smoker	2	1	1	2	1	2	1	0
male	54	2015-2018	Pancreatitis	1679718	smoker	2	1	0	0	0	2	0	0
female	58	2015-2018	Pancreatitis	1679718	smoker	2	1	1	2	0	2	0	1
male	58	2015-2018	Pancreatitis	1679718	smoker	2	1	0	0	0	2	0	0
male	67	2015-2018	Pancreatitis	1679718	smoker	2	1	0	0	0	2	0	0
male	76	2015-2018	Pancreatitis	1679718	ex-smoker	2	1	1	2	0	2	0	0
female	72	2015-2018	Pancreatitis	1679718	ex-smoker	2	1	1	2	0	2	0	0
male	58	2015-2018	Pancreatitis	1679718	smoker	2	1	1	2	1	2	0	0
male	78	2015-2018	Pancreatitis	1679718	ex-smoker	2	1	1	2	0	2	0	0
male	60	2015-2018	Pancreatitis	1679718	ex-smoker	2	1	1	2	0	1	0	1
male	58	2015-2018	Pancreatitis	1679718	smoker	2	1	1	2	0	2	0	0
male	64	2015-2018	Pancreatitis	1679718	ex-smoker	2	1	1	1	1	2	0	1

male	45	2015-2018	Pancreatitis	1679718	ex-smoker	2	1	0	0	0	2	0	1
female	83	2015-2018	Pancreatitis	1679718	smoker	2	0	1	2	0	2	0	0
female	71	2015-2018	Pancreatitis	1679718	non-smoker	2	0	0	0	0	2	1	0
male	64	2015-2018	Pancreatitis	1679718	smoker	2	1	1	2	0	2	0	0
male	61	2015-2018	Pancreatitis	1679718	smoker	2	1	1	2	0	2	0	0
male	55	2015-2018	Pancreatitis	1679718	smoker	2	1	1	2	0	2	0	0
male	62	2015-2018	Pancreatitis	1679718	smoker	2	1	1	2	0	2	0	0
female	55	2015-2018	Pancreatitis	1679718	smoker	2	1	0	0	1	2	0	0
female	78	2015-2018	PC	1679718	non-smoker	8	0	0	0	0	0	0	0
female	67	2015-2018	PC	1679718	smoker	8	0	0	0	0	0	1	1
female	74	2015-2018	PC	1679718	non-smoker	8	0	0	0	0	0	0	0
male	85	2015-2018	PC	1679718	ex-smoker	8	0	0	0	0	0	0	0
female	71	2015-2018	PC	1679718	non-smoker	8	0	1	1	1	0	0	1
female	68	2015-2018	PC	1679718	non-smoker	8	1	1	1	0	0	0	0
female	81	2015-2018	PC	1679718	ex-smoker	8	0	1	2	1	0	0	0
female	81	2015-2018	PC	1679718	non-smoker	8	1	1	1	1	0	0	0
male	86	2015-2018	PC	1679718	ex-smoker	8	1	0	0	0	0	0	0
female	71	2015-2018	PC	1679718	non-smoker	8	1	0	0	0	0	0	1
male	85	2015-2018	PC	1679718	ex-smoker	8	1	1	2	0	0	0	0
male	85	2015-2018	PC	1679718	ex-smoker	8	1	0	0	0	0	0	1
female	78	2015-2018	PC	1679718	non-smoker	8	0	0	0	1	0	0	0
female	84	2015-2018	PC	1679718	non-smoker	8	1	1	1	1	0	0	1
male	74	2015-2018	PC	1679718	non-smoker	8	1	0	0	1	0	0	1
male	88	2015-2018	PC	1679718	ex-smoker	8	1	0	0	0	0	0	1
female	79	2015-2018	PC	1679718	non-smoker	8	0	0	0	0	0	0	1
male	80	2015-2018	PC	1679718	non-smoker	8	1	1	2	0	0	0	1
male	49	2015-2018	PC	1679718	non-smoker	8	0	0	0	0	0	1	1
male	79	2015-2018	PC	1679718	smoker	8	1	0	0	0	0	0	0
female	80	2015-2018	PC	1679718	non-smoker	8	0	0	0	0	0	0	0
male	81	2015-2018	PC	1679718	non-smoker	8	0	0	0	0	0	0	0
male	86	2015-2018	PC	1679718	NA	8	1	0	0	1	0	0	0
male	70	2015-2018	PC	1679718	ex-smoker	8	1	0	0	0	0	0	0
female	85	2015-2018	PC	1679718	non-smoker	8	1	0	0	1	0	0	0
female	71	2015-2018	PC	1679718	non-smoker	8	1	0	0	0	0	0	0
male	67	2015-2018	PC	1679718	ex-smoker	8	1	0	0	0	0	0	0
male	69	2015-2018	PC	1679718	ex-smoker	8	1	1	2	1	0	0	0
female	75	2015-2018	PC	1679718	non-smoker	8	0	0	0	0	0	0	0
female	79	2015-2018	PC	1679718	non-smoker	8	0	0	0	1	0	0	0
female	74	2015-2018	PC	1679718	non-smoker	8	0	0	0	0	0	0	0
male	62	2015-2018	PC	1679718	ex-smoker	8	1	1	2	0	1	0	1
male	83	2015-2018	PC	1679718	ex-smoker	8	1	1	2	0	0	0	0
female	65	2015-2018	PC	1679718	ex-smoker	8	1	0	0	0	0	0	0
male	56	2015-2018	PC	1679718	ex-smoker	8	1	0	0	1	0	0	1
male	78	2015-2018	PC	1679718	ex-smoker	8	1	0	0	1	0	0	0
male	45	2015-2018	PC	1679718	smoker	8	1	0	0	1	0	0	1
male	81	2015-2018	PC	1679718	non-smoker	8	1	1	2	1	0	0	0
male	64	2015-2018	PC	1679718	smoker	8	1	1	1	0	0	0	0
female	88	2015-2018	CTR	1679718	non-smoker	8	1	0	0	NA	0	0	0
female	68	2015-2018	CTR	1679718	ex-smoker	8	1	0	0	0	0	0	0
female	80	2015-2018	CTR	1679718	non-smoker	8	0	1	2	1	0	1	1
male	88	2015-2018	CTR	1679718	ex-smoker	8	1	0	0	0	0	0	0
female	79	2015-2018	CTR	1679718	non-smoker	8	1	0	0	1	0	0	0
female	59	2015-2018	CTR	1679718	non-smoker	8	0	0	0	1	0	0	0
female	77	2015-2018	CTR	1679718	non-smoker	8	1	0	0	1	0	0	0
female	84	2015-2018	CTR	1679718	non-smoker	8	0	0	0	0	0	0	0
female	84	2015-2018	CTR	1679718	non-smoker	8	0	0	0	0	0	0	0
male	91	2015-2018	CTR	1679718	non-smoker	8	1	0	0	0	0	0	1
female	62	2015-2018	CTR	1679718	non-smoker	8	1	0	0	0	0	0	0
female	62	2015-2018	CTR	1679718	non-smoker	8	1	0	0	0	0	0	0
male	81	2015-2018	CTR	1679718	ex-smoker	8	1	1	2	0	0	0	0
male	81	2015-2018	CTR	1679718	ex-smoker	8	1	0	0	0	0	0	1
female	76	2015-2018	CTR	1679718	non-smoker	8	0	0	0	0	0	0	0
female	78	2015-2018	CTR	1679718	non-smoker	8	0	1	2	0	0	0	1
male	71	2015-2018	CTR	1679718	non-smoker	8	1	0	0	1	0	0	0
male	93	2015-2018	CTR	1679718	smoker	8	1	0	0	1	0	1	0
female	85	2015-2018	CTR	1679718	smoker	8	0	0	0	0	0	0	0
male	72	2015-2018	CTR	1679718	smoker	8	1	0	0	1	0	0	0
male	49	2015-2018	CTR	1679718	non-smoker	8	1	0	0	0	0	0	1
female	78	2015-2018	CTR	1679718	smoker	8	1	NA	0	0	0	0	0
female	87	2015-2018	CTR	1679718	non-smoker	8	0	1	2	1	0	0	0
male	87	2015-2018	CTR	1679718	ex-smoker	8	1	0	0	0	0	0	0
male	87	2015-2018	CTR	1679718	ex-smoker	8	1	0	0	0	0	0	0
male	78	2015-2018	CTR	1679718	smoker	8	1	0	0	1	0	0	0
female	88	2015-2018	CTR	1679718	non-smoker	8	0	0	0	0	0	0	0
female	69	2015-2018	CTR	1679718	ex-smoker	8	1	0	0	0	0	0	0
male	73	2015-2018	CTR	1679718	ex-smoker	8	1	0	0	0	0	0	0
male	77	2015-2018	CTR	1679718	non-smoker	8	1	0	0	0	0	0	0
female	85	2015-2018	CTR	1679718	non-smoker	8	0	0	0	0	0	0	0
female	89	2015-2018	CTR	1679718	non-smoker	8	1	0	0	0	0	1	0
male	69	2015-2018	CTR	1679718	ex-smoker	8	1	0	0	1	0	0	1
female	58	2015-2018	CTR	1679718	ex-smoker	8	1	0	0	0	0	0	0
male	59	2015-2018	CTR	1679718	ex-smoker	8	0	0	0	0	0	0	0
male	71	2015-2018	CTR	1679718	smoker	8	1	0	0	1	0	1	0
female	77	2015-2018	Pancreatitis	1679718	non-smoker	8	1	0	0	0	2	0	1
female	51	2015-2018	Pancreatitis	1679718	ex-smoker	8	1	0	0	0	2	0	1
female	51	2015-2018	Pancreatitis	1679718	ex-smoker	8	1	0	0	0	2	0	1
female	56	2015-2018	Pancreatitis	1679718	non-smoker	8	1	0	0	0	2	0	1
male	74	2015-2018	Pancreatitis	1679718	ex-smoker	8	1	0	0	1	2	0	0
male	54	2015-2018	Pancreatitis	1679718	non-smoker	8	1	1	2	0	2	0	0
male	68	2015-2018	Pancreatitis	1679718	smoker	8	0	1	2	1	2	0	0

male	79	2015-2017	PC	646099	smoker	2	0	0	0	0	0	0	0
male	79	2015-2017	PC	646099	smoker	2	0	0	0	0	0	0	0
female	68	2015-2017	PC	646099	smoker	2	0	0	0	0	0	0	1
male	54	2015-2017	PC	646099	non-smoker	2	1	0	0	0	0	0	0
female	68	2015-2017	PC	646099	non-smoker	2	1	0	0	1	0	0	0
female	68	2015-2017	PC	646099	non-smoker	2	1	0	0	1	0	0	0
male	71	2015-2017	PC	646099	non-smoker	2	0	0	0	0	0	0	0
male	84	2015-2017	PC	646099	non-smoker	2	1	1	1	0	0	0	0
male	84	2015-2017	PC	646099	non-smoker	2	1	1	1	0	0	0	0
female	69	2015-2017	PC	646099	non-smoker	2	0	0	0	0	0	1	1
female	69	2015-2017	PC	646099	non-smoker	2	0	0	0	0	0	1	1
male	82	2015-2017	PC	646099	ex-smoker	2	1	1	2	0	0	0	0
male	82	2015-2017	PC	646099	ex-smoker	2	1	1	2	0	0	0	0
male	82	2015-2017	PC	646099	ex-smoker	2	1	1	2	0	0	0	0
male	60	2015-2017	PC	646099	smoker	2	1	0	0	0	2	0	0
male	63	2015-2017	PC	646099	ex-smoker	2	1	0	0	1	0	0	0
male	63	2015-2017	PC	646099	ex-smoker	2	1	0	0	1	0	0	0
male	63	2015-2017	PC	646099	ex-smoker	2	1	0	0	1	0	0	0
male	63	2015-2017	PC	646099	ex-smoker	2	1	0	0	1	0	0	0
male	45	2015-2017	PC	646099	ex-smoker	2	1	0	0	0	0	1	0
female	73	2015-2017	PC	646099	non-smoker	2	1	0	0	1	0	0	0
female	73	2015-2017	PC	646099	non-smoker	2	1	0	0	1	0	0	0
male	66	2015-2017	PC	646099	non-smoker	2	0	1	2	0	0	0	0
male	66	2015-2017	PC	646099	non-smoker	2	0	1	2	0	0	0	0
male	68	2015-2017	PC	646099	non-smoker	2	1	0	0	0	0	0	0
male	68	2015-2017	PC	646099	non-smoker	2	1	0	0	0	0	0	0
male	79	2015-2017	Pancreatitis	646099	ex-smoker	2	1	1	2	0	2	0	0
male	79	2015-2017	Pancreatitis	646099	ex-smoker	2	1	1	2	0	2	0	0
male	54	2015-2017	Pancreatitis	646099	smoker	2	1	0	0	0	1	0	0
male	54	2015-2017	Pancreatitis	646099	smoker	2	1	0	0	0	1	0	0
male	54	2015-2017	Pancreatitis	646099	smoker	2	1	0	0	0	1	0	0
male	58	2015-2017	Pancreatitis	646099	smoker	2	1	0	0	0	0	0	0
male	58	2015-2017	Pancreatitis	646099	smoker	2	1	0	0	0	0	0	0
female	55	2015-2017	Pancreatitis	646099	smoker	2	1	0	0	1	0	0	0
female	78	2015-2017	PC	646099	non-smoker	8	0	0	0	0	0	0	0
female	67	2015-2017	PC	646099	smoker	8	0	0	0	0	0	1	1
female	67	2015-2017	PC	646099	smoker	8	0	0	0	0	0	1	1
female	74	2015-2017	PC	646099	non-smoker	8	0	0	0	0	0	0	0
female	71	2015-2017	PC	646099	non-smoker	8	0	1	1	1	0	0	1
female	71	2015-2017	PC	646099	non-smoker	8	0	1	1	1	0	0	1
female	81	2015-2017	PC	646099	ex-smoker	8	0	1	2	1	0	0	0
female	81	2015-2017	PC	646099	ex-smoker	8	0	1	2	1	0	0	0
male	85	2015-2017	PC	646099	ex-smoker	8	1	1	2	0	0	0	0
male	85	2015-2017	PC	646099	ex-smoker	8	1	1	2	0	0	0	0
male	85	2015-2017	PC	646099	ex-smoker	8	1	1	2	0	0	0	0
female	78	2015-2017	PC	646099	non-smoker	8	0	0	0	1	0	0	0
female	78	2015-2017	PC	646099	non-smoker	8	0	0	0	1	0	0	0
female	79	2015-2017	PC	646099	non-smoker	8	0	0	0	0	0	0	1
female	79	2015-2017	PC	646099	non-smoker	8	0	0	0	0	0	0	1
female	79	2015-2017	PC	646099	non-smoker	8	0	0	0	0	0	0	1
male	81	2015-2017	PC	646099	non-smoker	8	1	1	2	1	0	0	0

allhburn	allrheum	allhbp	cholesterol	abmedicatior	antibiotic	asparmed	salicylic.ever	racetamol.ev	holmedicatio	cortmed	diabdiet	diabin	diabmed
0	0	1	0	0	1	1	0	0	NA	0	0	0	0
0	0	0	0	0	0	0	0	0	NA	0	0	0	0
0	0	0	1	1	1	1	0	0	1	0	0	0	0
0	0	1	0	0	0	0	0	0	NA	0	0	0	0
0	0	1	0	0	0	0	0	0	NA	0	0	0	0
0	0	1	0	0	1	0	0	0	NA	0	0	0	0
0	0	1	0	0	1	0	0	0	NA	0	1	1	2
0	0	1	0	0	1	0	0	0	NA	0	1	1	2
0	0	1	1	1	1	1	0	1	1	0	0	0	0
0	0	1	0	1	1	1	1	0	NA	0	1	1	1
0	0	1	0	1	1	1	1	0	NA	0	1	1	1
1	0	1	1	1	1	0	0	0	0	0	0	0	0
1	0	0	0	0	0	0	0	0	NA	0	0	0	0
0	0	1	0	0	0	1	1	0	NA	0	2	2	1
0	0	1	0	0	0	1	1	0	NA	0	2	2	1
0	0	1	1	0	1	0	0	0	1	0	1	2	1
0	0	1	1	0	1	0	0	0	1	0	1	2	1
0	0	1	0	0	0	0	0	0	NA	0	0	0	0
0	0	1	1	0	1	0	0	0	1	0	0	0	0
1	0	0	0	1	1	0	0	0	0	0	0	0	0
0	0	1	0	0	0	0	0	0	NA	0	0	0	0
0	0	1	0	0	0	1	0	1	NA	0	0	0	0
0	0	1	0	0	0	1	0	1	NA	0	0	0	0
0	0	0	0	0	1	0	0	0	NA	0	1	2	1
0	0	1	1	1	0	1	0	0	1	0	1	1	2
1	0	1	0	0	0	0	0	0	NA	0	0	0	0
0	0	0	0	0	1	0	0	0	NA	0	0	0	0
1	0	1	0	1	1	1	1	1	NA	0	0	0	0
0	0	0	1	0	1	1	0	0	0	0	0	0	0
0	0	0	1	0	1	1	0	0	0	0	0	0	0
1	0	0	0	0	1	0	0	0	NA	0	0	0	0
0	0	0	0	0	1	0	0	0	NA	0	0	0	0
0	1	0	1	0	0	0	0	0	1	0	0	0	0
0	0	1	1	1	1	1	0	1	1	0	2	1	2
0	0	0	0	0	0	0	0	0	NA	0	1	2	1
0	0	0	0	0	0	0	0	0	NA	0	1	2	1
0	0	0	0	0	0	0	0	0	NA	0	0	0	0
0	0	0	0	0	0	0	0	0	NA	0	0	0	0
1	0	0	0	0	0	0	0	0	NA	0	0	0	0
0	0	0	0	1	1	0	0	0	NA	0	1	2	1
0	0	1	1	0	0	0	0	0	0	0	0	0	0
0	0	0	0	0	0	0	0	0	NA	0	0	0	0
1	0	0	0	1	1	0	0	0	NA	0	0	0	0
0	0	0	0	0	1	0	0	0	NA	0	0	0	0
0	0	1	0	0	0	0	0	0	0	0	0	0	0
1	0	0	0	0	0	1	1	0	NA	0	0	0	0
0	0	1	1	0	1	0	0	0	1	0	0	0	0
0	0	0	0	0	0	0	0	0	NA	0	0	0	0
0	0	0	1	0	1	1	1	0	0	0	0	0	0
1	0	0	1	0	1	0	0	0	0	0	0	0	0
1	0	0	0	1	0	1	0	0	NA	0	0	0	0
0	0	0	0	0	0	0	0	0	NA	0	0	0	0
0	0	1	0	0	0	1	0	1	NA	0	2	2	1
0	0	1	0	0	1	0	0	0	NA	0	1	1	2
1	0	1	0	1	0	0	0	0	0	0	1	1	1
0	0	1	0	0	1	0	0	0	NA	0	0	0	0
1	0	0	1	1	0	1	0	0	1	1	2	1	2
0	0	1	0	0	1	1	0	1	NA	0	0	0	0
0	0	0	0	0	0	0	0	0	0	0	0	0	0
0	0	1	0	1	1	1	0	0	0	0	1	1	1
0	0	1	1	1	1	1	0	0	1	0	1	1	1
0	0	0	0	0	0	0	0	0	NA	0	1	2	1
0	0	1	0	0	0	0	0	0	0	0	1	2	1
1	0	0	1	1	1	0	0	0	1	0	1	1	2
0	0	1	1	1	1	0	0	0	1	0	1	1	1
1	0	0	1	1	1	0	0	0	0	0	1	2	2
1	0	0	1	1	1	0	0	0	0	0	1	2	2
0	0	0	0	0	0	0	0	0	NA	0	0	0	0
0	0	1	0	1	0	0	0	0	0	0	1	1	1
0	0	0	1	1	1	1	0	1	1	0	0	0	0
1	0	0	0	1	1	1	0	1	0	0	2	1	1
0	0	0	1	1	0	0	0	0	1	0	1	1	1
0	0	0	0	1	1	1	0	1	NA	0	2	1	2
0	0	1	0	1	0	0	0	0	0	0	1	1	1
0	0	1	0	1	1	1	1	1	NA	0	0	0	0
1	0	0	1	1	1	0	0	0	1	0	0	0	0
1	0	0	1	1	1	0	0	0	1	0	0	0	0
1	1	1	1	0	1	1	1	1	1	0	0	0	0
0	1	1	1	NA	1	1	0	1	1	0	0	0	0
0	1	1	1	NA	1	1	0	1	1	0	0	0	0
0	0	1	1	0	1	1	0	1	1	0	0	0	0
1	1	0	1	0	1	0	0	0	0	0	1	2	2
0	0	0	1	0	1	0	0	0	1	0	1	2	1
0	0	1	1	0	NA	0	0	0	1	0	2	2	1
0	0	NA	1	0	0	1	0	1	0	0	0	0	0
1	0	0	1	0	1	0	0	0	1	0	0	0	0
1	0	1	0	1	1	0	0	0	NA	0	2	2	1
1	0	1	1	1	0	0	0	0	1	0	0	0	0

1	0	1	0	1	1	1	0	1	0	1	2	1	2
1	0	1	NA	1	1	0	0	0	1	0	0	0	0
0	0	1	1	0	0	1	1	1	1	1	0	0	0
1	0	0	0	1	0	0	0	0	0	0	0	0	0
1	0	0	0	1	0	0	0	0	0	0	0	0	0
0	1	1	0	0	1	1	0	1	0	0	1	1	1
0	1	0	1	1	0	1	0	1	0	1	0	0	0
0	0	0	0	0	0	0	0	0	0	0	0	0	0
0	0	1	0	1	1	1	0	1	0	0	0	0	0
NA	0	0	0	0	0	1	1	1	0	0	0	0	0
NA	0	0	0	0	0	1	1	1	0	0	0	0	0
0	0	1	1	0	1	0	0	0	1	0	0	0	0
0	0	0	NA	0	0	1	0	1	1	0	0	0	0
0	0	1	1	1	1	1	0	0	1	0	0	0	0
0	0	0	NA	1	1	1	1	0	0	0	0	0	0
0	0	1	0	1	0	0	0	0	1	0	1	2	1
0	0	1	1	0	1	0	0	0	1	0	0	0	0
0	0	0	0	1	0	0	0	0	1	0	0	0	0
0	0	0	0	1	0	0	0	0	0	1	0	0	0
1	0	0	1	0	1	1	0	0	1	0	1	1	1
0	0	0	NA	NA	1	1	0	1	NA	0	1	1	1
0	0	0	0	0	0	0	0	0	0	0	0	0	0
1	0	1	0	1	0	1	0	0	0	1	0	0	0
0	0	1	1	0	0	0	0	0	1	0	0	0	0
0	0	0	1	1	1	1	1	1	0	0	0	0	0
1	0	1	1	0	0	0	0	0	1	0	2	2	1
0	0	0	0	0	1	0	0	0	0	0	1	1	2
0	1	0	0	NA	0	1	0	0	NA	NA	0	0	0
1	0	1	1	0	1	1	0	1	1	1	1	1	2
0	0	1	0	1	1	1	1	1	0	0	0	0	0
0	0	1	0	0	0	0	0	0	0	0	0	0	0
0	1	1	0	0	1	1	0	1	0	1	0	0	0
0	0	0	0	1	1	0	0	0	0	0	0	0	0
0	1	1	0	1	0	1	1	1	0	0	0	0	0
0	1	0	1	0	1	0	0	0	1	0	1	2	2
0	0	1	0	0	0	0	0	0	0	0	0	0	0
0	1	1	1	1	0	1	1	0	1	0	0	0	0
0	0	1	0	NA	1	0	0	0	NA	0	0	0	0
1	0	0	1	0	1	0	0	0	1	0	0	0	0
0	1	0	0	1	1	1	0	0	0	1	0	0	0
0	0	1	1	0	1	0	0	0	1	0	0	0	0
1	0	0	0	0	1	0	0	0	0	0	0	0	0
0	0	1	0	1	0	1	1	1	0	0	1	2	1
0	0	0	0	0	0	1	1	1	0	0	0	0	0
0	0	1	0	0	0	1	1	0	0	0	0	0	0
0	0	1	0	0	0	1	1	0	0	0	0	0	0
0	1	1	0	1	1	1	0	1	1	0	0	0	0
0	0	0	0	0	1	0	0	0	0	0	0	0	0
0	1	1	NA	0	1	0	0	0	1	0	0	0	0
0	0	1	0	0	1	1	NA	NA	0	0	0	0	0
0	1	0	0	0	0	0	0	0	0	0	0	0	0
0	0	0	0	1	1	1	0	0	0	0	NA	NA	NA
0	0	0	0	1	1	1	0	1	0	0	0	0	0
0	0	1	1	0	1	0	0	0	1	0	0	0	0
0	0	1	0	0	0	0	0	0	0	0	0	0	0
0	0	1	1	0	0	0	0	0	1	0	0	0	0
1	1	1	0	0	0	1	0	0	NA	0	0	0	0
0	1	0	0	1	1	1	0	0	NA	0	0	0	0
1	0	0	NA	1	1	1	NA	NA	0	1	0	0	0
0	0	0	0	1	1	1	0	1	0	0	0	0	0
1	0	0	0	1	1	1	0	0	0	0	2	1	2
0	0	0	0	1	1	1	1	0	0	0	1	2	1
0	0	1	0	0	1	1	0	0	NA	0	0	0	0
0	0	0	0	0	0	0	0	0	NA	0	0	0	0
0	0	0	1	1	1	1	0	0	1	0	0	0	0
0	0	1	0	0	0	0	0	0	NA	0	0	0	0
0	0	1	0	0	1	0	0	0	NA	0	0	0	0
0	0	1	0	0	1	0	0	0	NA	0	1	1	2
0	0	1	1	1	1	1	0	1	1	0	0	0	0
0	0	1	0	1	1	1	1	0	NA	0	1	1	1
1	0	1	1	1	1	0	0	0	0	0	0	0	0
1	0	0	0	0	0	0	0	0	NA	0	0	0	0
0	0	1	0	0	0	1	1	0	NA	0	2	2	1
0	0	1	1	0	1	0	0	0	1	0	1	2	1
0	0	1	0	0	0	0	0	0	NA	0	0	0	0
0	0	1	1	0	1	0	0	0	1	0	0	0	0
1	0	0	0	1	1	0	0	0	0	0	0	0	0
0	0	1	0	0	0	0	0	0	NA	0	0	0	0
0	0	1	0	0	0	1	0	1	NA	0	0	0	0
0	0	0	0	0	1	0	0	0	NA	0	1	2	1
0	0	1	1	1	0	1	0	0	1	0	1	1	2
1	0	1	0	0	0	0	0	0	NA	0	0	0	0
0	0	0	0	0	1	0	0	0	NA	0	0	0	0
1	0	1	0	1	1	1	1	1	NA	0	0	0	0
0	0	0	1	0	1	1	0	0	0	0	0	0	0
1	0	0	0	0	1	0	0	0	NA	0	0	0	0
0	0	0	0	0	1	0	0	0	NA	0	0	0	0
0	1	0	1	0	0	0	0	0	1	0	0	0	0
0	0	1	1	1	1	1	0	1	1	0	2	1	2
0	0	0	0	0	0	0	0	0	NA	0	1	2	1

0	0	0	0	0	0	0	0	0	NA	0	0	0	0
0	0	0	0	0	0	0	0	0	NA	0	0	0	0
1	0	0	0	0	0	0	0	0	NA	0	0	0	0
0	0	0	0	1	1	0	0	0	NA	0	1	2	1
0	0	1	1	0	0	0	0	0	0	0	0	0	0
0	0	0	0	0	0	0	0	0	NA	0	0	0	0
1	0	0	0	1	1	0	0	0	NA	0	0	0	0
0	0	0	0	0	1	0	0	0	NA	0	0	0	0
0	0	1	0	0	0	0	0	0	0	0	0	0	0
1	0	0	0	0	0	1	1	0	NA	0	0	0	0
0	0	1	1	0	1	0	0	0	1	0	0	0	0
0	0	0	0	0	0	0	0	0	NA	0	0	0	0
0	0	0	1	0	1	1	1	0	0	0	0	0	0
1	0	0	1	0	1	0	0	0	0	0	0	0	0
1	0	0	0	1	0	1	0	0	NA	0	0	0	0
0	0	0	0	0	0	0	0	0	NA	0	0	0	0
0	0	1	0	0	0	1	0	1	NA	0	2	2	1
0	0	1	0	0	1	0	0	0	NA	0	1	1	2
0	0	1	0	0	1	0	0	0	NA	0	0	0	0
0	0	1	0	0	1	1	0	1	NA	0	0	0	0
0	0	0	0	0	0	0	0	0	NA	0	1	2	1
0	0	1	1	1	1	0	0	0	1	0	1	1	1
1	0	0	1	1	1	0	0	0	0	0	1	2	2
0	0	1	0	1	1	1	1	1	NA	0	0	0	0
1	0	0	1	1	1	0	0	0	1	0	0	0	0
1	1	1	1	0	1	1	1	1	1	0	0	0	0
0	1	1	1	NA	1	1	0	1	1	0	0	0	0
0	0	1	1	0	1	1	0	1	1	0	0	0	0
1	1	0	1	0	1	0	0	0	0	0	1	2	2
0	0	0	1	0	1	0	0	0	1	0	1	2	1
0	NA	1	1	0	1	1	0	0	1	0	1	1	1
0	0	1	1	0	NA	0	0	0	1	0	2	2	1
0	0	NA	1	0	0	1	0	1	0	0	0	0	0
1	0	0	1	0	1	0	0	0	1	0	0	0	0
1	0	1	0	1	1	0	0	0	NA	0	2	2	1
1	0	1	1	1	0	0	0	0	1	0	0	0	0
0	0	0	0	0	1	0	0	0	NA	0	0	0	0
1	0	1	0	1	1	1	0	1	0	1	2	1	2
1	0	1	NA	1	1	0	0	0	1	0	0	0	0
0	0	1	1	0	0	1	1	1	1	1	0	0	0
1	0	0	0	1	0	0	0	0	0	0	0	0	0
0	1	1	0	0	1	1	0	1	0	0	1	1	1
0	1	0	1	1	0	1	0	1	0	1	0	0	0
0	0	1	0	1	1	1	0	1	0	0	0	0	0
0	0	0	1	0	1	0	0	0	1	0	0	0	0
NA	0	0	0	0	0	1	1	1	0	0	0	0	0
0	1	0	0	NA	0	1	0	0	NA	NA	0	0	0
0	1	1	0	0	1	0	0	0	NA	0	0	0	0
1	0	1	1	0	1	1	0	1	1	1	1	1	2
0	0	1	0	1	1	1	1	1	0	0	0	0	0
0	0	1	0	0	0	0	0	0	0	0	0	0	0
0	1	1	0	0	1	1	0	1	0	1	0	0	0
0	0	1	0	0	0	1	0	1	NA	1	0	0	0
0	0	1	1	0	0	0	0	0	1	0	0	0	0
0	0	1	1	0	0	0	0	0	1	0	0	0	0
0	0	0	0	1	1	0	0	0	0	0	0	0	0
0	1	1	0	1	0	1	1	1	0	0	0	0	0
0	1	1	0	1	0	1	1	1	0	0	0	0	0
0	1	0	1	0	1	0	0	0	1	0	1	2	2
0	0	1	0	0	0	0	0	0	0	0	0	0	0
0	1	1	1	1	0	1	1	0	1	0	0	0	0
0	0	1	1	1	1	1	1	0	1	NA	1	1	1
0	0	1	0	NA	1	0	0	0	NA	0	0	0	0
1	0	0	1	0	1	0	0	0	1	0	0	0	0
0	1	0	0	1	1	1	0	0	0	1	0	0	0
0	0	1	1	0	1	0	0	0	1	0	0	0	0
1	0	0	0	0	1	0	0	0	0	0	0	0	0
0	0	0	0	0	0	0	NA	NA	0	0	0	0	0
0	0	1	0	1	0	1	1	1	0	0	1	2	1
0	0	0	0	0	0	1	1	1	0	0	0	0	0
0	0	0	0	0	0	1	1	1	0	0	0	0	0
0	0	1	0	0	0	1	1	0	0	0	0	0	0
1	1	1	0	0	0	1	0	0	NA	0	0	0	0
0	0	0	0	1	1	1	0	1	0	0	0	0	0
1	0	0	0	1	1	1	0	0	0	0	2	1	2
0	0	0	1	1	1	1	0	0	1	0	0	0	0
0	0	1	0	0	0	0	0	0	0	0	0	0	0
0	0	1	0	0	1	0	0	0	0	0	0	0	0
0	0	1	1	1	1	1	0	1	1	0	0	0	0
0	0	1	0	0	0	0	0	0	0	0	0	0	0
0	0	1	1	0	1	0	0	0	1	0	0	0	0
0	0	1	1	0	1	0	0	0	1	0	0	0	0
1	0	0	0	1	1	0	0	0	0	0	0	0	0
1	0	0	0	1	1	0	0	0	0	0	0	0	0
0	0	1	0	0	0	0	0	0	0	0	0	0	0
0	0	0	0	0	1	0	0	0	0	0	1	2	1
0	0	0	0	0	1	0	0	0	0	0	0	0	0
0	0	1	0	0	0	1	0	1	0	0	2	2	1
0	0	1	0	0	1	0	0	0	0	0	0	0	0
0	0	1	0	0	1	0	0	0	0	0	0	0	0

0	0	1	0	0	1	1	0	0	NA	0	0	0	0
0	0	0	0	0	0	0	0	0	NA	0	0	0	0
0	0	0	1	1	1	1	0	0	1	0	0	0	0
0	0	1	0	0	0	0	0	0	NA	0	0	0	0
0	0	1	0	0	1	0	0	0	NA	0	0	0	0
0	0	1	0	0	1	0	0	0	NA	0	1	1	2
0	0	1	1	1	1	1	0	1	1	0	0	0	0
0	0	1	0	1	1	1	1	0	NA	0	1	1	1
1	0	1	1	1	1	0	0	0	0	0	0	0	0
1	0	0	0	0	0	0	0	0	NA	0	0	0	0
0	0	1	0	0	0	1	1	0	NA	0	2	2	1
0	0	1	1	0	1	0	0	0	1	0	1	2	1
0	0	1	0	0	0	0	0	0	NA	0	0	0	0
0	0	1	1	0	1	0	0	0	1	0	0	0	0
1	0	0	0	1	1	0	0	0	0	0	0	0	0
0	0	1	0	0	0	0	0	0	NA	0	0	0	0
0	0	1	0	0	0	1	0	1	NA	0	0	0	0
0	0	0	0	0	1	0	0	0	NA	0	1	2	1
0	0	1	1	1	0	1	0	0	1	0	1	1	2
1	0	1	0	0	0	0	0	0	NA	0	0	0	0
0	0	0	0	0	1	0	0	0	NA	0	0	0	0
1	0	1	0	1	1	1	1	1	NA	0	0	0	0
0	0	0	1	0	1	1	0	0	0	0	0	0	0
1	0	0	0	0	1	0	0	0	NA	0	0	0	0
0	0	0	0	0	1	0	0	0	NA	0	0	0	0
0	1	0	1	0	0	0	0	0	1	0	0	0	0
0	0	1	1	1	1	1	0	1	1	0	2	1	2
0	0	0	0	0	0	0	0	0	NA	0	1	2	1
0	0	0	0	0	0	0	0	0	NA	0	0	0	0
0	0	0	0	0	0	0	0	0	NA	0	0	0	0
1	0	0	0	0	0	0	0	0	NA	0	0	0	0
0	0	0	0	1	1	0	0	0	NA	0	1	2	1
0	0	1	1	0	0	0	0	0	0	0	0	0	0
0	0	0	0	0	0	0	0	0	NA	0	0	0	0
1	0	0	0	1	1	0	0	0	NA	0	0	0	0
0	0	0	0	0	1	0	0	0	NA	0	0	0	0
0	0	1	0	0	0	0	0	0	0	0	0	0	0
1	0	0	0	0	0	1	1	0	NA	0	0	0	0
0	0	1	1	0	1	0	0	0	1	0	0	0	0
0	0	0	0	0	0	0	0	0	NA	0	0	0	0
0	0	0	1	0	1	1	1	0	0	0	0	0	0
1	0	0	1	0	1	0	0	0	0	0	0	0	0
1	0	0	0	1	0	1	0	0	NA	0	0	0	0
0	0	0	0	0	0	0	0	0	NA	0	0	0	0
0	0	1	0	0	0	1	0	1	NA	0	2	2	1
0	0	1	0	0	1	0	0	0	NA	0	1	1	2
1	0	1	0	1	0	0	0	0	0	0	1	1	1
0	0	1	0	0	1	0	0	0	NA	0	0	0	0
0	0	1	0	0	1	1	0	1	NA	0	0	0	0
0	0	0	0	0	0	0	0	0	0	0	0	0	0
0	0	1	0	1	1	1	0	0	0	0	1	1	1
0	0	1	1	1	1	1	0	0	1	0	1	1	1
0	0	0	0	0	0	0	0	0	NA	0	1	2	1
0	0	1	0	0	0	0	0	0	0	0	1	2	1
1	0	0	1	1	1	0	0	0	1	0	1	1	2
0	0	1	1	1	1	0	0	0	1	0	1	1	1
1	0	0	1	1	1	0	0	0	0	0	1	2	2
1	0	0	1	1	1	0	0	0	0	0	1	2	2
0	0	1	0	1	0	0	0	0	0	0	1	1	1
0	0	0	1	1	1	1	0	1	1	0	0	0	0
1	0	0	0	1	1	1	0	1	0	0	2	1	1
0	0	0	1	1	0	0	0	0	1	0	1	1	1
0	0	1	0	1	0	0	0	0	0	0	1	1	1
0	0	1	0	1	1	1	1	1	NA	0	0	0	0
1	0	0	1	1	1	0	0	0	1	0	0	0	0
1	1	1	1	0	1	1	1	1	1	0	0	0	0
0	1	1	1	NA	1	1	0	1	1	0	0	0	0
0	0	1	1	0	1	1	0	1	1	0	0	0	0
1	1	0	1	0	1	0	0	0	0	0	1	2	2
0	0	0	1	0	1	0	0	0	1	0	1	2	1
0	0	1	1	0	NA	0	0	0	1	0	2	2	1
0	0	NA	1	0	0	1	0	1	0	0	0	0	0
1	0	0	1	0	1	0	0	0	1	0	0	0	0
1	0	1	0	1	1	0	0	0	NA	0	2	2	1
1	0	1	1	1	0	0	0	0	1	0	0	0	0
1	0	1	0	1	1	1	0	1	0	1	2	1	2
1	0	1	NA	1	1	0	0	0	1	0	0	0	0
0	0	1	1	0	0	1	1	1	1	1	0	0	0
1	0	0	0	1	0	0	0	0	0	0	0	0	0
0	1	1	0	0	1	1	0	1	0	0	1	1	1
0	1	0	1	1	0	1	0	1	0	1	0	0	0
0	0	0	0	0	0	0	0	0	0	0	0	0	0
0	0	1	0	1	1	1	0	1	0	0	0	0	0
0	0	0	1	0	1	0	0	0	1	0	0	0	0
0	0	0	1	0	1	0	0	0	1	0	0	0	0
NA	0	0	0	0	0	1	1	1	0	0	0	0	0
0	0	1	1	0	1	0	0	0	1	0	0	0	0
0	0	0	NA	0	0	1	0	1	1	0	0	0	0
0	0	1	1	1	1	1	0	0	1	0	0	0	0
0	0	0	0	0	0	0	0	0	0	0	0	0	0

1	0	1	0	1	0	1	0	0	0	1	0	0	0
0	0	1	1	0	0	0	0	0	1	0	0	0	0
0	0	0	1	1	1	1	1	1	0	0	0	0	0
1	0	1	1	0	0	0	0	0	1	0	2	2	1
0	0	0	0	0	1	0	0	0	0	0	1	1	2
0	1	0	0	NA	0	1	0	0	NA	NA	0	0	0
1	0	1	1	0	1	1	0	1	1	1	1	1	2
0	0	1	0	1	1	1	1	1	0	0	0	0	0
0	0	1	0	0	0	0	0	0	0	0	0	0	0
0	1	1	0	0	1	1	0	1	0	1	0	0	0
0	0	0	0	1	1	0	0	0	0	0	0	0	0
0	1	1	0	1	0	1	1	1	0	0	0	0	0
0	1	0	1	0	1	0	0	0	1	0	1	2	2
0	0	1	0	0	0	0	0	0	0	0	0	0	0
0	1	1	1	1	0	1	1	0	1	0	0	0	0
0	0	1	0	NA	1	0	0	0	NA	0	0	0	0
1	0	0	1	0	1	0	0	0	1	0	0	0	0
0	1	0	0	1	1	1	0	0	0	1	0	0	0
0	0	1	1	0	1	0	0	0	1	0	0	0	0
1	0	0	0	0	1	0	0	0	0	0	0	0	0
0	0	1	0	1	0	1	1	1	0	0	1	2	1
0	0	0	0	0	0	1	1	1	0	0	0	0	0
0	0	1	0	0	0	1	1	0	0	0	0	0	0
0	0	0	0	0	1	0	0	0	0	0	0	0	0
0	1	0	0	0	0	0	0	0	0	0	0	0	0
0	0	0	0	1	1	1	0	1	0	0	0	0	0
0	0	1	1	0	1	0	0	0	1	0	0	0	0
0	0	1	0	0	0	0	0	0	0	0	0	0	0
0	0	1	1	0	0	0	0	0	1	0	0	0	0
1	1	1	0	0	0	1	0	0	NA	0	0	0	0
0	0	0	0	1	1	1	0	1	0	0	0	0	0
1	0	0	0	1	1	1	0	0	0	0	2	1	2
0	0	1	0	0	1	1	0	0	NA	0	0	0	0
0	0	0	0	0	0	0	0	0	NA	0	0	0	0
0	0	0	1	1	1	1	0	0	1	0	0	0	0
0	0	1	0	0	0	0	0	0	NA	0	0	0	0
0	0	1	0	0	1	0	0	0	NA	0	0	0	0
0	0	1	0	0	1	0	0	0	NA	0	1	1	2
0	0	1	1	1	1	1	0	1	1	0	0	0	0
0	0	1	0	1	1	1	1	0	NA	0	1	1	1
1	0	1	1	1	1	0	0	0	0	0	0	0	0
1	0	0	0	0	0	0	0	0	NA	0	0	0	0
0	0	1	0	0	0	1	1	0	NA	0	2	2	1
0	0	1	1	0	1	0	0	0	1	0	1	2	1
0	0	1	0	0	0	0	0	0	NA	0	0	0	0
0	0	1	1	0	1	0	0	0	1	0	0	0	0
1	0	0	0	1	1	0	0	0	0	0	0	0	0
0	0	1	0	0	0	0	0	0	NA	0	0	0	0
0	0	1	0	0	0	1	0	1	NA	0	0	0	0
0	0	0	0	0	1	0	0	0	NA	0	1	2	1
0	0	1	1	1	0	1	0	0	1	0	1	1	2
1	0	1	0	0	0	0	0	0	NA	0	0	0	0
0	0	0	0	0	1	0	0	0	NA	0	0	0	0
1	0	1	0	1	1	1	1	1	NA	0	0	0	0
0	0	0	1	0	1	1	0	0	0	0	0	0	0
1	0	0	0	0	1	0	0	0	NA	0	0	0	0
0	0	0	0	0	1	0	0	0	NA	0	0	0	0
0	1	0	1	0	0	0	0	0	1	0	0	0	0
0	0	1	1	1	1	1	0	1	1	0	2	1	2
0	0	0	0	0	0	0	0	0	NA	0	1	2	1
0	0	0	0	0	0	0	0	0	NA	0	0	0	0
0	0	0	0	0	0	0	0	0	NA	0	0	0	0
1	0	0	0	0	0	0	0	0	NA	0	0	0	0
0	0	0	0	1	1	0	0	0	NA	0	1	2	1
0	0	1	1	0	0	0	0	0	0	0	0	0	0
0	0	0	0	0	0	0	0	0	NA	0	0	0	0
1	0	0	0	1	1	0	0	0	NA	0	0	0	0
0	0	0	0	0	1	0	0	0	NA	0	0	0	0
0	0	1	0	0	0	0	0	0	0	0	0	0	0
1	0	0	0	0	0	1	1	0	NA	0	0	0	0
0	0	1	1	0	1	0	0	0	1	0	0	0	0
0	0	0	0	0	0	0	0	0	NA	0	0	0	0
0	0	0	1	0	1	1	1	0	0	0	0	0	0
1	0	0	1	0	1	0	0	0	0	0	0	0	0
1	0	0	0	1	0	1	0	0	NA	0	0	0	0
0	0	0	0	0	0	0	0	0	NA	0	0	0	0
0	0	1	0	0	0	1	0	1	NA	0	2	2	1
0	0	1	0	0	1	0	0	0	NA	0	1	1	2
1	0	1	0	1	0	0	0	0	NA	0	1	1	1
0	0	1	0	0	1	0	0	0	NA	0	0	0	0
1	0	0	1	1	0	1	0	0	1	1	2	1	2
0	0	1	0	0	1	1	0	1	NA	0	0	0	0
0	0	0	0	0	0	0	0	0	0	0	0	0	0
0	0	1	0	1	1	1	0	0	0	0	1	1	1
0	0	1	1	1	1	1	0	0	1	0	1	1	1
0	0	0	0	0	0	0	0	0	NA	0	1	2	1
0	0	1	0	0	0	0	0	0	0	0	1	2	1
1	0	0	1	1	1	0	0	0	1	0	1	1	2
0	0	1	1	1	1	0	0	0	1	0	1	1	1
1	0	0	1	1	1	0	0	0	0	0	1	2	2

0	0	0	0	0	0	0	0	0	0	0	0	0	0
0	0	1	0	1	0	0	0	0	0	0	1	1	1
0	0	0	1	1	1	1	0	1	1	0	0	0	0
1	0	0	0	1	1	1	0	1	0	0	2	1	1
0	0	0	1	1	0	0	0	0	1	0	1	1	1
0	0	0	0	1	1	1	0	1	0	0	2	1	2
0	0	1	0	1	0	0	0	0	0	0	1	1	1
0	0	1	0	1	1	1	1	1	NA	0	0	0	0
1	0	0	1	1	1	0	0	0	1	0	0	0	0
1	1	1	1	0	1	1	1	1	1	0	0	0	0
0	1	1	1	NA	1	1	0	1	1	0	0	0	0
0	0	1	1	0	1	1	0	1	1	0	0	0	0
1	1	0	1	0	1	0	0	0	0	0	1	2	2
0	0	0	1	0	1	0	0	0	1	0	1	2	1
0	NA	1	1	0	1	1	0	0	1	0	1	1	1
0	0	1	1	0	NA	0	0	0	1	0	2	2	1
0	0	NA	1	0	0	1	0	1	0	0	0	0	0
1	0	0	1	0	1	0	0	0	1	0	0	0	0
1	0	1	0	1	1	0	0	0	NA	0	2	2	1
1	0	1	1	1	0	0	0	0	1	0	0	0	0
0	0	0	0	0	1	0	0	0	NA	0	0	0	0
1	0	1	0	1	1	1	0	1	0	1	2	1	2
1	0	1	NA	1	1	0	0	0	1	0	0	0	0
0	0	1	1	0	0	1	1	1	1	1	0	0	0
1	0	0	0	1	0	0	0	0	0	0	0	0	0
0	1	1	0	0	1	1	0	1	0	0	1	1	1
0	1	0	1	1	0	1	0	1	0	1	0	0	0
0	0	0	0	0	0	0	0	0	0	0	0	0	0
0	0	1	0	1	1	1	0	1	0	0	0	0	0
0	0	0	1	0	1	0	0	0	1	0	0	0	0
NA	0	0	0	0	0	1	1	1	0	0	0	0	0
0	0	1	1	0	1	0	0	0	1	0	0	0	0
0	0	0	1	0	0	1	0	1	1	0	0	0	0
0	0	1	1	1	1	1	0	0	1	0	0	0	0
0	0	0	0	1	1	1	1	0	0	0	0	0	0
0	0	1	0	1	0	0	0	0	1	0	1	2	1
0	0	1	1	0	1	0	0	0	1	0	0	0	0
0	0	0	0	1	0	0	0	0	1	0	0	0	0
0	0	0	0	1	0	0	0	0	0	1	0	0	0
1	0	0	1	0	1	1	0	0	1	0	1	1	1
0	0	0	0	1	1	1	0	1	0	1	1	1	1
0	0	0	0	0	0	0	0	0	0	0	0	0	0
1	0	1	0	1	0	1	0	0	0	1	0	0	0
0	0	1	1	0	0	0	0	0	1	0	0	0	0
0	0	0	1	1	1	1	1	1	0	0	0	0	0
1	0	1	1	0	0	0	0	0	1	0	2	2	1
0	0	0	0	0	1	0	0	0	0	0	1	1	2
0	1	0	0	NA	0	1	0	0	NA	NA	0	0	0
0	1	1	0	0	1	0	0	0	NA	0	0	0	0
1	0	1	1	0	1	1	0	1	1	1	1	1	2
0	0	1	0	1	1	1	1	1	0	0	0	0	0
0	0	1	0	0	0	0	0	0	0	0	0	0	0
0	1	1	0	0	1	1	0	1	0	1	0	0	0
0	0	1	0	0	0	1	0	1	NA	1	0	0	0
0	0	1	1	0	0	0	0	0	1	0	0	0	0
0	0	1	1	0	0	0	0	0	1	0	0	0	0
0	0	0	0	1	1	0	0	0	0	0	0	0	0
0	1	1	0	1	0	1	1	1	0	0	0	0	0
0	1	1	0	1	0	1	1	1	0	0	0	0	0
0	1	0	1	0	1	0	0	0	1	0	1	2	2
0	0	1	0	0	0	0	0	0	0	0	0	0	0
0	1	1	1	1	0	1	1	0	1	0	0	0	0
0	0	1	1	1	1	1	1	0	1	NA	1	1	1
0	0	1	0	NA	1	0	0	0	NA	0	0	0	0
1	0	0	1	0	1	0	0	0	1	0	0	0	0
0	1	0	0	1	1	1	0	0	0	1	0	0	0
0	0	1	1	0	1	0	0	0	1	0	0	0	0
1	0	0	0	0	1	0	0	0	0	0	0	0	0
0	0	0	0	0	0	0	NA	NA	0	0	0	0	0
0	0	1	0	1	0	1	1	1	0	0	1	2	1
0	0	0	0	0	0	1	1	1	0	0	0	0	0
0	0	0	0	0	0	1	1	1	0	0	0	0	0
0	0	1	0	0	0	1	1	0	0	0	0	0	0
0	1	1	0	1	1	1	0	1	1	0	0	0	0
0	0	0	0	0	1	0	0	0	0	0	0	0	0
0	1	1	1	0	1	0	0	0	1	0	0	0	0
0	0	1	0	0	1	1	0	1	0	0	0	0	0
0	1	0	0	0	0	0	0	0	0	0	0	0	0
0	0	0	0	1	1	1	0	0	0	0	0	0	0
0	0	0	0	1	1	1	0	1	0	0	0	0	0
0	0	1	1	0	1	0	0	0	1	0	0	0	0
0	0	1	0	0	0	0	0	0	0	0	0	0	0
0	0	1	1	0	0	0	0	0	1	0	0	0	0
1	1	1	0	0	0	1	0	0	NA	0	0	0	0
0	1	0	0	1	1	1	0	0	0	0	0	0	0
0	1	0	0	1	1	1	0	0	0	0	0	0	0
1	0	0	0	1	1	1	0	1	0	1	0	0	0
0	0	0	0	1	1	1	0	1	0	0	0	0	0
1	0	0	0	1	1	1	0	0	0	0	2	1	2
0	0	0	0	1	1	1	1	0	0	0	1	2	1

0	0	1	0	0	1	1	0	0	0	0	0	0	0
0	0	1	0	0	1	1	0	0	0	0	0	0	0
0	0	0	1	1	1	1	0	0	1	0	0	0	0
0	0	1	0	0	0	0	0	0	0	0	0	0	0
0	0	1	0	0	1	0	0	0	0	0	0	0	0
0	0	1	0	0	1	0	0	0	0	0	0	0	0
0	0	1	1	1	1	1	0	1	1	0	0	0	0
0	0	1	0	1	1	1	1	0	0	0	1	1	1
0	0	1	0	1	1	1	1	0	0	0	1	1	1
1	0	1	1	1	1	0	0	0	0	0	0	0	0
1	0	1	1	1	1	0	0	0	0	0	0	0	0
0	0	1	0	0	0	1	1	0	0	0	2	2	1
0	0	1	0	0	0	1	1	0	0	0	2	2	1
0	0	1	0	0	0	1	1	0	0	0	2	2	1
0	0	1	0	0	0	0	0	0	0	0	0	0	0
0	0	1	1	0	1	0	0	0	1	0	0	0	0
0	0	1	1	0	1	0	0	0	1	0	0	0	0
0	0	1	1	0	1	0	0	0	1	0	0	0	0
0	0	1	1	0	1	0	0	0	1	0	0	0	0
1	0	0	0	1	1	0	0	0	0	0	0	0	0
0	0	1	0	0	0	0	0	0	0	0	0	0	0
0	0	1	0	0	0	0	0	0	0	0	0	0	0
0	0	0	0	0	1	0	0	0	0	0	1	2	1
0	0	0	0	0	1	0	0	0	0	0	1	2	1
0	0	0	0	0	1	0	0	0	0	0	0	0	0
0	0	0	0	0	1	0	0	0	0	0	0	0	0
0	0	1	0	0	0	1	0	1	0	0	2	2	1
0	0	1	0	0	0	1	0	1	0	0	2	2	1
0	0	1	0	0	1	0	0	0	0	0	0	0	0
0	0	1	0	0	1	0	0	0	0	0	0	0	0
0	0	1	0	0	1	0	0	0	0	0	0	0	0
0	0	1	0	0	1	1	0	1	0	0	0	0	0
0	0	1	0	0	1	1	0	1	0	0	0	0	0
0	0	1	0	1	1	1	1	1	0	0	0	0	0
1	0	0	1	1	1	0	0	0	1	0	0	0	0
1	1	1	1	0	1	1	1	0	1	0	0	0	0
1	1	1	1	0	1	1	1	0	1	0	0	0	0
0	1	1	1	1	1	1	0	1	1	0	0	0	0
1	1	0	1	0	1	0	0	0	0	0	1	2	2
1	1	0	1	0	1	0	0	0	0	0	1	2	2
0	0	1	1	0	1	1	0	0	1	0	1	1	1
0	0	1	1	0	1	1	0	0	1	0	1	1	1
1	0	1	0	1	1	0	0	0	0	0	2	2	1
1	0	1	0	1	1	0	0	0	0	0	2	2	1
1	0	1	0	1	1	0	0	0	0	0	2	2	1
0	0	0	0	0	1	0	0	0	0	0	0	0	0
0	0	0	0	0	1	0	0	0	0	0	0	0	0
1	0	0	0	1	0	0	0	0	0	0	0	0	0
1	0	0	0	1	0	0	0	0	0	0	0	0	0
1	0	0	0	1	0	0	0	0	0	0	0	0	0
1	0	1	1	0	0	0	0	0	1	0	2	2	1

etformin.eve	probiot	periodontitis	recession	FHPDAC	pack/year	nctype_patie	nasal	CA19	coded_ca19	PDAC_stage
0	0	1	NA	0	3	0	0	18	-1	2
0	0	0	0	0	1	0	0	133	1	4
0	0	0	1	0	2	0	0	126	1	2
0	0	1	1	0	0	0	1	124	1	2
0	0	1	1	0	0	0	1	124	1	2
0	0	0	0	0	0	0	0	5170	1	2
0	0	1	0	0	3	0	0	NA	0	1
0	0	1	0	0	3	0	0	NA	0	1
0	0	NA	0	0	0	0	0	0	-1	2
2	0	0	0	NA	0	0	0	55	1	2
2	0	0	0	NA	0	0	0	55	1	2
0	0	1	1	0	0	0	0	2572	1	2
0	0	1	1	0	3	0	1	151	1	3
1	0	0	0	0	1	0	0	25	-1	2
1	0	0	0	0	1	0	0	25	-1	2
1	0	0	0	0	0	0	0	629	1	4
1	0	0	0	0	0	0	0	629	1	4
0	0	0	0	1	3	2	0	90	1	2
0	0	0	0	0	3	0	0	326	1	2
0	0	1	1	0	2	NA	1	319	1	2
0	0	1	0	0	0	0	0	143	1	2
0	0	0	0	0	0	0	0	0	-1	3
0	0	0	0	0	0	0	0	0	-1	3
1	0	0	0	0	0	0	0	1049	1	2
0	0	1	0	0	3	0	0	2219	1	4
0	0	0	1	0	1	0	0	449	1	4
0	1	1	1	0	0	0	1	1	-1	1
0	0	NA	NA	0	3	0	0	74	1	NA
0	0	0	1	0	0	0	1	NA	0	NA
0	0	0	1	0	0	0	1	NA	0	NA
0	0	0	0	0	0	1	0	1	-1	NA
0	0	1	1	0	0	0	0	4	-1	NA
0	0	1	0	0	2	0	0	1	-1	NA
0	0	0	1	0	3	2	0	NA	0	NA
2	0	NA	0	0	0	0	0	11	-1	NA
2	0	NA	0	0	0	0	0	11	-1	NA
0	0	0	1	0	0	0	0	17	-1	NA
0	0	0	0	1	0	0	0	12	-1	NA
0	0	0	0	0	2	0	0	NA	0	NA
1	0	NA	0	0	2	0	0	NA	0	NA
0	0	0	0	0	3	0	1	NA	0	NA
0	0	0	0	0	3	0	0	8	-1	NA
0	0	0	0	0	0	0	0	5	-1	NA
0	0	0	0	0	1	0	0	9	-1	NA
0	0	0	0	0	2	0	0	NA	0	NA
0	0	0	0	0	0	0	1	NA	0	NA
0	0	0	0	0	0	0	0	15	-1	NA
0	0	0	0	0	0	0	0	6	-1	NA
0	0	0	0	1	3	0	0	NA	0	NA
0	0	1	1	0	3	0	0	6	-1	NA
0	0	NA	0	0	2	0	1	NA	0	NA
0	0	0	0	0	3	0	1	0	-1	NA
1	0	0	0	NA	2	NA	0	NA	0	NA
0	0	0	0	0	3	2	0	NA	0	NA
2	0	0	1	0	3	NA	1	NA	0	NA
0	0	1	0	0	3	1	0	NA	0	NA
0	0	1	1	0	2	2	0	NA	0	NA
0	0	1	1	0	3	0	0	NA	0	NA
0	0	1	0	0	3	NA	0	NA	0	NA
2	0	0	0	0	3	NA	0	NA	0	NA
1	0	1	1	0	2	NA	0	NA	0	NA
1	0	0	0	0	1	NA	0	NA	0	NA
1	0	0	0	0	1	NA	0	NA	0	NA
0	0	0	1	0	2	1	0	NA	0	NA
2	0	1	1	0	3	2	0	NA	0	NA
0	0	1	NA	0	3	1	0	NA	0	NA
0	0	1	NA	0	3	1	0	NA	0	NA
0	0	0	0	0	1	2	0	NA	0	NA
2	0	0	0	0	2	NA	0	NA	0	NA
0	0	1	0	0	0	NA	0	NA	0	NA
1	0	0	1	0	2	NA	0	NA	0	NA
2	0	0	0	0	2	NA	0	NA	0	NA
0	0	1	0	0	3	2	0	NA	0	NA
1	0	0	0	0	2	NA	0	NA	0	NA
0	0	1	1	0	3	0	0	NA	0	NA
0	0	1	1	0	0	0	0	1033	1	2
0	0	1	1	0	0	0	0	1033	1	2
0	1	0	0	0	2	0	1	495	1	2
0	0	NA	1	0	0	0	0	4862	1	4
0	0	NA	1	0	0	0	0	4862	1	4
0	0	0	0	0	1	0	0	NA	0	1
0	0	0	1	0	0	0	1	3537	1	2
1	0	0	1	0	0	0	0	72	1	4
1	0	0	1	0	0	0	0	NA	0	1
0	0	0	0	0	1	0	0	NA	0	2
0	0	0	1	0	0	0	0	760	1	3
1	0	0	1	1	3	0	1	19	-1	1
0	0	0	0	0	1	0	0	NA	0	1

0	0	0	0	0	0	0	0	9810	1	3
0	0	0	0	0	0	0	0	348	1	3
0	0	0	0	0	1	0	0	13617	1	4
0	0	0	1	0	0	0	0	419	1	2
0	0	0	1	0	0	0	0	419	1	2
1	0	0	1	0	0	0	1	34	-1	4
0	0	0	0	0	0	0	0	351	1	3
0	0	0	0	0	1	NA	0	NA	0	3
0	0	0	0	0	0	0	0	455	1	4
0	0	1	0	0	1	0	0	NA	0	2
0	0	1	0	0	1	0	0	NA	0	2
0	0	1	1	1	3	NA	0	36	-1	4
0	0	0	1	NA	0	0	NA	0	-1	3
0	0	0	0	0	0	NA	0	5459	1	4
0	0	0	0	NA	3	0	0	17031	1	3
1	0	0	0	NA	2	0	0	192	1	4
0	0	1	1	NA	0	0	0	489	1	4
0	0	0	NA	NA	0	0	0	1505	1	3
0	0	1	0	NA	0	0	0	26511	1	4
1	0	1	1	NA	1	1	1	208	1	3
NA	0	0	0	NA	1	0	0	NA	0	4
0	0	0	0	0	2	NA	0	NA	0	4
0	0	1	1	0	1	NA	0	NA	0	3
0	0	1	1	0	1	NA	0	98	1	4
0	0	0	0	0	1	NA	0	NA	0	4
1	0	0	0	1	0	NA	0	NA	0	3
0	0	0	1	0	3	NA	0	NA	0	4
0	0	0	0	0	0	0	0	1	-1	NA
0	0	0	1	0	0	0	0	30	-1	NA
0	0	0	0	0	3	0	0	NA	0	NA
0	NA	0	0	0	0	0	0	NA	0	NA
0	0	0	0	0	0	0	0	5	-1	NA
0	0	0	1	0	0	0	1	NA	0	NA
0	1	0	1	0	0	0	0	8	-1	NA
0	0	0	0	0	3	0	0	15	-1	NA
0	0	0	0	0	1	0	1	NA	0	NA
0	0	1	1	0	0	0	0	0	-1	NA
0	0	0	0	0	0	0	0	6	-1	NA
0	1	0	0	0	2	0	0	13	-1	NA
0	0	0	0	0	1	0	0	28	-1	NA
0	0	1	0	0	3	0	1	15	-1	NA
0	0	0	0	0	0	0	0	7	-1	NA
1	0	1	0	0	0	0	0	NA	0	NA
0	0	0	0	0	3	0	0	11	-1	NA
0	0	0	0	0	1	0	0	NA	0	NA
0	0	0	0	0	1	0	0	NA	0	NA
0	0	1	1	0	0	0	0	17	-1	NA
0	0	1	1	0	1	NA	0	4	-1	NA
0	0	1	0	0	1	0	0	0	-1	NA
0	0	0	1	0	0	0	0	6	-1	NA
0	0	0	0	0	0	NA	0	13	-1	NA
NA	0	1	0	0	0	0	0	NA	0	NA
0	0	0	1	0	3	NA	0	8	-1	NA
0	1	1	1	0	1	NA	0	NA	0	NA
0	0	0	0	0	3	NA	0	NA	0	NA
0	0	0	0	1	1	NA	0	0	-1	NA
0	0	1	0	0	0	1	0	NA	0	NA
0	0	0	0	1	1	2	0	NA	0	NA
0	0	0	0	1	0	NA	0	NA	0	NA
0	0	1	0	0	1	2	0	NA	0	NA
0	0	1	1	0	0	2	0	NA	0	NA
1	0	0	0	0	2	2	0	NA	0	NA
0	0	1	NA	0	3	0	0	18	-1	2
0	0	0	0	0	1	0	0	133	1	4
0	0	0	1	0	2	0	0	126	1	2
0	0	1	1	0	0	0	1	124	1	2
0	0	0	0	0	0	0	0	5170	1	2
0	0	1	0	0	3	0	0	NA	0	1
0	0	NA	0	0	0	0	0	0	-1	2
2	0	0	0	NA	0	0	0	55	1	2
0	0	1	1	0	0	0	0	2572	1	2
0	0	1	1	0	3	0	1	151	1	3
1	0	0	0	0	1	0	0	25	-1	2
1	0	0	0	0	0	0	0	629	1	4
0	0	0	0	1	3	2	0	90	1	2
0	0	0	0	0	3	0	0	326	1	2
0	0	1	1	0	2	NA	1	319	1	2
0	0	1	0	0	0	0	0	143	1	2
0	0	0	0	0	0	0	0	0	-1	3
1	0	0	0	0	0	0	0	1049	1	2
0	0	1	0	0	3	0	0	2219	1	4
0	0	0	1	0	1	0	0	449	1	4
0	1	1	1	0	0	0	1	1	-1	1
0	0	NA	NA	0	3	0	0	74	1	NA
0	0	0	1	0	0	0	1	NA	0	NA
0	0	0	0	0	0	1	0	1	-1	NA
0	0	1	1	0	0	0	0	4	-1	NA
0	0	1	0	0	2	0	0	1	-1	NA
0	0	0	1	0	3	2	0	NA	0	NA
2	0	NA	0	0	0	0	0	11	-1	NA

0	0	0	1	0	0	0	0	17	-1	NA
0	0	0	0	1	0	0	0	12	-1	NA
0	0	0	0	0	2	0	0	NA	0	NA
1	0	NA	0	0	2	0	0	NA	0	NA
0	0	0	0	0	3	0	1	NA	0	NA
0	0	0	0	0	3	0	0	8	-1	NA
0	0	0	0	0	0	0	0	5	-1	NA
0	0	0	0	0	1	0	0	9	-1	NA
0	0	0	0	0	2	0	0	NA	0	NA
0	0	0	0	0	0	0	1	NA	0	NA
0	0	0	0	0	0	0	0	15	-1	NA
0	0	0	0	0	0	0	0	6	-1	NA
0	0	0	0	1	3	0	0	NA	0	NA
0	0	1	1	0	3	0	0	6	-1	NA
0	0	NA	0	0	2	0	1	NA	0	NA
0	0	0	0	0	3	0	1	0	-1	NA
1	0	0	0	NA	2	NA	0	NA	0	NA
0	0	0	0	0	3	2	0	NA	0	NA
0	0	1	0	0	3	1	0	NA	0	NA
0	0	1	1	0	3	0	0	NA	0	NA
1	0	0	0	0	1	NA	0	NA	0	NA
2	0	1	1	0	3	2	0	NA	0	NA
0	0	1	NA	0	3	1	0	NA	0	NA
0	0	1	1	0	3	0	0	NA	0	NA
0	0	1	1	0	0	0	0	1033	1	2
0	1	0	0	0	2	0	1	495	1	2
0	0	NA	1	0	0	0	0	4862	1	4
0	0	0	0	0	1	0	0	NA	0	1
0	0	0	1	0	0	0	1	3537	1	2
1	0	0	1	0	0	0	0	72	1	4
1	0	0	0	0	1	0	0	82	1	2
1	0	0	1	0	0	0	0	NA	0	1
0	0	0	0	0	1	0	0	NA	0	2
0	0	0	1	0	0	0	0	760	1	3
1	0	0	1	1	3	0	1	19	-1	1
0	0	0	0	0	1	0	0	NA	0	1
0	0	0	0	0	0	0	0	525	1	1
0	0	0	0	0	0	0	0	9810	1	3
0	0	0	0	0	0	0	0	348	1	3
0	0	0	0	0	1	0	0	13617	1	4
0	0	0	1	0	0	0	0	419	1	2
1	0	0	1	0	0	0	1	34	-1	4
0	0	0	0	0	0	0	0	351	1	3
0	0	0	0	0	0	0	0	455	1	4
0	0	0	0	0	0	0	0	25	-1	4
0	0	1	0	0	1	0	0	NA	0	2
0	0	0	0	0	0	0	0	1	-1	NA
0	1	0	1	0	2	0	0	7	-1	NA
0	0	0	1	0	0	0	0	30	-1	NA
0	0	0	0	0	3	0	0	NA	0	NA
0	NA	0	0	0	0	0	0	NA	0	NA
0	0	0	0	0	0	0	0	5	-1	NA
0	0	0	0	0	0	0	0	7	-1	NA
0	0	1	1	0	0	0	0	NA	0	NA
0	0	1	1	0	0	0	0	NA	0	NA
0	0	0	1	0	0	0	1	NA	0	NA
0	1	0	1	0	0	0	0	8	-1	NA
0	1	0	1	0	0	0	0	8	-1	NA
0	0	0	0	0	3	0	0	15	-1	NA
0	0	0	0	0	1	0	1	NA	0	NA
0	0	1	1	0	0	0	0	0	-1	NA
1	0	0	NA	0	0	0	0	22	-1	NA
0	0	0	0	0	0	0	0	6	-1	NA
0	1	0	0	0	2	0	0	13	-1	NA
0	0	0	0	0	1	0	0	28	-1	NA
0	0	1	0	0	3	0	1	15	-1	NA
0	0	0	0	0	0	0	0	7	-1	NA
0	0	0	0	0	1	NA	0	NA	0	NA
1	0	1	0	0	0	0	0	NA	0	NA
0	0	0	0	0	3	0	0	11	-1	NA
0	0	0	0	0	3	0	0	11	-1	NA
0	0	0	0	0	1	0	0	NA	0	NA
0	0	1	0	0	0	1	0	NA	0	NA
0	0	1	0	0	1	2	0	NA	0	NA
0	0	1	1	0	0	2	0	NA	0	NA
0	0	0	1	0	2	NA	NA	NA	NA	NA
0	0	1	1	0	0	NA	NA	NA	NA	NA
0	0	0	0	0	0	NA	NA	NA	NA	NA
0	0	0	0	0	0	NA	NA	NA	NA	NA
0	0	0	0	1	3	NA	NA	NA	NA	NA
0	0	0	0	0	3	NA	NA	NA	NA	NA
0	0	0	0	0	3	NA	NA	NA	NA	NA
0	0	1	1	0	2	NA	NA	NA	NA	NA
0	0	1	1	0	2	NA	NA	NA	NA	NA
0	0	1	0	0	0	NA	NA	NA	NA	NA
1	0	0	0	0	0	NA	NA	NA	NA	NA
0	1	1	1	0	0	NA	NA	NA	NA	NA
1	0	0	0	0	2	NA	NA	NA	NA	NA
0	0	1	0	0	3	NA	NA	NA	NA	NA
0	0	1	0	0	3	NA	NA	NA	NA	NA

0	0	1	NA	0	3	0	0	18	-1	2
0	0	0	0	0	1	0	0	133	1	4
0	0	0	1	0	2	0	0	126	1	2
0	0	1	1	0	0	0	1	124	1	2
0	0	0	0	0	0	0	0	5170	1	2
0	0	1	0	0	3	0	0	NA	0	1
0	0	NA	0	0	0	0	0	0	-1	2
2	0	0	0	NA	0	0	0	55	1	2
0	0	1	1	0	0	0	0	2572	1	2
0	0	1	1	0	3	0	1	151	1	3
1	0	0	0	0	1	0	0	25	-1	2
1	0	0	0	0	0	0	0	629	1	4
0	0	0	0	1	3	2	0	90	1	2
0	0	0	0	0	3	0	0	326	1	2
0	0	1	1	0	2	NA	1	319	1	2
0	0	1	0	0	0	0	0	143	1	2
0	0	0	0	0	0	0	0	0	-1	3
1	0	0	0	0	0	0	0	1049	1	2
0	0	1	0	0	3	0	0	2219	1	4
0	0	0	1	0	1	0	0	449	1	4
0	1	1	1	0	0	0	1	1	-1	1
0	0	NA	NA	0	3	0	0	74	1	NA
0	0	0	1	0	0	0	1	NA	0	NA
0	0	0	0	0	0	1	0	1	-1	NA
0	0	1	1	0	0	0	0	4	-1	NA
0	0	1	0	0	2	0	0	1	-1	NA
0	0	0	1	0	3	2	0	NA	0	NA
2	0	NA	0	0	0	0	0	11	-1	NA
0	0	0	1	0	0	0	0	17	-1	NA
0	0	0	0	1	0	0	0	12	-1	NA
0	0	0	0	0	2	0	0	NA	0	NA
1	0	NA	0	0	2	0	0	NA	0	NA
0	0	0	0	0	3	0	1	NA	0	NA
0	0	0	0	0	3	0	0	8	-1	NA
0	0	0	0	0	0	0	0	5	-1	NA
0	0	0	0	0	1	0	0	9	-1	NA
0	0	0	0	0	2	0	0	NA	0	NA
0	0	0	0	0	0	0	1	NA	0	NA
0	0	0	0	0	0	0	0	15	-1	NA
0	0	0	0	0	0	0	0	6	-1	NA
0	0	0	0	1	3	0	0	NA	0	NA
0	0	1	1	0	3	0	0	6	-1	NA
0	0	NA	0	0	2	0	1	NA	0	NA
0	0	0	0	0	3	0	1	0	-1	NA
1	0	0	0	NA	2	NA	0	NA	0	NA
0	0	0	0	0	3	2	0	NA	0	NA
2	0	0	1	0	3	NA	1	NA	0	NA
0	0	1	0	0	3	1	0	NA	0	NA
0	0	1	1	0	3	0	0	NA	0	NA
0	0	1	0	0	3	NA	0	NA	0	NA
2	0	0	0	0	3	NA	0	NA	0	NA
1	0	1	1	0	2	NA	0	NA	0	NA
1	0	0	0	0	1	NA	0	NA	0	NA
1	0	0	0	0	1	NA	0	NA	0	NA
0	0	0	1	0	2	1	0	NA	0	NA
2	0	1	1	0	3	2	0	NA	0	NA
0	0	1	NA	0	3	1	0	NA	0	NA
0	0	1	NA	0	3	1	0	NA	0	NA
2	0	0	0	0	2	NA	0	NA	0	NA
0	0	1	0	0	0	NA	0	NA	0	NA
1	0	0	1	0	2	NA	0	NA	0	NA
2	0	0	0	0	2	NA	0	NA	0	NA
1	0	0	0	0	2	NA	0	NA	0	NA
0	0	1	1	0	3	0	0	NA	0	NA
0	0	1	1	0	0	0	0	1033	1	2
0	1	0	0	0	2	0	1	495	1	2
0	0	NA	1	0	0	0	0	4862	1	4
0	0	0	0	0	1	0	0	NA	0	1
0	0	0	1	0	0	0	1	3537	1	2
1	0	0	1	0	0	0	0	72	1	4
1	0	0	1	0	0	0	0	NA	0	1
0	0	0	0	0	1	0	0	NA	0	2
0	0	0	1	0	0	0	0	760	1	3
1	0	0	1	1	3	0	1	19	-1	1
0	0	0	0	0	1	0	0	NA	0	1
0	0	0	0	0	0	0	0	9810	1	3
0	0	0	0	0	0	0	0	348	1	3
0	0	0	0	0	1	0	0	13617	1	4
0	0	0	1	0	0	0	0	419	1	2
1	0	0	1	0	0	0	1	34	-1	4
0	0	0	0	0	0	0	0	351	1	3
0	0	0	0	0	1	NA	0	NA	0	3
0	0	0	0	0	0	0	0	455	1	4
0	0	0	0	0	0	0	0	NA	NA	NA
0	0	0	0	0	0	0	0	NA	NA	NA
0	0	1	0	0	1	0	0	NA	0	2
0	0	1	1	1	3	NA	0	36	-1	4
0	0	0	1	NA	0	0	NA	0	-1	3
0	0	0	0	0	0	NA	0	5459	1	4
0	0	0	0	0	2	NA	0	NA	0	4

0	0	1	1	0	1	NA	0	NA	0	3
0	0	1	1	0	1	NA	0	98	1	4
0	0	0	0	0	1	NA	0	NA	0	4
1	0	0	0	1	0	NA	0	NA	0	3
0	0	0	1	0	3	NA	0	NA	0	4
0	0	0	0	0	0	0	0	1	-1	NA
0	0	0	1	0	0	0	0	30	-1	NA
0	0	0	0	0	3	0	0	NA	0	NA
0	NA	0	0	0	0	0	0	NA	0	NA
0	0	0	0	0	0	0	0	5	-1	NA
0	0	0	1	0	0	0	1	NA	0	NA
0	1	0	1	0	0	0	0	8	-1	NA
0	0	0	0	0	3	0	0	15	-1	NA
0	0	0	0	0	1	0	1	NA	0	NA
0	0	1	1	0	0	0	0	0	-1	NA
0	0	0	0	0	0	0	0	6	-1	NA
0	1	0	0	0	2	0	0	13	-1	NA
0	0	0	0	0	1	0	0	28	-1	NA
0	0	1	0	0	3	0	1	15	-1	NA
0	0	0	0	0	0	0	0	7	-1	NA
1	0	1	0	0	0	0	0	NA	0	NA
0	0	0	0	0	3	0	0	11	-1	NA
0	0	0	0	0	1	0	0	NA	0	NA
0	0	1	1	0	1	NA	0	4	-1	NA
0	0	0	0	0	0	NA	0	13	-1	NA
0	0	0	1	0	3	NA	0	8	-1	NA
0	1	1	1	0	1	NA	0	NA	0	NA
0	0	0	0	0	3	NA	0	NA	0	NA
0	0	0	0	1	1	NA	0	0	-1	NA
0	0	1	0	0	0	1	0	NA	0	NA
0	0	1	0	0	1	2	0	NA	0	NA
0	0	1	1	0	0	2	0	NA	0	NA
0	0	1	NA	0	3	0	0	18	-1	2
0	0	0	0	0	1	0	0	133	1	4
0	0	0	1	0	2	0	0	126	1	2
0	0	1	1	0	0	0	1	124	1	2
0	0	0	0	0	0	0	0	5170	1	2
0	0	1	0	0	3	0	0	NA	0	1
0	0	NA	0	0	0	0	0	0	-1	2
2	0	0	0	NA	0	0	0	55	1	2
0	0	1	1	0	0	0	0	2572	1	2
0	0	1	1	0	3	0	1	151	1	3
1	0	0	0	0	1	0	0	25	-1	2
1	0	0	0	0	0	0	0	629	1	4
0	0	0	0	1	3	2	0	90	1	2
0	0	0	0	0	3	0	0	326	1	2
0	0	1	1	0	2	NA	1	319	1	2
0	0	1	0	0	0	0	0	143	1	2
0	0	0	0	0	0	0	0	0	-1	3
1	0	0	0	0	0	0	0	1049	1	2
0	0	1	0	0	3	0	0	2219	1	4
0	0	0	1	0	1	0	0	449	1	4
0	1	1	1	0	0	0	1	1	-1	1
0	0	NA	NA	0	3	0	0	74	1	NA
0	0	0	1	0	0	0	1	NA	0	NA
0	0	0	0	0	0	1	0	1	-1	NA
0	0	1	1	0	0	0	0	4	-1	NA
0	0	1	0	0	2	0	0	1	-1	NA
0	0	0	1	0	3	2	0	NA	0	NA
2	0	NA	0	0	0	0	0	11	-1	NA
0	0	0	1	0	0	0	0	17	-1	NA
0	0	0	0	1	0	0	0	12	-1	NA
0	0	0	0	0	2	0	0	NA	0	NA
1	0	NA	0	0	2	0	0	NA	0	NA
0	0	0	0	0	3	0	1	NA	0	NA
0	0	0	0	0	3	0	0	8	-1	NA
0	0	0	0	0	0	0	0	5	-1	NA
0	0	0	0	0	1	0	0	9	-1	NA
0	0	0	0	0	2	0	0	NA	0	NA
0	0	0	0	0	0	0	1	NA	0	NA
0	0	0	0	0	0	0	0	15	-1	NA
0	0	0	0	0	0	0	0	6	-1	NA
0	0	0	0	1	3	0	0	NA	0	NA
0	0	1	1	0	3	0	0	6	-1	NA
0	0	NA	0	0	2	0	1	NA	0	NA
0	0	0	0	0	3	0	1	0	-1	NA
1	0	0	0	NA	2	NA	0	NA	0	NA
0	0	0	0	0	3	2	0	NA	0	NA
2	0	0	1	0	NA	NA	1	NA	NA	NA
0	0	1	0	0	3	1	0	NA	0	NA
0	0	1	1	0	2	NA	0	NA	NA	NA
0	0	1	1	0	3	0	0	NA	0	NA
0	0	1	0	0	3	NA	0	NA	NA	NA
2	0	0	0	0	3	NA	0	NA	NA	NA
1	0	1	1	0	2	NA	0	NA	NA	NA
1	0	0	0	0	1	NA	0	NA	0	NA
1	0	0	0	0	1	NA	0	NA	NA	NA
0	0	0	1	0	2	NA	0	NA	NA	NA
2	0	1	1	0	3	2	0	NA	0	NA
0	0	1	NA	0	3	1	0	NA	0	NA

0	0	0	0	0	1	NA	0	NA	NA	NA
2	0	0	0	0	2	NA	0	NA	NA	NA
0	0	1	0	0	0	NA	0	NA	NA	NA
1	0	0	1	0	2	NA	0	NA	NA	NA
2	0	0	0	0	2	NA	0	NA	NA	NA
0	0	1	0	0	3	NA	0	NA	NA	NA
1	0	0	0	0	2	NA	0	NA	NA	NA
0	0	1	1	0	3	0	0	NA	0	NA
0	0	1	1	0	0	0	0	1033	1	2
0	1	0	0	0	2	0	1	495	1	2
0	0	NA	1	0	0	0	0	4862	1	4
0	0	0	0	0	1	0	0	NA	0	1
0	0	0	1	0	0	0	1	3537	1	2
1	0	0	1	0	0	0	0	72	1	4
1	0	0	0	0	1	0	0	82	1	2
1	0	0	1	0	0	0	0	NA	0	1
0	0	0	0	0	1	0	0	NA	0	2
0	0	0	1	0	0	0	0	760	1	3
1	0	0	1	1	3	0	1	19	-1	1
0	0	0	0	0	1	0	0	NA	0	1
0	0	0	0	0	0	0	0	525	1	1
0	0	0	0	0	0	0	0	9810	1	3
0	0	0	0	0	0	0	0	348	1	3
0	0	0	0	0	1	0	0	13617	1	4
0	0	0	1	0	0	0	0	419	1	2
1	0	0	1	0	0	0	1	34	-1	4
0	0	0	0	0	0	0	0	351	1	3
0	0	0	0	0	1	NA	0	NA	NA	NA
0	0	0	0	0	0	0	0	455	1	4
0	0	0	0	0	0	0	0	25	-1	4
0	0	1	0	0	1	0	0	NA	0	2
0	0	1	1	1	3	NA	0	NA	NA	NA
0	0	0	1	0	0	NA	0	NA	NA	NA
0	0	0	0	0	0	NA	0	NA	NA	NA
0	0	0	0	0	3	NA	0	NA	NA	NA
1	0	0	0	1	2	NA	0	NA	NA	NA
0	0	1	1	0	0	NA	0	NA	NA	NA
0	0	0	0	1	0	NA	0	NA	NA	NA
0	0	1	0	0	0	NA	0	NA	NA	NA
1	0	1	1	0	1	NA	1	NA	NA	NA
1	0	0	0	0	1	NA	0	NA	NA	NA
0	0	0	0	0	2	NA	0	NA	NA	NA
0	0	1	1	0	1	NA	0	NA	NA	NA
0	0	1	1	0	1	NA	0	NA	NA	NA
0	0	0	0	0	1	NA	0	NA	NA	NA
1	0	0	0	1	0	NA	0	NA	NA	NA
0	0	0	1	0	3	NA	0	NA	NA	NA
0	0	0	0	0	0	0	0	1	-1	NA
0	1	0	1	0	2	0	0	7	-1	NA
0	0	0	1	0	0	0	0	30	-1	NA
0	0	0	0	0	3	0	0	NA	0	NA
0	NA	0	0	0	0	0	0	NA	0	NA
0	0	0	0	0	0	0	0	5	-1	NA
0	0	0	0	0	0	0	0	7	-1	NA
0	0	1	1	0	0	0	0	NA	0	NA
0	0	1	1	0	0	0	0	NA	0	NA
0	0	0	1	0	0	0	1	NA	0	NA
0	1	0	1	0	0	0	0	8	-1	NA
0	1	0	1	0	0	0	0	8	-1	NA
0	0	0	0	0	3	0	0	15	-1	NA
0	0	0	0	0	1	0	1	NA	0	NA
0	0	1	1	0	0	0	0	0	-1	NA
1	0	0	NA	0	0	0	0	22	-1	NA
0	0	0	0	0	0	0	0	6	-1	NA
0	1	0	0	0	2	0	0	13	-1	NA
0	0	0	0	0	1	0	0	28	-1	NA
0	0	1	0	0	3	0	1	15	-1	NA
0	0	0	0	0	0	0	0	7	-1	NA
0	0	0	0	0	1	NA	0	NA	0	NA
1	0	1	0	0	0	0	0	NA	0	NA
0	0	0	0	0	3	0	0	11	-1	NA
0	0	0	0	0	3	0	0	11	-1	NA
0	0	0	0	0	1	0	0	NA	0	NA
0	0	1	1	0	0	NA	0	NA	NA	NA
0	0	1	1	0	1	NA	0	NA	NA	NA
0	0	1	0	0	1	NA	0	NA	NA	NA
0	0	0	1	0	0	NA	0	NA	NA	NA
0	0	0	0	0	0	NA	0	NA	NA	NA
0	0	1	0	0	0	NA	0	NA	NA	NA
0	0	0	1	0	3	NA	0	NA	NA	NA
0	1	1	1	0	1	NA	0	NA	NA	NA
0	0	0	0	0	3	NA	0	NA	NA	NA
0	0	0	0	1	1	NA	0	NA	NA	NA
0	0	1	0	0	0	1	0	NA	0	NA
0	0	0	0	1	1	NA	0	NA	NA	NA
0	0	0	0	1	1	NA	0	NA	NA	NA
0	0	0	0	1	0	NA	0	NA	NA	NA
0	0	1	0	0	1	2	0	NA	0	NA
0	0	1	1	0	0	2	0	NA	0	NA
1	0	0	0	0	2	NA	0	NA	NA	NA

0	0	1	1	0	3	NA	NA	NA	NA	NA
0	0	1	1	0	3	NA	NA	NA	NA	NA
0	0	0	1	0	2	NA	NA	NA	NA	NA
0	0	1	1	0	0	NA	NA	NA	NA	NA
0	0	0	0	0	0	NA	NA	NA	NA	NA
0	0	0	0	0	0	NA	NA	NA	NA	NA
0	0	0	0	0	0	NA	NA	NA	NA	NA
2	0	0	0	0	0	NA	NA	NA	NA	NA
2	0	0	0	0	0	NA	NA	NA	NA	NA
0	0	1	1	0	0	NA	NA	NA	NA	NA
0	0	1	1	0	0	NA	NA	NA	NA	NA
1	0	0	0	0	1	NA	NA	NA	NA	NA
1	0	0	0	0	1	NA	NA	NA	NA	NA
1	0	0	0	0	1	NA	NA	NA	NA	NA
0	0	0	0	1	3	NA	NA	NA	NA	NA
0	0	0	0	0	3	NA	NA	NA	NA	NA
0	0	0	0	0	3	NA	NA	NA	NA	NA
0	0	0	0	0	3	NA	NA	NA	NA	NA
0	0	0	0	0	3	NA	NA	NA	NA	NA
0	0	1	1	0	2	NA	NA	NA	NA	NA
0	0	1	0	0	0	NA	NA	NA	NA	NA
0	0	1	0	0	0	NA	NA	NA	NA	NA
1	0	0	0	0	0	NA	NA	NA	NA	NA
1	0	0	0	0	0	NA	NA	NA	NA	NA
0	1	1	1	0	0	NA	NA	NA	NA	NA
0	1	1	1	0	0	NA	NA	NA	NA	NA
1	0	0	0	0	2	NA	NA	NA	NA	NA
1	0	0	0	0	2	NA	NA	NA	NA	NA
0	0	1	0	0	3	NA	NA	NA	NA	NA
0	0	1	0	0	3	NA	NA	NA	NA	NA
0	0	1	0	0	3	NA	NA	NA	NA	NA
0	0	1	1	0	3	NA	NA	NA	NA	NA
0	0	1	1	0	3	NA	NA	NA	NA	NA
0	0	1	1	0	3	NA	NA	NA	NA	NA
0	0	1	1	0	0	NA	NA	NA	NA	NA
0	1	0	0	0	2	NA	NA	NA	NA	NA
0	1	0	0	0	2	NA	NA	NA	NA	NA
0	0	1	1	0	0	NA	NA	NA	NA	NA
0	0	0	1	0	0	NA	NA	NA	NA	NA
0	0	0	1	0	0	NA	NA	NA	NA	NA
1	0	0	0	0	1	NA	NA	NA	NA	NA
1	0	0	0	0	1	NA	NA	NA	NA	NA
1	0	0	1	1	3	NA	NA	NA	NA	NA
1	0	0	1	1	3	NA	NA	NA	NA	NA
1	0	0	1	1	3	NA	NA	NA	NA	NA
0	0	0	0	0	0	NA	NA	NA	NA	NA
0	0	0	0	0	0	NA	NA	NA	NA	NA
0	0	0	1	0	0	NA	NA	NA	NA	NA
0	0	0	1	0	0	NA	NA	NA	NA	NA
0	0	0	1	0	0	NA	NA	NA	NA	NA
1	0	0	0	1	0	NA	NA	NA	NA	NA

Supplementary Table S2

Variable Description	Variable Name	QES Column	Full Definition	Categories
Alcohol consumption status	alcohol_status		Ever drunk alcohol regularly, that is at least once per month for 6 months or more	NA = Missing or Don´t know<br>0=Never drinker<br>1=Ever drinker
Alcohol consumption	alc_dd		Drinks/day of total alcohol in lifetime.<br>Weighted average of the alcohol intakes at different ages, with weights equal to the total subject-specific time of investigation<br>Formula: F/(age-20)<br>F= ((mean (beer_st1, beer_s2,..,spirits_st5) * (Min(age_last, age)-20))	**Continuous**<br>**NA = Missing or Don´t know**<br>**0=Never drinker**
Have been diagnosed with diabetes	alldiab	G19A11, G19AIreland11	Subjects report if they have ever been diagnosed with diabetes.	NA=Missing or Don´t Know<br>0= No<br>1=Yes
Have been diagnosed with diabetes categorical by time of diagnosis	diabcat	G19A11, G19B11, agec	Subjects that report having been diagnosed with diabetes are categorized by being diagnosed more or less than 2 years before the time of recruitment in the study	NA=Missing or Don´t Know<br>1 = less than 3years<br>2 = more or equal to 3 years
BMI at two years prior recruitment >=30	obese	A7-1-1 and A61-1	Subjects that have BMI >=30 two years before study recruitment	NA=Missing or Don't know<br>0 = BMI <30<br>1= BMI>=30
Type of pancreatitis	panctype	G20B11	Subjects report if their type of pancreatitis if they have been diagnosed with it.<br>è **<u>Use this variable for analyses</u>**<br>***Pancreatitis cases forced all into the 'chornic' category***	NA=Missing or Don't know<br>0= No pancreatitis<br>1 = Acute<br>2 = Chronic
Type of pancreatitis	Panctype_patient	G20B11	Subjects report if their type of pancreatitis if they have been diagnosed with it. Variable exactly as reported by the patient	**NA=Missing or Don't know**<br>**0= No pancreatitis**<br>**1 = Acute**<br>**2 = Chronic**
Have had asthma	asthma	G5-1-1	Subjects that report ever having asthma.	NA=Missing or Don't know<br>0= No<br>1=Yes
Have had nasal allergies (Rhinitis)	nasal	G11-1-1	Subjects that report having had nasal allergies including hay fever	NA=Missing or Don't know<br>0= No<br>1=Yes
Ever been diagnosed with heartburn	allhburn	G42A11, G42AIreland11	Subjects report if they have ever been diagnosed with Heartburn.	NA=Missing or Don't know<br>0= No<br>1=Yes
Ever been diagnosed with acid regurgitation	allacid	G42B11, G42BIreland11	Subjects report if they have ever been diagnosed with Acid regurgitation	NA=Missing or Don't know<br>0= No<br>1=Yes
Have been diagnosed with Rheumatoid arthritis	allrheum	G2311, G23Ireland11	Subjects report if they have ever been diagnosed with Rheumatoid arthritis	NA=Missing or Don't know<br>0= No<br>1=Yes
Ever been diagnosed with High blood pressure	allhbp	G4011, G40Ireland11	Subjects report if they have ever been diagnosed with High blood pressure	NA=Missing or Don't know<br>0= No<br>1=Yes
Ever been diagnosed with high blood pressure	cholesterol	G4311	Subjects report if they have ever been diagnosed with High cholesterol	NA=Missing or Don't know<br>0= No<br>1=Yes
Have had periodontitis	periodontitis	J7-1-1	Subjects that report, excluding the last two years, to been told by a dentist that they have	NA=Missing or Don't know<br>1=Yes

			periodontitis	0 = No
Have had receding gums	recession	J6-1-1	Subjects report having receding gums	NA=Missing or Don't know<br>1=Yes<br>0 = No
PDAC in a relative	FHPDAC	All K25 variables and all cancer location variables K26	History of PDAC (pancreatic ductal adenocarcinoma) in a relative.<br>Subjects reported if any first degree relative had ever been diagnosed with PDAC.<br>"Yes" was considered if any K25* variables was reported as yes and if location (any k26*variable) was reported to be PDAC.<br>Diagnoses of other cancers, other than PDAC, in first degree relatives was considered as NO FHPDAC	NA=Missing or Don't know<br>0=no FHPDAC<br>1=yes FHPDAC
Diabetes control with diet	diabdiet	cdiabtmodifdieta	Subjects reported if their diabetes is controlled with dietary changes	NA= Missing or Don't know<br>0= Answered No to alldiab<br>1 = Yes<br>2 = No
Diabetes control with insulin	diabin	diabetinsulina	Subjects reported if their diabetes is controlled with insulin pump or injections	NA= Missing or Don't know<br>0= Answered No to alldiab<br>1 = Yes<br>2 = No
Diabetes control with medication	diabmed	diabetmedoral	Subjects reported if their diabetes is controlled with oral medication	NA= Missing or Don't know<br>0= Answered No to alldiab<br>1 = Yes<br>2 = No
Metformin	metformin.ever		Subjects report if ever taking metformin regularly, that is 3 months or more during the last five years	**NA= Don´t know**<br>**0 =No diabetes & no oral medication**<br>**1= Metformin use**<br>**2=No metformin use**
Any acid regurgitation/Heartburn medication	abmedication	tratampirosregur	The subject reports at least one medication of this class (regardless of their report on the disease status)	NA= Missing or Don't know<br>0= No medication<br>1= Medication
Cholesterol medication	cholmedication	g37ttocolestalto	The subject reports at least one medication of this class (regardless of their report on the disease status)	NA= Missing or Don't know<br>0= No medication<br>1= Medication
Aspirin/Paracetamol medication	asparmed	tomadoantiinflamt	Subjects reported if they ever took aspirin or paracetamol or other aintI-inflammatory medication regularly	NA = Don't know<br>0 = No<br>1 = Yes
salicylic	salicylic.ever		Subjects report if they ever took aspirin (irrespective of reportin other anti-inflammatory medication)	NA = Don't know<br>0 = No<br>1 = Yes
paracetamol	paracetamol.ever		Subjects report if they ever took paracetamol (irrespective of reportin other anti-inflammatory medication)	NA = Don't know<br>0 = No<br>1 = Yes
Corticosteroids medication	cortmed	H3-1-1ttocorticoides	Subjects reported if they ever took corticoids regularly	NA = Missing or Don't know<br>0 = No<br>1 = Yes
NSAIDs medication	nsaidmed	H2-1-1	Subjects reported if they ever took nsaids regularly<br>*Answer from Sweden was based on variable H2B_1EU, if nsaids reported then a number 1 was given for this variable	**NA = missing or Don't know**<br>**0 = No**<br>**1 = Yes**
Antibiotics	antibiotic	h3attoantibiot	Subjects reported if they ever took antibiotics during the past five years	**NA = Don't know**<br>**0 = No**<br>**1 = Yes**
Pro/prebiotics	probiot	h4attoprobiotiprebiot	Subjects reported if they ever took **prebiotics or probiotics** regularly during the past five years	**NA = Don't know**<br>**0 = No**<br>**1 = Yes**

Supplementary Table S3: DE Cohort Details

sample_alias	instrument_model	replicate	insert_size	read_length	read_count	ronment_mat	timepoint	gender	age_years	ollection_dat	ct_disease_s
MBJT10257157ST	Illumina HiSeq 4000	0	300	150	47393312	[ENVO:00002	0	female	65	2014-2020	PC
MBJT13029530ST	Illumina HiSeq 4000	0	300	150	44500100	[ENVO:00002	0	male	34	2014-2020	CTR
MBJT13966914ST	Illumina HiSeq 4000	0	300	150	55422086	[ENVO:00002	0	male	66	2014-2020	PC
MBJT14741058ST	Illumina HiSeq 4000	0	300	150	54925804	[ENVO:00002	0	male	29	2014-2020	CTR
MBJT18793872ST	Illumina HiSeq 4000	0	300	150	48908442	[ENVO:00002	0	female	66	2014-2020	PC
MBJT19172978ST	Illumina HiSeq 4000	0	300	150	36491564	[ENVO:00002	0	female	54	2014-2020	CTR
MBJT19739988ST	Illumina HiSeq 4000	0	300	150	43913396	[ENVO:00002	0	male	59	2014-2020	PC
MBJT22022613ST	Illumina HiSeq 4000	0	300	150	37301894	[ENVO:00002	0	male	61	2014-2020	CTR
MBJT22320456ST	Illumina HiSeq 4000	0	300	150	39863688	[ENVO:00002	0	male	74	2014-2020	PC
MBJT23133014ST	Illumina HiSeq 4000	0	300	150	44285414	[ENVO:00002	0	female	40	2014-2020	CTR
MBJT24075712ST	Illumina HiSeq 4000	0	300	150	38908806	[ENVO:00002	0	male	65	2014-2020	PC
MBJT24417068ST	Illumina HiSeq 4000	0	300	150	46462326	[ENVO:00002	0	male	49	2014-2020	CTR
MBJT24770375ST	Illumina HiSeq 4000	0	300	150	41886800	[ENVO:00002	0	female	73	2014-2020	PC
MBJT25397146ST	Illumina HiSeq 4000	0	300	150	38837880	[ENVO:00002	0	female	68	2014-2020	CTR
MBJT26681707ST	Illumina HiSeq 4000	0	300	150	39728784	[ENVO:00002	0	female	57	2014-2020	PC
MBJT28436807ST	Illumina HiSeq 4000	0	300	150	43156950	[ENVO:00002	0	male	63	2014-2020	PC
MBJT28723736ST	Illumina HiSeq 4000	0	300	150	34666406	[ENVO:00002	0	female	79	2014-2020	PC
MBJT30331144ST	Illumina HiSeq 4000	0	300	150	31449568	[ENVO:00002	0	female	76	2014-2020	PC
MBJT32951253ST	Illumina HiSeq 4000	0	300	150	42555236	[ENVO:00002	0	female	78	2014-2020	PC
MBJT34036667ST	Illumina HiSeq 4000	0	300	150	41733736	[ENVO:00002	0	male	55	2014-2020	CTR
MBJT34220088ST	Illumina HiSeq 4000	0	300	150	48977086	[ENVO:00002	0	male	80	2014-2020	PC
MBJT35001732ST	Illumina HiSeq 4000	0	300	150	45479874	[ENVO:00002	0	female	40	2014-2020	CTR
MBJT35016775ST	Illumina HiSeq 4000	0	300	150	47763580	[ENVO:00002	0	female	81	2014-2020	PC
MBJT35058600ST	Illumina HiSeq 4000	0	300	150	51795444	[ENVO:00002	0	female	71	2014-2020	PC
MBJT40306752ST	Illumina HiSeq 4000	0	300	150	46477424	[ENVO:00002	0	female	24	2014-2020	CTR
MBJT40435704ST	Illumina HiSeq 4000	0	300	150	45919556	[ENVO:00002	0	male	63	2014-2020	PC
MBJT40701160ST	Illumina HiSeq 4000	0	300	150	50045710	[ENVO:00002	0	female	26	2014-2020	CTR
MBJT40707177ST	Illumina HiSeq 4000	0	300	150	40242120	[ENVO:00002	0	female	82	2014-2020	PC
MBJT41036209ST	Illumina HiSeq 4000	0	300	150	42245120	[ENVO:00002	0	male	67	2014-2020	PC
MBJT41854002ST	Illumina HiSeq 4000	0	300	150	40271702	[ENVO:00002	0	male	46	2014-2020	CTR
MBJT42733650ST	Illumina HiSeq 4000	0	300	150	35444906	[ENVO:00002	0	male	80	2014-2020	PC
MBJT42917972ST	Illumina HiSeq 4000	0	300	150	40122088	[ENVO:00002	0	female	72	2014-2020	CTR
MBJT44556531ST	Illumina HiSeq 4000	0	300	150	53434320	[ENVO:00002	0	female	50	2014-2020	PC
MBJT46290466ST	Illumina HiSeq 4000	0	300	150	37640290	[ENVO:00002	0	female	49	2014-2020	CTR
MBJT47281689ST	Illumina HiSeq 4000	0	300	150	55830262	[ENVO:00002	0	female	76	2014-2020	PC
MBJT47376024ST	Illumina HiSeq 4000	0	300	150	38754652	[ENVO:00002	0	female	66	2014-2020	PC
MBJT47759212ST	Illumina HiSeq 4000	0	300	150	31813116	[ENVO:00002	0	male	70	2014-2020	PC
MBJT47974019ST	Illumina HiSeq 4000	0	300	150	38234074	[ENVO:00002	0	female	81	2014-2020	PC
MBJT54042978ST	Illumina HiSeq 4000	0	300	150	51374756	[ENVO:00002	0	female	50	2014-2020	CTR
MBJT54598736ST	Illumina HiSeq 4000	0	300	150	46583366	[ENVO:00002	0	male	28	2014-2020	CTR
MBJT54773511ST	Illumina HiSeq 4000	0	300	150	45819402	[ENVO:00002	0	male	31	2014-2020	CTR
MBJT55041082ST	Illumina HiSeq 4000	0	300	150	25828972	[ENVO:00002	0	male	80	2014-2020	PC
MBJT55763017ST	Illumina HiSeq 4000	0	300	150	37164202	[ENVO:00002	0	female	73	2014-2020	PC
MBJT56486370ST	Illumina HiSeq 4000	0	300	150	60275548	[ENVO:00002	0	male	87	2014-2020	CTR
MBJT57869938ST	Illumina HiSeq 4000	0	300	150	36431696	[ENVO:00002	0	female	61	2014-2020	PC
MBJT58818096ST	Illumina HiSeq 4000	0	300	150	42617446	[ENVO:00002	0	female	69	2014-2020	PC
MBJT59111766ST	Illumina HiSeq 4000	0	300	150	39629386	[ENVO:00002	0	female	55	2014-2020	CTR
MBJT61614909ST	Illumina HiSeq 4000	0	300	150	32278494	[ENVO:00002	0	female	56	2014-2020	PC
MBJT61989978ST	Illumina HiSeq 4000	0	300	150	44899094	[ENVO:00002	0	male	67	2014-2020	CTR
MBJT63497322ST	Illumina HiSeq 4000	0	300	150	41550048	[ENVO:00002	0	female	65	2014-2020	PC
MBJT64178099ST	Illumina HiSeq 4000	0	300	150	45792268	[ENVO:00002	0	female	28	2014-2020	CTR
MBJT64323881ST	Illumina HiSeq 4000	0	300	150	51879638	[ENVO:00002	0	female	87	2014-2020	PC
MBJT65314632ST	Illumina HiSeq 4000	0	300	150	40100360	[ENVO:00002	0	male	77	2014-2020	PC
MBJT66677231ST	Illumina HiSeq 4000	0	300	150	38271172	[ENVO:00002	0	male	59	2014-2020	CTR
MBJT69789353ST	Illumina HiSeq 4000	0	300	150	37463630	[ENVO:00002	0	female	79	2014-2020	PC
MBJT71206045ST	Illumina HiSeq 4000	0	300	150	37446348	[ENVO:00002	0	male	64	2014-2020	PC
MBJT73212715ST	Illumina HiSeq 4000	0	300	150	29746146	[ENVO:00002	0	male	81	2014-2020	PC
MBJT73621698ST	Illumina HiSeq 4000	0	300	150	46013560	[ENVO:00002	0	male	22	2014-2020	CTR
MBJT75354344ST	Illumina HiSeq 4000	0	300	150	42836122	[ENVO:00002	0	male	84	2014-2020	PC
MBJT75856433ST	Illumina HiSeq 4000	0	300	150	38763576	[ENVO:00002	0	female	60	2014-2020	PC
MBJT76001019ST	Illumina HiSeq 4000	0	300	150	41432142	[ENVO:00002	0	female	36	2014-2020	CTR
MBJT77767153ST	Illumina HiSeq 4000	0	300	150	51551804	[ENVO:00002	0	female	70	2014-2020	CTR
MBJT79123647ST	Illumina HiSeq 4000	0	300	150	40277574	[ENVO:00002	0	female	72	2014-2020	PC
MBJT82870648ST	Illumina HiSeq 4000	0	300	150	47315674	[ENVO:00002	0	male	30	2014-2020	CTR
MBJT83133832ST	Illumina HiSeq 4000	0	300	150	40995200	[ENVO:00002	0	male	67	2014-2020	PC
MBJT83294902ST	Illumina HiSeq 4000	0	300	150	37680998	[ENVO:00002	0	male	64	2014-2020	CTR
MBJT85450199ST	Illumina HiSeq 4000	0	300	150	47792460	[ENVO:00002	0	male	76	2014-2020	PC
MBJT85948313ST	Illumina HiSeq 4000	0	300	150	44611384	[ENVO:00002	0	male	49	2014-2020	CTR
MBJT87587928ST	Illumina HiSeq 4000	0	300	150	43175984	[ENVO:00002	0	female	62	2014-2020	CTR
MBJT89319187ST	Illumina HiSeq 4000	0	300	150	43302384	[ENVO:00002	0	female	40	2014-2020	PC
MBJT90536787ST	Illumina HiSeq 4000	0	300	150	44711476	[ENVO:00002	0	female	64	2014-2020	CTR
MBJT92561737ST	Illumina HiSeq 4000	0	300	150	40739728	[ENVO:00002	0	female	32	2014-2020	CTR
MBJT92636570ST	Illumina HiSeq 4000	0	300	150	36248620	[ENVO:00002	0	male	57	2014-2020	PC
MBJT93071877ST	Illumina HiSeq 4000	0	300	150	45583612	[ENVO:00002	0	female	56	2014-2020	CTR
MBJT94530819ST	Illumina HiSeq 4000	0	300	150	44454590	[ENVO:00002	0	female	84	2014-2020	PC
MBJT97792954ST	Illumina HiSeq 4000	0	300	150	42824760	[ENVO:00002	0	male	68	2014-2020	PC

bmi	smoker	medication	stage	stage_detaile	etastasis (he	Treatment	y_of_operati	erapy_after_G	_of_last_foll	date_of follo	Ca_19_9
27	smoker	NA	2	2b	0	1	27.02.20	1	0	May 20	1702
NA	NA	no	NA	NA	NA	NA	NA	NA	NA	NA	NA
23,8	non-smoker	NA	2	2b	0	1	21.02.20	1	0	02.06.20	2
NA	NA	no	NA	NA	NA	NA	NA	NA	NA	NA	NA
29,7	non-smoker	NA	2	2a	0	4	31.01.20	3	0	03.06.20	1172
NA	NA	L-Thyrox	NA	NA	NA	NA	NA	NA	NA	NA	NA
21,47	non-smoker	NA	4	4	0	6	NA	3	NA	NA	1066
27,78	smoker	NA	NA	NA	0	1	01.04.19	NA	NA	NA	1575
26,01	non-smoker	NA	1	1b	0	1	08.01.19	2	0	19.06.20	17081
NA	NA	no	NA	NA	NA	NA	NA	NA	NA	NA	NA
29,54	smoker	NA	2	2b	0	1	04.07.19	2	0	06.03.20	2
NA	NA	no	NA	NA	NA	NA	NA	NA	NA	NA	NA
28,2	smoker	NA	4	4	NA	8	NA	0	0	16.09.20	4
20,2	non-smoker	NA	NA	NA	0	7	09.07.19	2	0	08.05.20	1412
39,68	smoker	NA	3	3	0	4	08.01.19	3	1	08.11.19	1000
30,37	smoker	NA	3	3	0	2	26.01.19	2	0	15.06.20	71091
22,1	non-smoker	NA	4	4	0	1	04.11.19	1	0	10.08.20	5097
32,8	non-smoker	NA	4	4	0	1	10.02.20	0	0	23.04.20	2183
22,68	non-smoker	NA	4	4	1	4	06.05.19	4	1	17.06.20	2
NA	NA	no	NA	NA	NA	NA	NA	NA	NA	NA	NA
32,3	non-smoker	NA	2	2a	0	1	20.08.19	2	0	14.07.20	164583
NA	NA	no	NA	NA	NA	NA	NA	NA	NA	NA	NA
19,7	smoker	NA	3	3	0	1	03.03.20	1	0	30.03.20	2346
29,76	smoker	NA	4	4	1	4	04.02.19	3	1	17.03.20	3447
NA	NA	no	NA	NA	NA	NA	NA	NA	NA	NA	NA
27,1	non-smoker	NA	2	2b	1	3	NA	2, 3	1	04.05.20	379
NA	NA	no	NA	NA	NA	NA	NA	NA	NA	NA	NA
24,22	smoker	NA	4	4	0	8	NA	NA	0	16.09.20	138
23,26	non-smoker	NA	4	4	0	1	30.07.19	0	0	10.09.20	906
NA	smoker	no	NA	NA	NA	NA	NA	NA	NA	NA	NA
26,8	non-smoker	NA	2	2b	0	3	16.04.19	0	1	13.07.19	160969
31,24	smoker	NA	NA	NA	0	1	17.04.19	NA	NA	NA	834
18,73	non-smoker	NA	3	3	0	1, 7	30.09.19	5	0	unknown	2
NA	NA	L-Thyrox	NA	NA	NA	NA	NA	NA	NA	NA	NA
22,7	non-smoker	NA	3	3	0	2	04.02.20	2	0	04.06.20	6021
22,39	non-smoker	NA	4	4	0	5	03.06.19	0	1	03.07.19	118805
27,13	non-smoker	NA	1	1a	0	1, 3	1.2016-01.07.	0	0	19.07.20	4
19,38	smoker	NA	3	3	NA	1	23.01.20	3	NA	16.09.20	985
28,9	non-smoker	NA	NA	NA	0	4	14.11.19	NA	NA	NA	76814
NA	NA	no	NA	NA	NA	NA	NA	NA	NA	NA	NA
NA	NA	no	NA	NA	NA	NA	NA	NA	NA	NA	NA
33,03	non-smoker	NA	2	2a	0	3	02.08.19	2	1	Jan 20	611609
25,64	non-smoker	NA	4	4	1	4	30.01.19	3	1	09.04.19	42
21,5	non-smoker	NA	NA	NA	1	2	18.02.20	2	0	11.09.20	239
32,87	non-smoker	NA	2	2a	0	1	12.04.19	2	0	20.05.20	19635
25,35	non-smoker	NA	3	3	0	1	14.02.19	2	1	24.01.20	130723
NA	NA	no	NA	NA	NA	NA	NA	NA	NA	NA	NA
23,92	smoker	NA	2	2b	0	1	20.03.19	2	0	08.06.20	27812
27,47	non-smoker	NA	NA	NA	0	1	13.02.19	NA	NA	NA	16658
30,4	smoker	NA	1	1b	0	3	10.07.19	NA	NA	NA	222
NA	NA	no	NA	NA	NA	NA	NA	NA	NA	NA	NA
24,14	non-smoker	NA	2	2b	0	1	29.01.19	0	1	30.01.19	7009
28,73	non-smoker	NA	2	2a	0	2	24.01.19	0	1	29.03.19	7212
NA	NA	no	NA	NA	NA	NA	NA	NA	NA	NA	NA
27,8	non-smoker	NA	4	4	0	1	20.12.19	2	0	06.08.20	87632
NA	smoker	NA	NA	NA	0	NA	NA	4	NA	NA	12779
26,9	non-smoker	NA	3	3	0	3	20.02.20	NA	NA	NA	3464
NA	NA	no	NA	NA	NA	NA	NA	NA	NA	NA	NA
28,04	non-smoker	NA	1	1b	NA	8	NA	0	0	16.09.20	379
25,9	non-smoker	NA	4	4	1	4	16.01.19	0	1	07.03.19	978
NA	NA	no	NA	NA	NA	NA	NA	NA	NA	NA	NA
25,83	non-smoker	NA	NA	NA	0	2	13.03.19	NA	NA	NA	1865
29,76	non-smoker	NA	3	3	0	3	02.05.19	2	0	12.05.20	2498
NA	NA	no	NA	NA	NA	NA	NA	NA	NA	NA	NA
22,58	non-smoker	NA	3	3	1	7	01.10.14	2	0	16.09.20	451
NA	NA	no	NA	NA	NA	NA	NA	NA	NA	NA	NA
24,61	non-smoker	NA	3	3	0	3	18.07.19	NA	NA	NA	55435
NA	NA	no	NA	NA	NA	NA	NA	NA	NA	NA	NA
26,29	non-smoker	NA	NA	NA	0	6	NA	NA	0	18.03.20	380838
24,22	smoker	NA	3	3	0	2	21.02.19	2	0	04.05.20	140631
NA	NA	no	NA	NA	NA	NA	NA	NA	NA	NA	NA
NA	NA	no	NA	NA	NA	NA	NA	NA	NA	NA	NA
22,9	non-smoker	NA	3	3	0	1	19.12.19	2	0	Aug 20	1146
NA	NA	L-Thyrox	NA	NA	NA	NA	NA	NA	NA	NA	NA
19,92	non-smoker	NA	3	3	1	1	06.09.18	2	1	21.08.19	NA
23,84	non-smoker	NA	2	2a	NA	8	NA	3	1	.04.2020 and	142

alcohol	lcohol_amour	medication.1	ntibiotics <28	PPI	recent_medic	lities_other_t	rbidities_dial	coded_ca19	talogical_sta	ampling_time
1	3-4 per week	1	0	1	NA	1	0	1	cN1L0V0Pn1G	ay before operation
NA	NA	NA	NA	NA	NA	NA	NA	0	NA	NA
0	NA	1	0	1	NA	1	0	-1	N0L0V0Pn1G	ay before operation
NA	NA	NA	NA	NA	NA	NA	NA	0	NA	NA
1	1 per week	1	0	1	NA	1	0	1	N0L0V0Pn1G	ay before operation
NA	NA	NA	NA	NA	NA	NA	NA	0	NA	NA
0	-	1	0	0	7	2	0	1	IV	single sample
1	daily	1	0	1	NA	1	0	-1	NA	ay before operation
1	ex abusus	1	1	0	NA	1	1	1	2N0LV0Pn1G	ay before operation
NA	NA	NA	NA	NA	NA	NA	NA	0	NA	NA
0	NA	1	0	0	NA	1	1	-1	L0V0Pn1G3R	ay before operation
NA	NA	NA	NA	NA	NA	NA	NA	0	NA	NA
0	-	1	1	1	1, 3, 4, 5, 7	1, 4	0	-1	IV	single sample
1	3-4 per week	1	0	0	NA	1	0	1	NA	ay before operation
0	-	1	0	1	2, 3, 4, 5, 7	1	0	1	III	single sample
1	daily	1	0	0	NA	0	1	1	N2L0V0Pn1G	ay before operation
1	1-2 per week	1	1	1	NA	1	0	1	NA	ay before operation
0	NA	1	0	1	NA	1	0	-1	l1(hep)L0V0P	ay before operation
1	1-2 per week	1	0	0	NA	3	0	-1	NA	ay before operation
NA	NA	NA	NA	NA	NA	NA	NA	0	NA	NA
0	NA	1	1	1	NA	1	0	1	aG2; pT3bN0L	ay before operation
NA	NA	NA	NA	NA	NA	NA	NA	0	NA	NA
1	1 per week	1	0	0	NA	1	0	1	N2L0V1Pn1G	ay before operation
0	NA	1	1	1	NA	2	0	-1	NA	ay before operation
NA	NA	NA	NA	NA	NA	NA	NA	0	NA	NA
1	occasionally	1	0	1	6, 7	1	0	1	pN1 (2/18), L	single sample
NA	NA	NA	NA	NA	NA	NA	NA	0	NA	NA
0	-	1	1	1	NA	1	0	1	IV	single sample
1	daily	1	1	1	NA	1	0	-1	M1(lym)L0V0P	ay before operation
NA	NA	NA	NA	NA	NA	NA	NA	0	NA	NA
0	NA	1	0	0	NA	1	0	1	N1L0V0Pn1G	ay before operation
1	daily	0	0	0	NA	0	0	-1	NA	ay before operation
1	onsumption sir	1	0	1	2, 5, 6, 7	1	1	-1	III	single sample
NA	NA	NA	NA	NA	NA	NA	NA	0	NA	NA
0	NA	1	0	0	NA	1	0	1	N2L0V0Pn1G	ay before operation
0	NA	1	0	0	NA	1, 4	1	1	NA	uvant radiochemotherapy
0	-	1	0	1	2, 4, 5, 7	6	1	-1	IA	single sample
0	-	1	1	0	2, 6, 2007	6	0	1	III	single sample
0	NA	1	0	0	NA	1, 3	1	1	NA	ay before operation
NA	NA	NA	NA	NA	NA	NA	NA	0	NA	NA
NA	NA	NA	NA	NA	NA	NA	NA	0	NA	NA
0	NA	1	1	1	NA	1	1	1	N0L0V0Pn1G	ay before operation
0	NA	1	0	1	NA	1	1	1	NA	ay before operation
0	NA	1	0	0	NA	1	0	-1	NA	ay before operation
0	NA	1	0	1	NA	1	1	1	N0L0V0Pn1G	ay before operation
0	NA	0	0	0	NA	0	0	1	N2L0V0Pn1G	ay before operation
NA	NA	NA	NA	NA	NA	NA	NA	0	NA	NA
1	daily	1	0	0	NA	1	0	1	N1L0V0Pn1G	ay before operation
1	daily	1	1	1	NA	1	1	1	NA	ay before operation
0	NA	1	0	0	NA	1	0	-1	N0L0V0Pn0G	ay before operation
NA	NA	NA	NA	NA	NA	NA	NA	0	NA	NA
1	1 per week	1	0	0	NA	1	0	1	N1L0V0Pn1G	ay before operation
0	NA	1	0	1	NA	1	0	1	N0L0V1Pn1G	ay before operation
NA	NA	NA	NA	NA	NA	NA	NA	0	NA	NA
0	NA	1	0	0	NA	1	0	1	NA	ay before operation
1	daily	1	0	1	NA	3	0	1	NA	djuvant chemotherapy
0	NA	1	0	0	NA	1	0	-1	N2L1V0Pn1G	ay before operation
NA	NA	NA	NA	NA	NA	NA	NA	0	NA	NA
0	-	1	1	0	1, 2, 3, 5, 7	1	1	1	Ib	single sample
1	1 per week	0	1	0	NA	0	0	-1	NA	ay before operation
NA	NA	NA	NA	NA	NA	NA	NA	0	NA	NA
1	1 per month	1	0	0	NA	2	0	-1	NA	ay before operation
0	NA	1	0	0	NA	1	0	1	N1L0V0Pn1G	ay before operation
NA	NA	NA	NA	NA	NA	NA	NA	0	NA	NA
1	lcohol since 2	1	0	1	2, 5, 2007	5	1	1	1 (1/12), L1, V	single sample
NA	NA	NA	NA	NA	NA	NA	NA	0	NA	NA
1	1 per month	1	0	0	NA	1	0	1	N2L1V0Pn1G	ay before operation
NA	NA	NA	NA	NA	NA	NA	NA	0	NA	NA
0	NA	1	0	1	NA	1	0	1	NA	ay before operation
0	NA	1	0	0	NA	5	0	1	N2L0V1Pn1G	ay before operation
NA	NA	NA	NA	NA	NA	NA	NA	0	NA	NA
NA	NA	NA	NA	NA	NA	NA	NA	0	NA	NA
1	daily	1	1	0	NA	1	0	1	N2L1V0Pn1G	ay before operation
NA	NA	NA	NA	NA	NA	NA	NA	0	NA	NA
0	-	1	0	1	1, 7	1, 6	0	-1	III	single sample
1	day in the last	1	0	1	1, 7	1	1	1	IIA	single sample

Supplementary Table S4

	Stool			Saliva		
**Meta variable**	**Sum sq**	**p value**	**adj p**	**Sum sq**	**p value**	**adj p**
abmedication	7,40E+08	0.434882370	1.0	7,26E+07	0.726039062	1.0
Age	5,57E+10	0.348999552	1.0	1,43E+10	0.992133250	1.0
Alcohol consumption	3,28E+09	0.105017752	1.0	4,22E+07	0.796587916	1.0
Acid regurgitation	1,92E+09	0.215874045	1.0	2,05E+07	0.857472013	1.0
Diabetes	1,47E+07	0.914256854	1.0	4,78E+08	0.387311299	1.0
Heart burn	7,20E+08	0.445550072	1.0	2,11E+09	0.059764110	1.0
High blood pressure	8,65E+06	0.934099012	1.0	4,70E+06	0.931832163	1.0
Rheumatoid arthritis	7,36E+08	0.444113445	1.0	3,28E+07	0.821293457	1.0
Antibiotic	6,18E+07	0.825159237	1.0	3,55E+08	0.455050912	1.0
Aspirin/paracetamol	1,36E+09	0.297843618	1.0	1,12E+09	0.181428572	1.0
Asthma	1,76E+09	0.236597169	1.0	4,57E+08	0.395506158	1.0
Center	4,90E+09	0.046953480	1.0	2,76E+06	0.947458361	1.0
Cholesterol	1,72E+09	0.246154381	1.0	2,93E+08	0.493014836	1.0
Cholmedication	6,33E+05	0.982226159	1.0	1,48E+08	0.663216095	1.0
Corticosteroids	2,24E+09	0.179684582	1.0	4,11E+08	0.406201881	1.0
cpy1	7,16E+10	0.825078663	1.0	2,39E+10	0.800141666	1.0
Bilirirubin direct	1,32E+11	0.543089087	1.0	5,08E+10	0.857634747	1.0
Bilirubin lab	1,30E+11	0.455465155	1.0	4,85E+10	0.802784617	1.0
FHPDAC	2,40E+06	0.964420558	1.0	4,78E+08	0.383616587	1.0
Gender	1,04E+09	0.362627665	1.0	5,98E+08	0.330808453	1.0
Jaundice	9,45E+09	0.004406831	0.3437328	4,30E+08	0.412200163	1.0
Jaundice imputed	8,32E+09	0.009192609	0.7078309	4,41E+08	0.403644362	1.0
Library_size	8,15E+09	0.086373435	1.0	1,55E+09	0.487294778	1.0
Metformin usage	3,98E+09	0.205930855	1.0	3,12E+08	0.783170829	1.0
Obesity	8,37E+08	0.415414037	1.0	3,49E+08	0.450252695	1.0
Paracetamol	4,49E+09	0.058802839	1.0	2,03E+09	0.072918539	1.0
Periodontitis	1,73E+08	0.710712567	1.0	1,31E+09	0.136666489	1.0
Probiotic	1,11E+07	0.925379621	1.0	1,09E+07	0.894062783	1.0
Receding gums	8,17E+08	0.415025012	1.0	2,69E+08	0.508708572	1.0
Salicylic acid	1,85E+09	0.227061461	1.0	1,94E+08	0.582723500	1.0
Salicylic acid	1,85E+09	0.227061461	1.0	1,94E+08	0.582723500	1.0
Smoking	9,62E+07	0.782721018	1.0	2,24E+09	0.059311754	1.0
Direct Bilirubin	2,75E+09	0.138132461	1.0	1,71E+09	0.102796407	1.0
Total Bilirubin	7,13E+08	0.440177205	1.0	2,87E+09	0.031822359	1.0

Supplementary Table S5

	Stool		Saliva	
**Meta variable**	**R2 Bray-Curtis**	**p value**	**R2 Bray-Curtis**	**p value**
status	0.023120267	0.0001	0.010468749	0.6507
center	0.011138008	0.2069	0.016751291	0.1285
age	0.013765355	0.0320	0.018667727	0.0680
gender	0.010546251	0.2884	0.017940759	0.0884
jaundice_imp	0.015980165	0.0087	0.007371362	0.9560
diabetes	0.013610607	0.0396	0.008990599	0.8264
obesity	0.008734297	0.6915	0.016455787	0.1449
smoking	0.008943945	0.6466	0.015490596	0.1926
alcohol_con	0.009730450	0.4545	0.017867998	0.0929
periodontitis	0.009018863	0.6237	0.009449706	0.7709
cholesterol	0.007848892	0.8750	0.010637989	0.6368
metformin	0.008315828	0.7739	0.018288711	0.0898
salicylic	0.009192529	0.5749	0.008768629	0.8462
antibiotic	0.007858670	0.8685	0.006825246	0.9740
aspirin/paracetamol	0.012544261	0.0847	0.020375695	0.0432
corticosteroids	0.010196054	0.3492	0.017140805	0.1277
asthma	0.008577892	0.7244	0.014670641	0.2412
acid regurgitation	0.011797158	0.1335	0.006447749	0.9801
rheumatoid arthritis	0.008798014	0.6747	0.011646596	0.5123
probiotic	0.009681840	0.4578	0.011088990	0.5759
paracetamol	0.010198223	0.3573	0.009887242	0.7242
heartburn	0.008810470	0.6760	0.013602585	0.3241
high blood pressure	0.007920655	0.8614	0.010496978	0.6491
receding gums	0.008765518	0.6873	0.007806693	0.9229
FHPDAC	0.010506425	0.2887	0.012944276	0.3752
acid med	0.010032433	0.3870	0.007517951	0.9390

Supplementary Table S6

Study	Diagnosis	Cohort siz	Pred.pos	Pred.neg	FPR	Model	Accession number
Average BRCA	BRCA	62			0,35	Model 1	
Average BRCA	BRCA	62			0,05	Model 2	
Average CD	CD	340			0,35	Model 1	
Average CD	CD	340			0,05	Model 2	
Average CRC	CRC	679			0,25	Model 1	
Average CRC	CRC	679			0,04	Model 2	
Average CTR	CTR	3872			0,17	Model 1	
Average CTR	CTR	3872			0,01	Model 2	
Average LD	LD	237			0,26	Model 1	
Average LD	LD	237			0,07	Model 2	
Average T1D	T1D	87			0,29	Model 1	
Average T1D	T1D	87			0,00	Model 2	
Average T2D	T2D	382			0,23	Model 1	
Average T2D	T2D	382			0,02	Model 2	
Average UC	UC	352			0,18	Model 1	
Average UC	UC	352			0,01	Model 2	
Buschart 2016	CTR	26	5	21	0,19	Model 1	PRJNA289586
Buschart 2016	T1D	27	8	19	0,30	Model 1	
Buschart 2016	CTR	26	0	26	0,00	Model 2	
Buschart 2016	T1D	27	0	27	0,00	Model 2	
Dhakan 2019	CTR	110	19	91	0,17	Model 1	PRJNA397112
Dhakan 2019	CTR	110	2	108	0,02	Model 2	
Feng 2015	CTR	63	7	56	0,11	Model 1	PRJEB7774
Feng 2015	CRC	93	11	82	0,12	Model 1	
Feng 2015	CTR	63	2	61	0,03	Model 2	
Feng 2015	CRC	93	2	91	0,02	Model 2	
FijiCOMP	CTR	172	14	158	0,08	Model 1	PRJNA217052
FijiCOMP	CTR	172	1	171	0,01	Model 2	
FMT Vaughn	CD	15	4	11	0,27	Model 1	PRJNA321058
FMT Vaughn	CD	15	1	14	0,07	Model 2	
Franzosa 2018	CTR	56	5	51	0,09	Model 1	PRJNA400072
Franzosa 2018	UC	76	10	66	0,13	Model 1	
Franzosa 2018	CD	88	19	69	0,22	Model 1	
Franzosa 2018	CTR	56	0	56	0,00	Model 2	
Franzosa 2018	UC	76	0	76	0,00	Model 2	
Franzosa 2018	CD	88	2	86	0,02	Model 2	
He 2017	CTR	54	11	43	0,20	Model 1	PRJEB15371
He 2017	CD	49	33	16	0,67	Model 1	
He 2017	CTR	54	0	54	0,00	Model 2	
He 2017	CD	49	5	44	0,10	Model 2	
Price-Lloyd 2019	UC	84	26	58	0,31	Model 1	PRJNA398089
Price-Lloyd 2019	CD	151	38	113	0,25	Model 1	
Price-Lloyd 2019	CTR	65	5	60	0,08	Model 1	
Price-Lloyd 2019	UC	84	1	83	0,01	Model 2	
Price-Lloyd 2019	CD	151	2	149	0,01	Model 2	
Price-Lloyd 2019	CTR	65	0	65	0,00	Model 2	

Hoyles 2018	LD	73	3	70	0,04	Model 1	PRJEB14215
Hoyles 2018	LD	73	3	70	0,04	Model 2	
Karlsson 2013	CTR	43	8	35	0,19	Model 1	PRJEB1786
Karlsson 2013	T2D	102	14	88	0,14	Model 1	
Karlsson 2013	CTR	43	0	43	0,00	Model 2	
Karlsson 2013	T2D	102	0	102	0,00	Model 2	
Kuang 2019	CTR	59	18	41	0,31	Model 1	PRJEB18755
Kuang 2019	T1D	29	9	20	0,31	Model 1	
Kuang 2019	CTR	59	0	59	0,00	Model 2	
Kuang 2019	T1D	29	0	29	0,00	Model 2	
Liu 2016	CTR	110	5	105	0,05	Model 1	PRJNA328899
Liu 2016	CTR	110	0	110	0,00	Model 2	
Mardinoglu 2018	LD	48	4	44	0,08	Model 1	PRJNA420817
Mardinoglu 2018	LD	48	0	48	0,00	Model 2	
Forslund 2015	CTR	372	85	287	0,23	Model 1	224, PRJEB1220, PRJ
Forslund 2015	T2D	78	16	62	0,21	Model 1	
Forslund 2015	T1D	31	8	23	0,26	Model 1	
Forslund 2015	UC	192	20	172	0,10	Model 1	
Forslund 2015	CD	37	13	24	0,35	Model 1	
Forslund 2015	CTR	372	0	372	0,00	Model 2	
Forslund 2015	T2D	78	0	78	0,00	Model 2	
Forslund 2015	T1D	31	0	31	0,00	Model 2	
Forslund 2015	UC	192	1	191	0,01	Model 2	
Forslund 2015	CD	37	1	36	0,03	Model 2	
Qin 2012	CTR	185	51	134	0,28	Model 1	PRJNA422434
Qin 2012	T2D	183	76	107	0,42	Model 1	
Qin 2012	CTR	185	1	184	0,01	Model 2	
Qin 2012	T2D	183	2	181	0,01	Model 2	
Qin 2014	LD	116	77	39	0,66	Model 1	PRJEB6337
Qin 2014	CTR	114	25	89	0,22	Model 1	
Qin 2014	LD	116	20	96	0,17	Model 2	
Qin 2014	CTR	114	1	113	0,01	Model 2	
Sankaranarayanan 2015	T2D	19	3	16	0,16	Model 1	PRJNA299502
Sankaranarayanan 2015	T2D	19	1	18	0,05	Model 2	
Schirmer 2016	CTR	471	14	457	0,03	Model 1	PRJNA319574
Schirmer 2016	CTR	471	0	471	0,00	Model 2	
Spanish	CP	29	4	25	0,14	Model 1	
Spanish	CP	29	5	24	0,17	Model 2	
Vogtmann 2016	CRC	51	13	38	0,25	Model 1	PRJEB12449
Vogtmann 2016	CTR	52	11	41	0,21	Model 1	
Vogtmann 2016	CRC	51	0	51	0,00	Model 2	
Vogtmann 2016	CTR	52	2	50	0,04	Model 2	
Wirbel 2019	CRC	60	12	48	0,20	Model 1	ERP005534 PRJEB27
Wirbel 2019	CTR	60	4	56	0,07	Model 1	
Wirbel 2019	CRC	60	1	59	0,02	Model 2	
Wirbel 2019	CTR	60	0	60	0,00	Model 2	
Xie 2016	CTR	250	35	215	0,14	Model 1	PRJEB9576
Xie 2016	CTR	250	2	248	0,01	Model 2	

Yachida 2019	CRC	352	88	264	0,25	Model 1	PRJDB4176
Yachida 2019	CTR	289	76	213	0,26	Model 1	
Yachida 2019	CRC	352	28	324	0,08	Model 2	
Yachida 2019	CTR	289	8	281	0,03	Model 2	
Yassour 2018	CTR	42	3	39	0,07	Model 1	PRJNA290381
Yassour 2018	CTR	42	0	42	0,00	Model 2	
Yu 2017	CRC	74	25	49	0,34	Model 1	PRJEB10878
Yu 2017	CTR	54	14	40	0,26	Model 1	
Yu 2017	CRC	74	2	72	0,03	Model 2	
Yu 2017	CTR	54	0	54	0,00	Model 2	
Zeevi 2015	CTR	900	81	819	0,09	Model 1	PRJEB11532
Zeevi 2015	CTR	900	1	899	0,00	Model 2	
Zeller 2014	CTR	83	10	73	0,12	Model 1	PRJEB6070, PRJEB2[illegible]
Zeller 2014	CRC	49	18	31	0,37	Model 1	
Zeller 2014	CTR	83	4	79	0,05	Model 2	
Zeller 2014	CRC	49	5	44	0,10	Model 2	
Zhu 2018	BRCA	62	22	40	0,35	Model 1	PRJNA453965
Zhu 2018	CTR	71	25	46	0,35	Model 1	
Zhu 2018	BRCA	62	3	59	0,05	Model 2	
Zhu 2018	CTR	71	0	71	0,00	Model 2	

Supplementary Table S7

Target species/genus	Sequence	Probe	Dye	Reference
Bifidobacterium (genus)	5'- GATAGGACGCGACCCCAT -3'	Bif228	Cy3	http://probebase.csb.univie.ac.at/pb_report/probe/3959
Veillonella (genus)	5'- AGACGCAATCCCCTCCTT -3'	Veil223	FITC	http://probebase.csb.univie.ac.at/pb_report/probe/553
Akkermansia (genus/species)	5'- CCTTGCGGTTGGCTTCAGAT -3'	MUC-1437	FITC	http://probebase.csb.univie.ac.at/pb_report/probe/3898
Lactobacillus (genus)	5'- ACATGGAGTTCCACT -3'	Lact663	FITC	https://repositorium.sdum.uminho.pt/bitstream/1822/24334/1/pp.pdf
Bacteroides (genus)	5'- CCAATGTGGGGGACCTT -3'	Bac303	Cy3	https://onlinelibrary.wiley.com/doi/pdf/10.1111/j.1365-2672.2011.05039.x
Streptococcus (genus)	5'-TTTAGCCGTCCCTTTCTGG -3'	Strc493	Cy3	http://probebase.csb.univie.ac.at/pb_report/probe/964
