# Supplementary material for: A faecal microbiota signature with high specificity for pancreatic cancer
Source: Gut. 2022 Mar 8;71(7):1359–72. doi: 10.1136/gutjnl-2021-324755 (PMC9185815; doi:10.1136/gutjnl-2021-324755)
Supplement: Supplementary data [file gutjnl-2021-324755supp001.pdf]

SUPPLEMENTARY RESULTS & DISCUSSION

Several PDAC-associated species in the gut may be sourced from the oral cavity.

Many microbial species traverse the gastrointestinal tract to form overlapping populations between the oral cavity and intestine, with increased levels of intra-individual strain transmission associated with diseases such as CRC (83). Indeed, several prominent marker taxa showing fecal enrichment in PDAC are common oral commensals, such as *Veillonella sp.*, *Streptococcus sp.* or *Fusobacterium sp.*. We hypothesized that intestinal populations of these PDAC-associated species were primarily of oral origin, with generally enhanced levels of autologous oral-intestinal strain exchange in PDAC patients. Therefore, we explored microbiome links between body sites at the highest taxonomic resolution attainable with metagenomic data, at the level of strain populations.

We quantified oral-to-gut transmission based on the intra-individual overlap of microbial Single Nucleotide Variants (SNVs) for species prevalent in both mouth and gut metagenomes, as a proxy for oral and intestinal strain populations (see Methods, **Supplementary Fig. 15**). We found that viewed across all subjects and species, PDAC was associated with increased levels of oral-intestinal strain population overlap (Cohen's d = 0.33; ANOVA $p<10^{-3}$ when adjusting for species-level effects and technical, demographic and clinical variables). This observation extended to individual PDAC-associated species, with enhanced levels of autologous transmission in several *Veillonellaceae sp.* (*V. dispar,* d=0.71; *V. atypica*, d=0.6; *V. parvula*, d=0.2; *Megasphaera micronuciformis*, d=2.47) and *Streptococcus sp.* (*S. salivarius*, d=0.51; *S. vestibularis*, d=0.49; *S. parasanguinis*, d=0.36). The situation was more nuanced among *Bifidobacteriaceae sp.*, with enhanced transmission in *B. longum* (d=2.16) and *A. omnicolens* (d=1.24), but less strain overlap in *B. dentium* (d=-0.89). However, due to limits in metagenomic coverage and species prevalence, our dataset size did not provide sufficient statistical power to significantly discern these trends for

individual species with confidence, in particular when adjusting for putative confounders and correcting for multiple tests. Nevertheless, our data indicates that PDAC patients showed overall enhanced levels of oral-intestinal transmission, and that intestinal strain populations of PDAC signature species may be sourced autologously from the oral cavity.

False positive PDAC detections in external validation populations may be due to technical artefacts.

We note that for both model-1 and model-2, at least some false predictions in external validation sets may be attributable to technical artefacts: technical variation between studies often exceeds biological differences in microbiome composition (96), while shallower metagenomic sequencing depths skew taxonomic profiles and bias against lowly abundant species. Moreover, by design, the external validation sets were matched for neither age nor sex, and information on clinical variables with relevance to PDAC was usually not collected or not publicly available. The highest false detection rates were observed among populations with much younger subjects than would normally be considered a PDAC risk group (**Supplementary Fig. 12**). To overcome such limitations, meta-studies of multiple geographically and ethnically diverse PDAC cohorts will be required to further establish globally consistent PDAC microbiome signatures, as has been successfully shown for colorectal cancer (38,97).

Univariate associations of individual species may be informative, but not specific to PDAC.

Species enriched in PDAC included various *Veillonella sp., Alloscardovia omnicolens,* and *Methanobrevibacter smithii*, among others **(Fig. 1c and Fig. 2a**). We confirmed that these were generally not univariately associated with putative confounding factors (**Supplementary Fig. 7**), yet we note that several among them have previously been linked to both health and disease. For example, *Veillonella sp.* are common oral and gut commensals and have been associated with exercise performance in athletes (98), but also with various disease states including cystic fibrosis

(a PDAC risk factor) (99), several infections including meningitis (100), as well as lung (101) and oral carcinomas (102). The role of *Methanobrevibacter smithii,* a prevalent methanogenic archaeon, in the human gut remains poorly understood (103,104), but the species has likewise been associated with athletic performance (105) and disease states (104) such as anorexia nervosa (106,107) and irritable bowel disease (108). This indicates that individual univariate species associations may be informative, but not specific to PDAC. In contrast, our multi-species classifier model-2, capturing a combined signature of PDAC-enriched species, provided very high disease specificity.

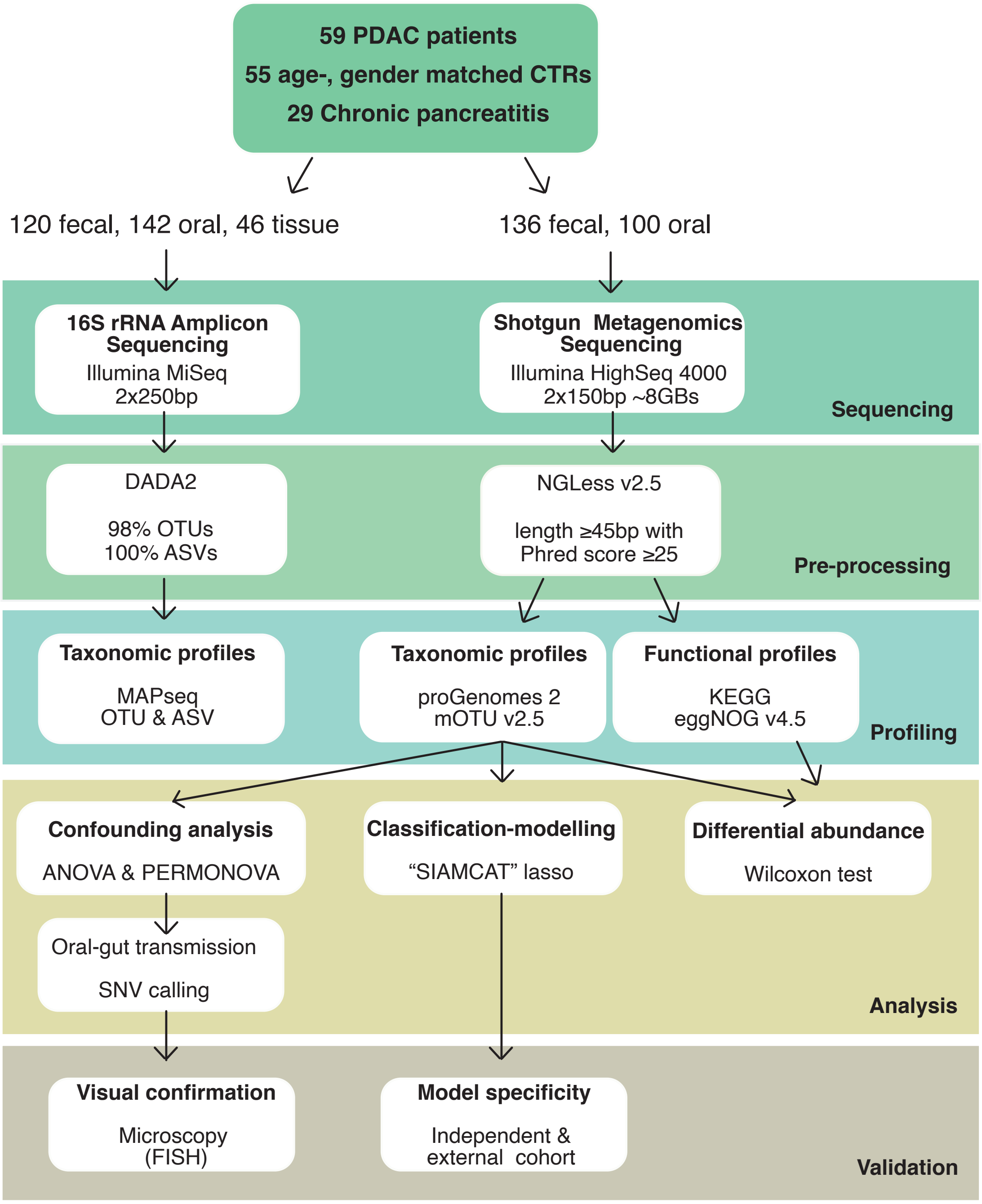

Figure S1. Analysis workflow.
Diagram of analysis steps for 16S rRNA amplicon sequencing data and for shotgun metagenomics sequencing data.

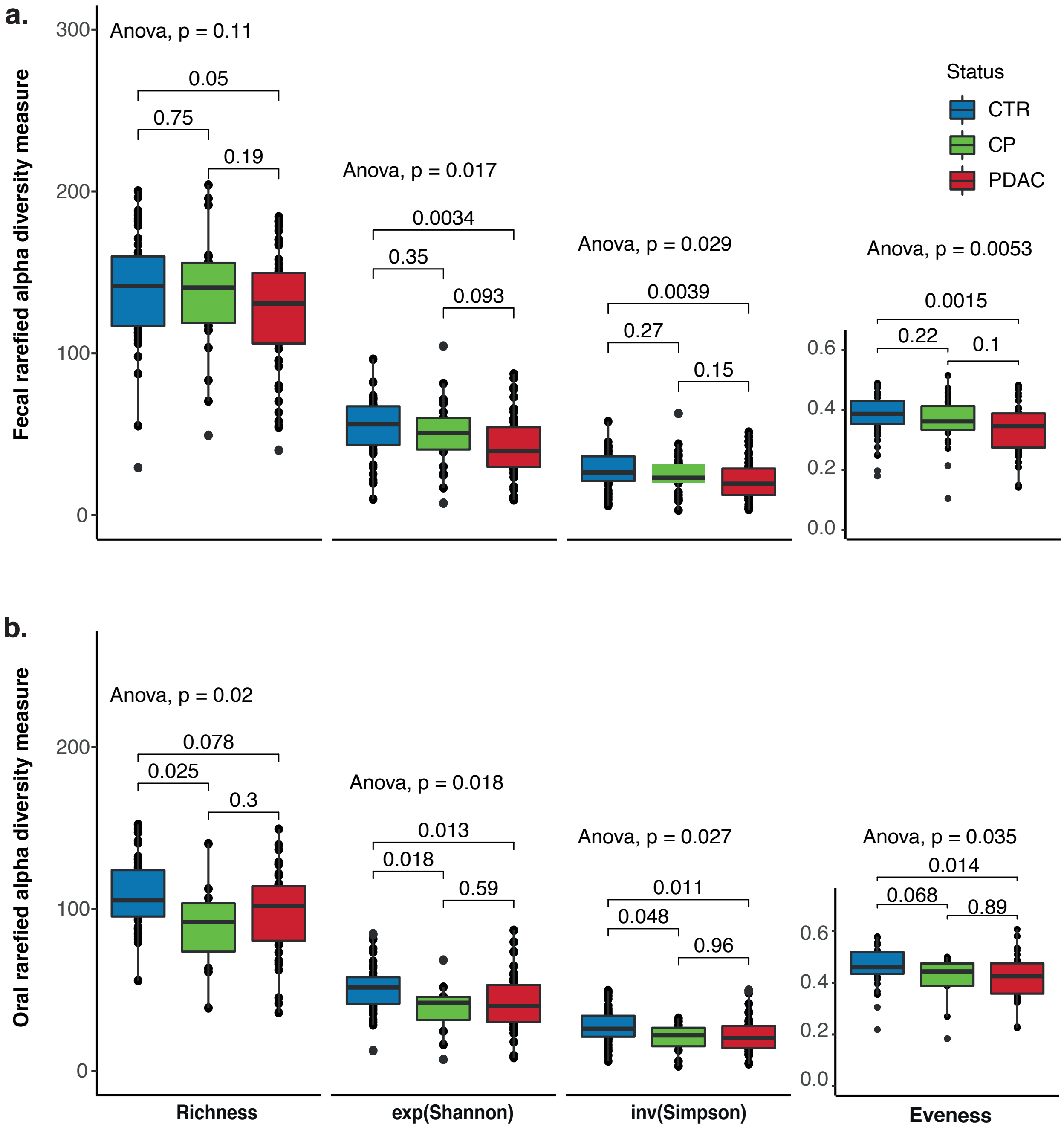

Figure S2. Alpha diversity measurements comparing PDAC and CP patients with controls.
Alpha diversity metrics for (a) fecal and (b) oral samples calculated as richness, exponential Shannon index (exp(Shannon)), inverse Simpson index (inv(Simpson)) and eveness. Colors denote groups, with blue for controls (CTR), green for chronic pancreatitis (CP) patients and red for PDAC cases. Pairwise comparisons were performed using Wilcoxon test and comparisons across all three groups were performed using ANOVA (see Methods).

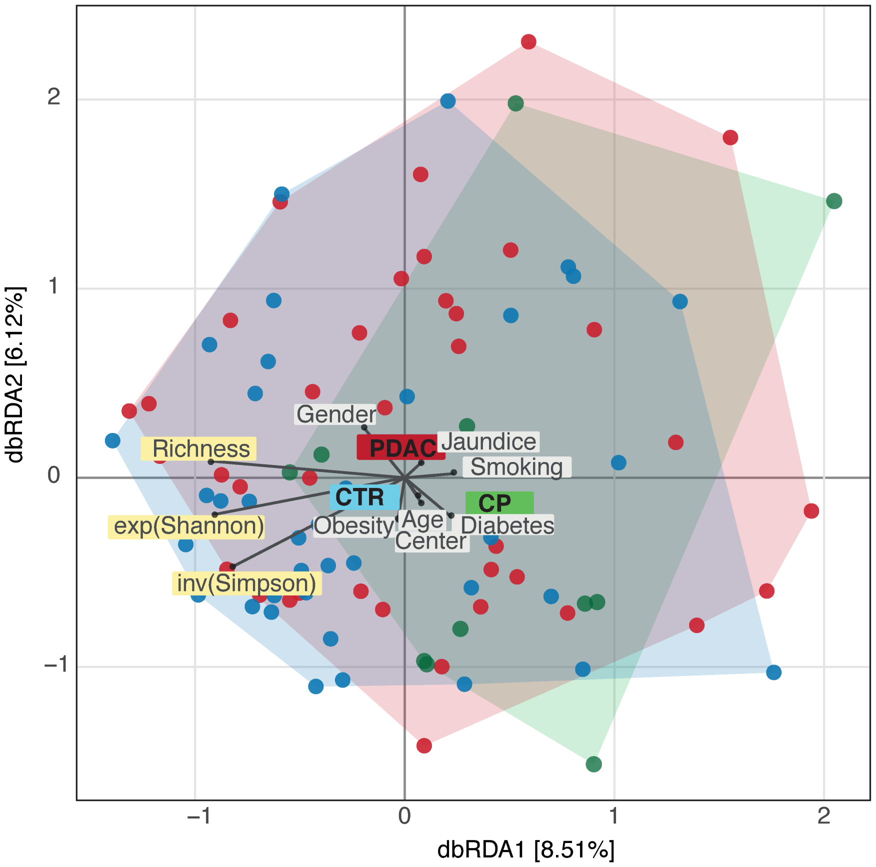

Figure S3. Distance-based redundancy analysis of saliva microbiome.
Bray-Curtis distance-based redundancy analysis (dbRDA) of PDAC, CP and control saliva microbiome data. PDAC samples are shown as red circles, CP patients as green and controls as blue. Association with metadata variables are shown as labeled lines. Richness, exponential Shannon (exp(Shannon)) and inverse Simpson (inv(Simpson)) diversity measures are also visualized with lines and were analysed similarly to metadata variables. The length of the metadata variable line represents the confounding effect size (see Methods).

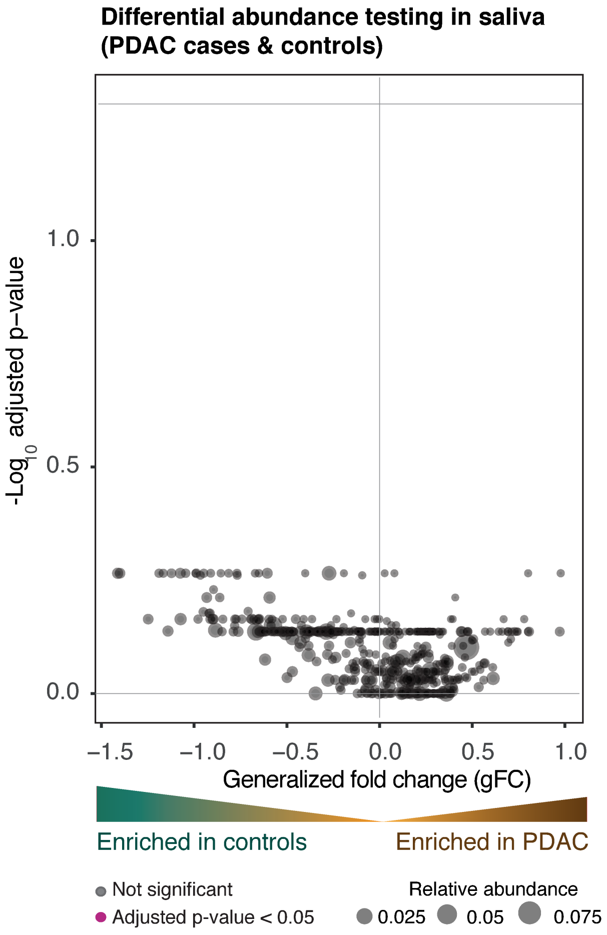

Figure S4. Differential abundance testing of saliva microbiome
Wilcoxon test results of saliva microbiome data to test for enrichment of taxa between PDAC cases and controls (see Methods). Y-axis is log10(FDR corrected p-values), x-axis is generalized fold change and dot size represents the relative abundance of given species and strains. Red dots represent significantly differentially abundant species/strains in either group, while black dots show non-significant species after FDR correction.

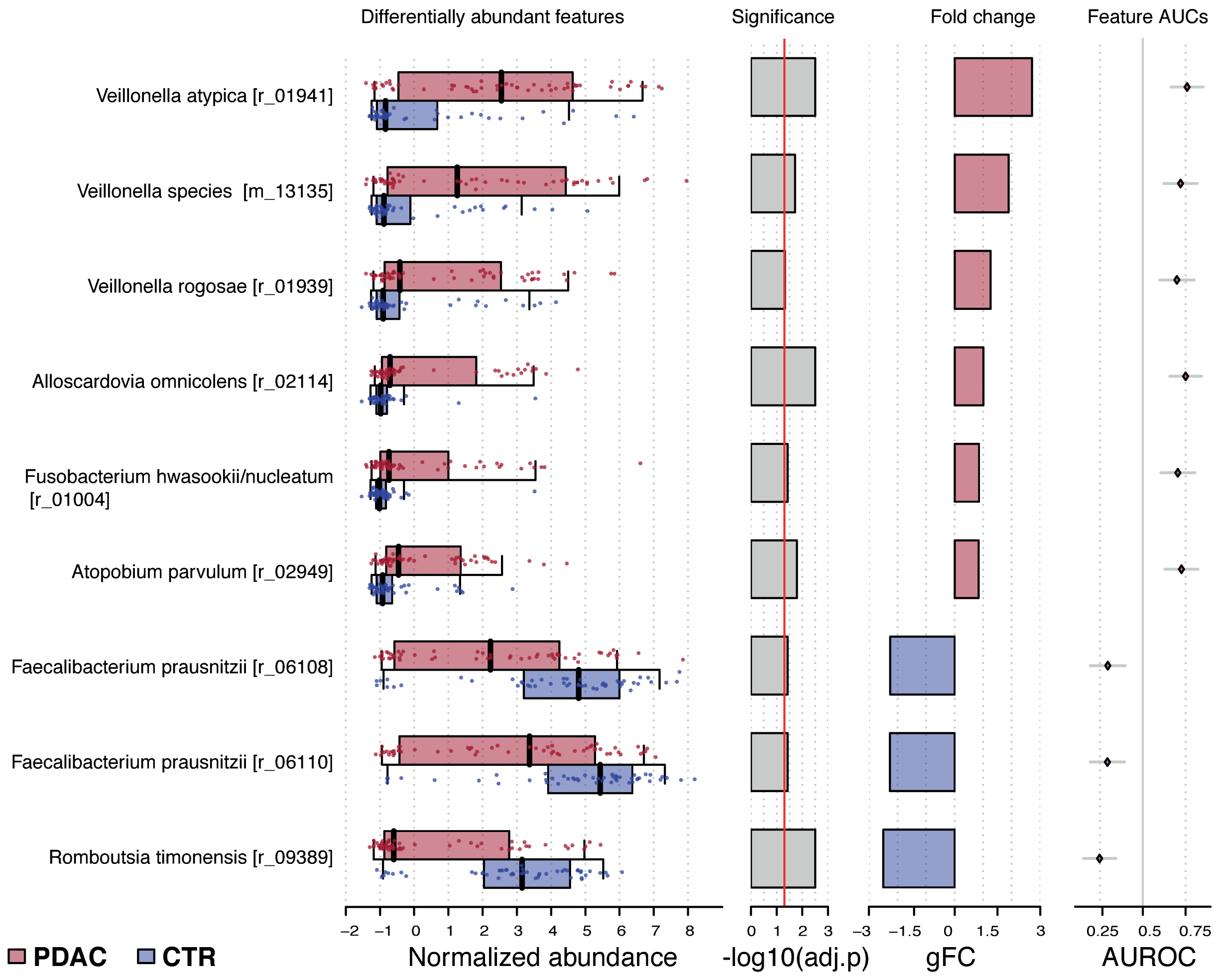

Figure S5. Differentially abundant species in fecal microbiome between PDAC cases and controls.
First column panel shows the differentially abundant species between PDAC cases (red) and controls (blue). Middle panels display the log10(FDR corrected p-values) and generalized fold change for each taxon and the last panel presents the AUC of each feature to distinguish cases from controls. gFC:Generalized fold change.

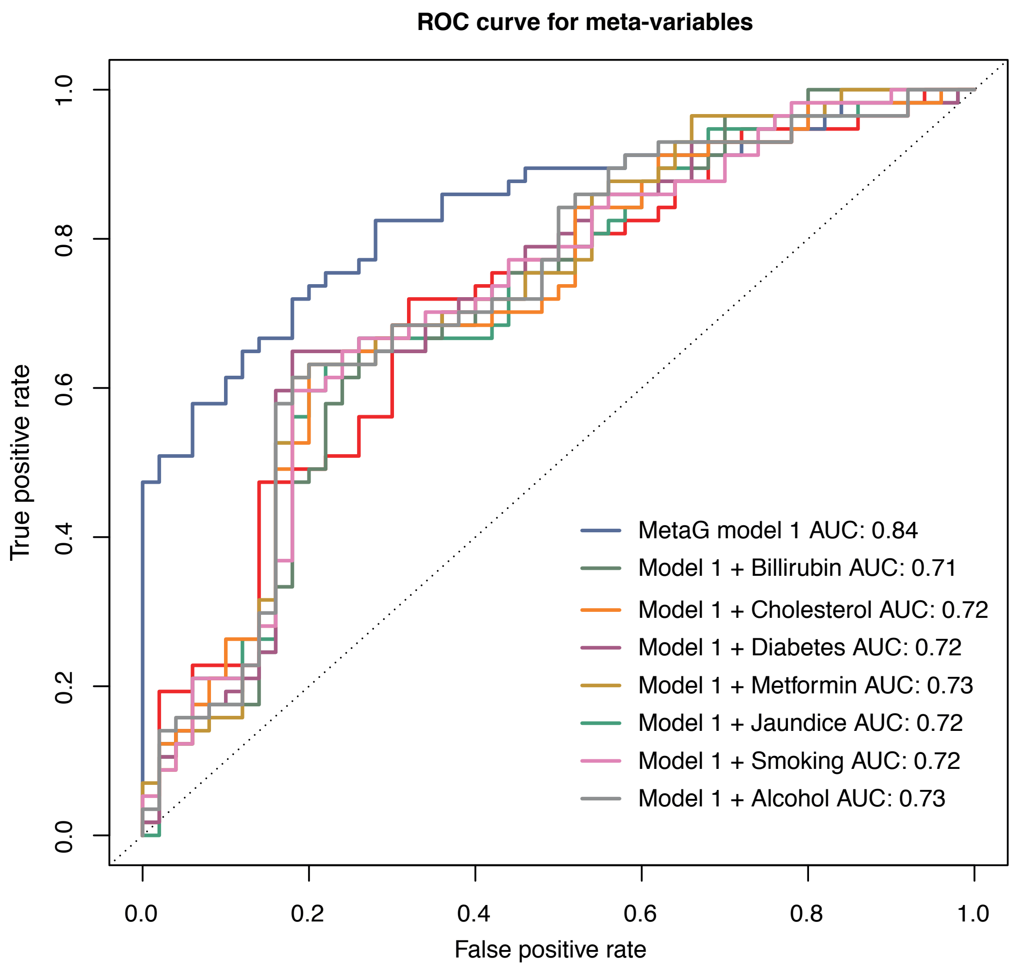

Figure S6. Contribution of confounding factors to the model.

The area under the ROC curve (AUROC) is used to show the performance of lasso_ll model based on fecal microbiome data of PDAC and control samples with 10 times resampling and 10 cross validation (see Methods). Each color corresponds to one specific model based on metagenomics features with an additional metadata variable. Shown metadata variables were added to the metagenomics features table with “add.meta.pred” function from “SIAMCAT” package v1.5.0.

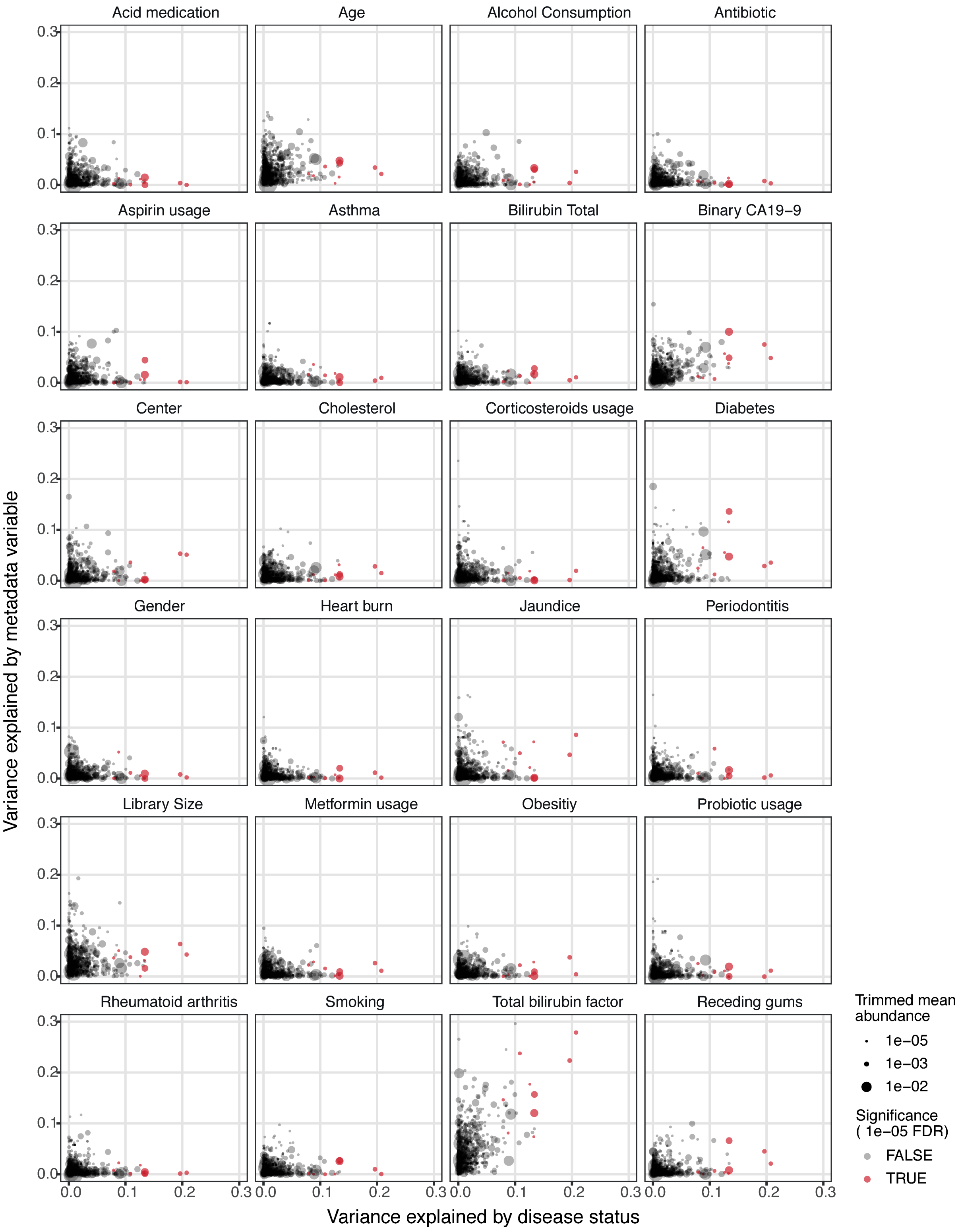

Figure S7. Potential confounder of single species associations by individual demographic and technical variables.

Variance explained by diagnosis is represented against confounding factors for single microbial species. Each circle is a strain or species and is colored red if it is differentially abundant between PDAC cases and controls. The size of each circle represents the mean abundance of that species or strain. Disease status and the tested variables were used as explanatory variables in the linear model for feature abundance.

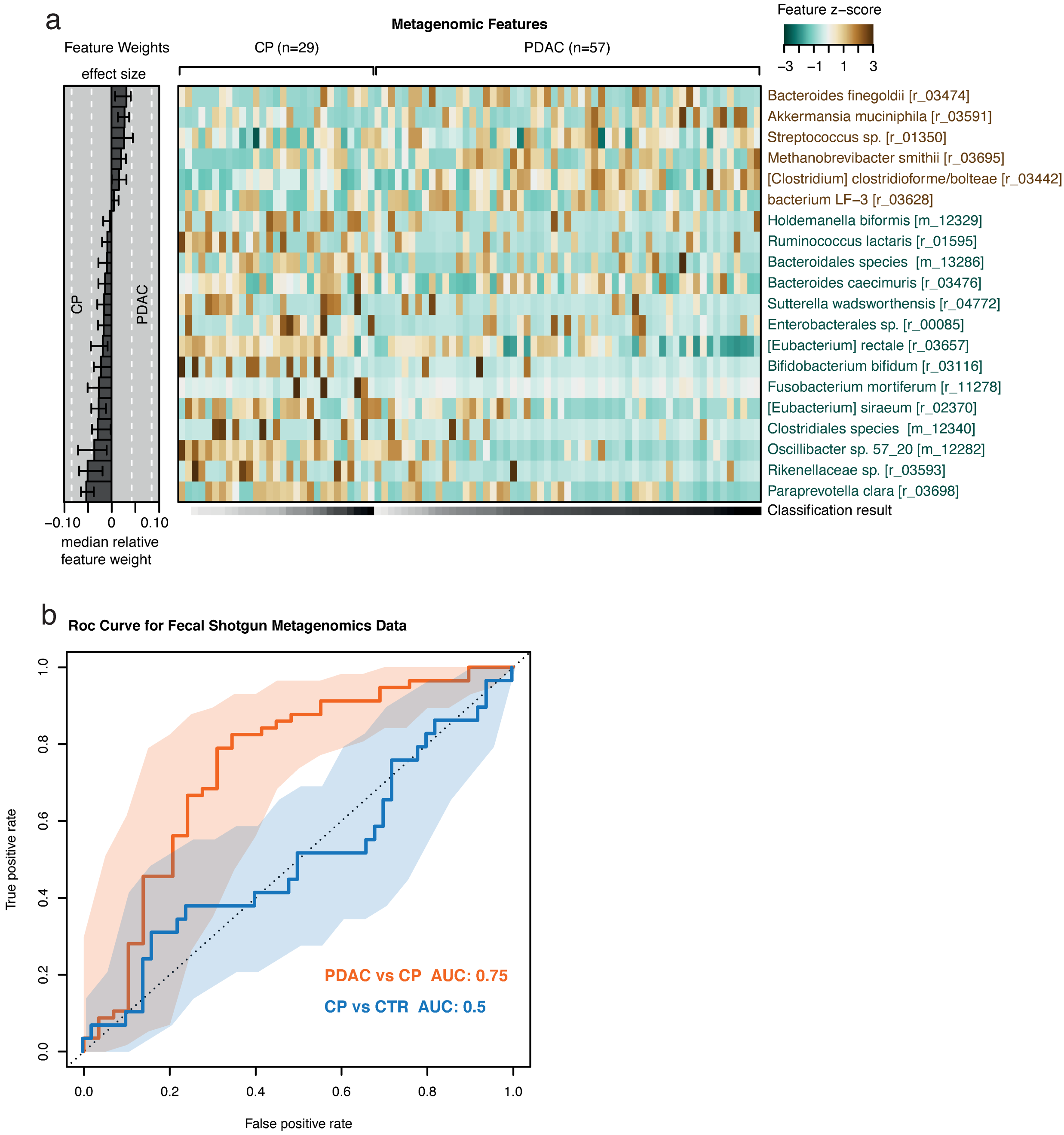

Figure S8. Fecal microbiome-based classifier distinguishes chronic pancreatitis cases from PDAC patients
(a) Heatmap representing the selected metagenomic features in the lasso_ll regression model between PDAC cases and chronic pancreatitis (CP) patients in the fecal microbiome data. (b) ROC curve based on 10 resamplings and 10-fold cross validation (see methods). The blue line represents the model for CP versus controls and the orange line for PDAC vs CP cases. Internal cross validation results are shown as receiver operating characteristic (ROC) curve with a 95% confidence interval shaded in corresponding color.

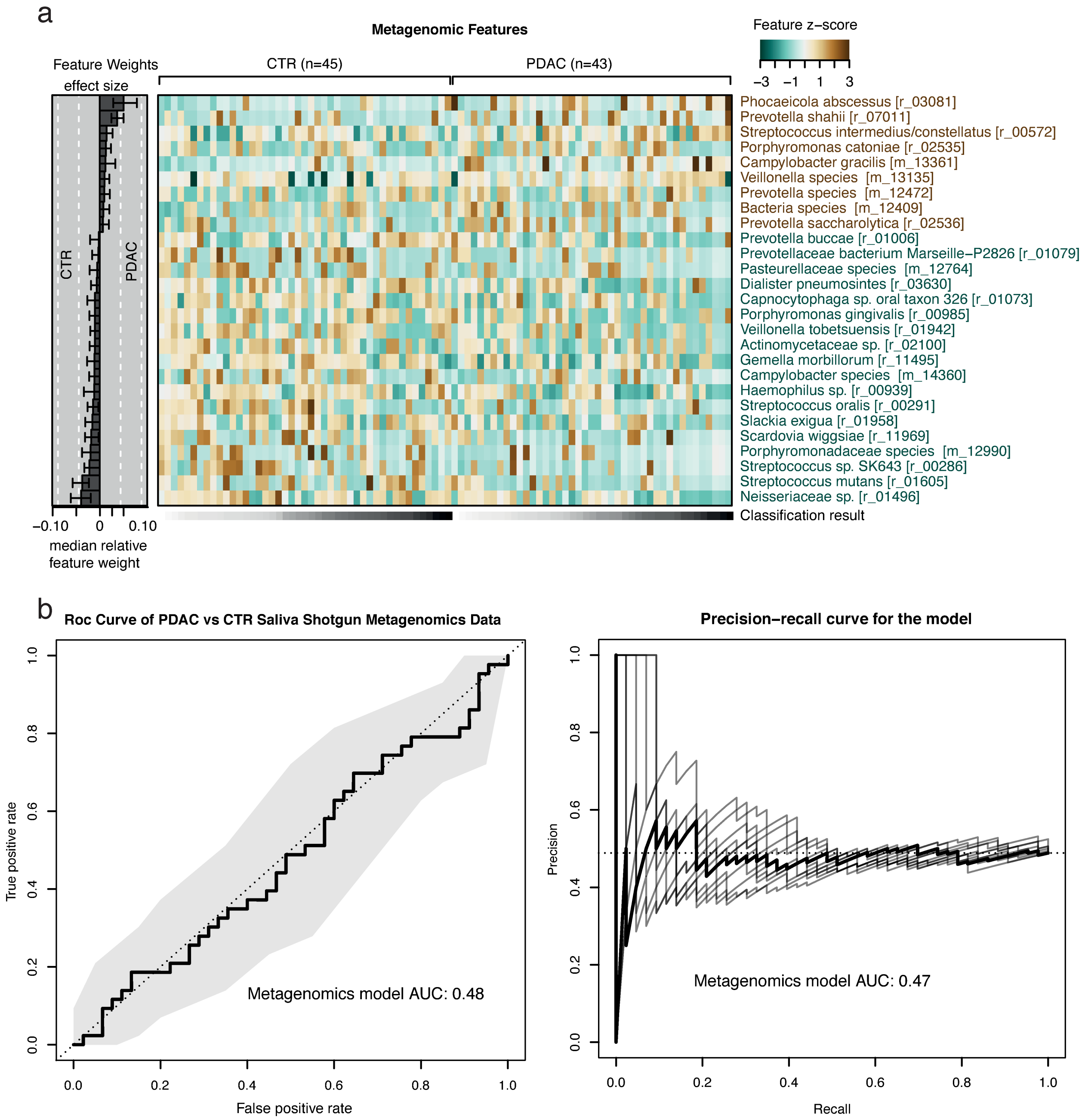

Figure S9. Oral microbiome does not distinguish PDAC samples from control samples.
(a) Heatmap representing the selected metagenomic features in the lasso_ll regression model between cases and controls in the saliva microbiome data. (b) ROC curve based on 10 resamplings and 10-fold cross validation (see methods) and precision recall curve. Internal cross validation results are shown as receiver operating characteristic (ROC) curve with a 95% confidence interval shaded in grey.

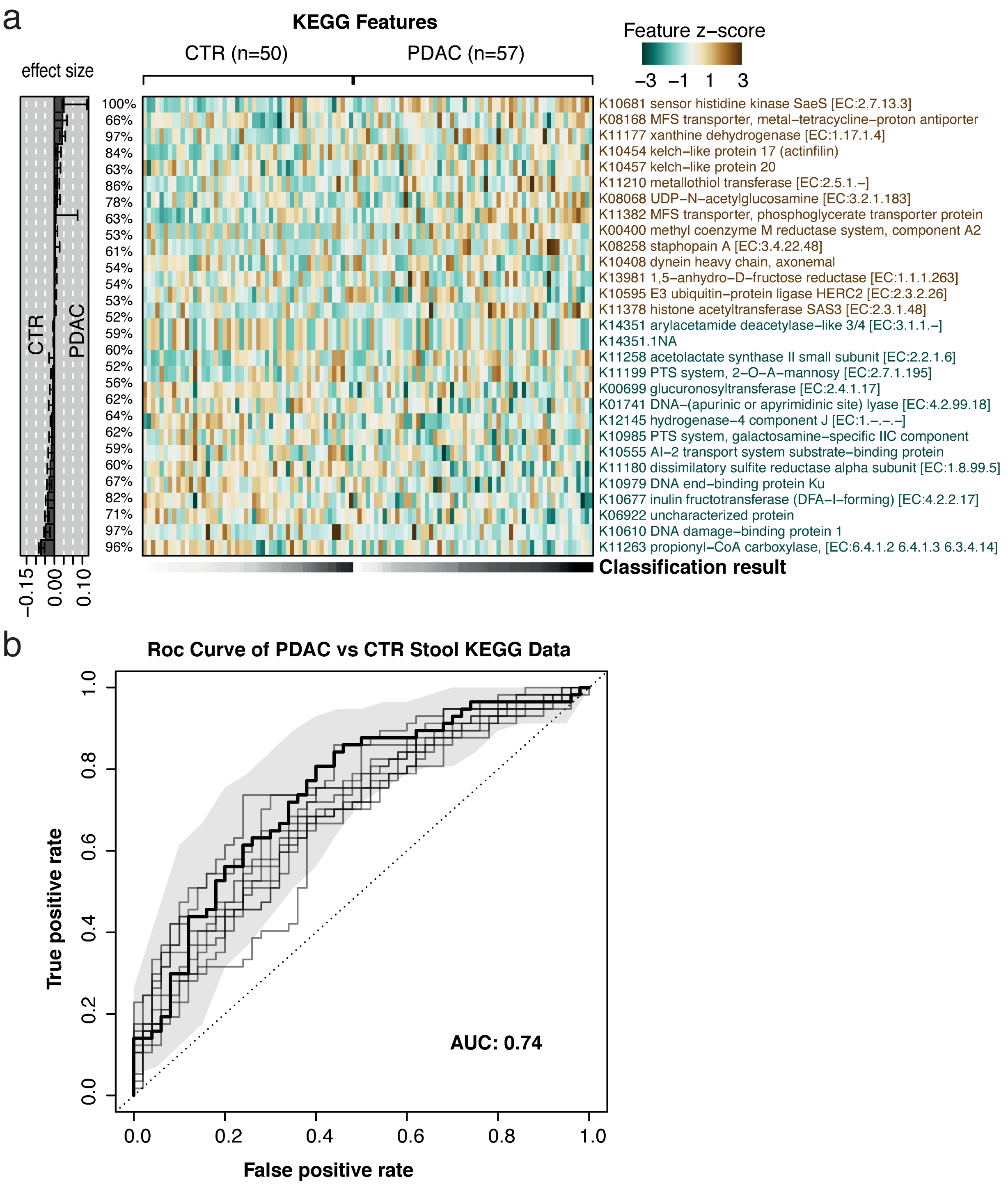

Figure S10. Lasso_ll regression model based on top 200 KEGG modules.
(a) Heatmap representing the selected KEGG modules in the lasso_ll regression model. (b) ROC curve based on 10 resamplings and 10-fold cross validation (see methods).

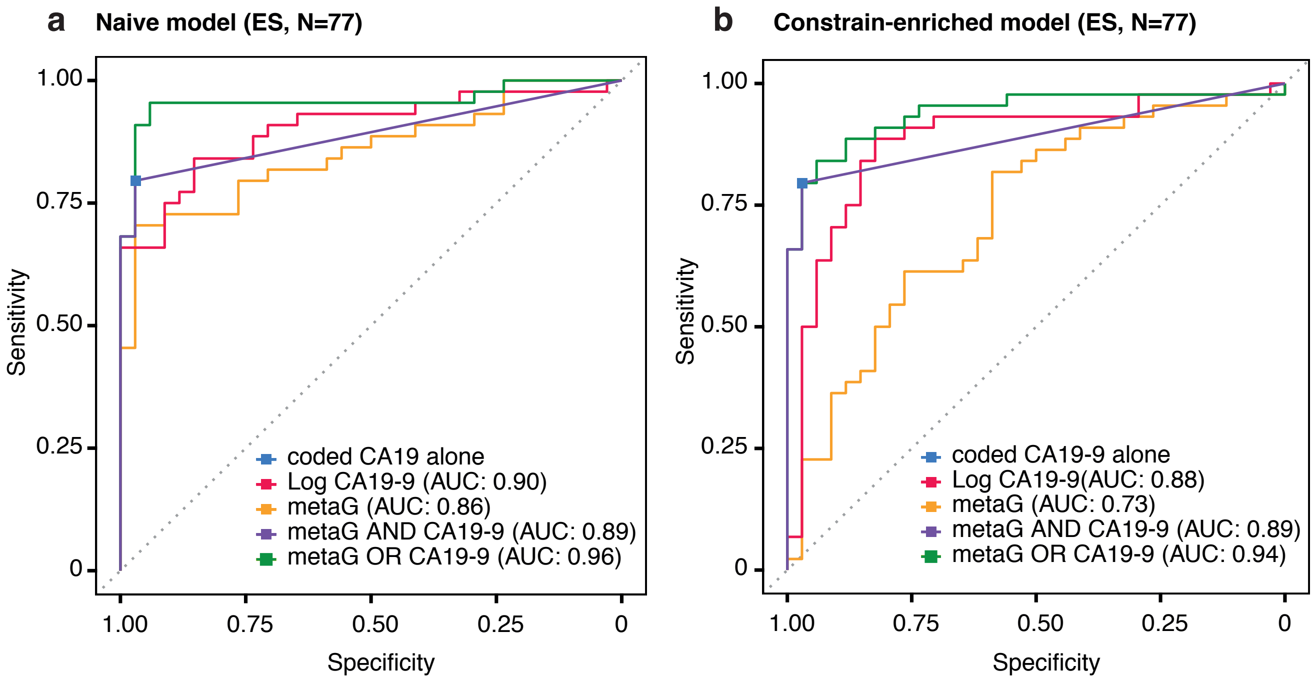

Figure S11. Combination of fecal microbiome data with CA19-9 results increase sensitivity.
77/107 (33/50 CTRs and 44/57 PDAC cases) individuals in Spanish (ES) whom CA19-9 data were available included in the modelling process explicitly. CA19-9 values were converted to binary values (>37ul/ml = 1 & <37ul/ml = 0) **(a)** ROC curve of full feature set. **(b)** ROC curve of enrichment-constrained models based on 77 individual fecal microbiomes. Coded CA19-9 is the binary version of data, which is represented by a blue dot. Log(CA19-9) is displayed with red, while "AND" and "OR" combinations are shown with purple and green respectively. 8/32 CTRs and 43/44 PDAC patients in the German (DE) cohort

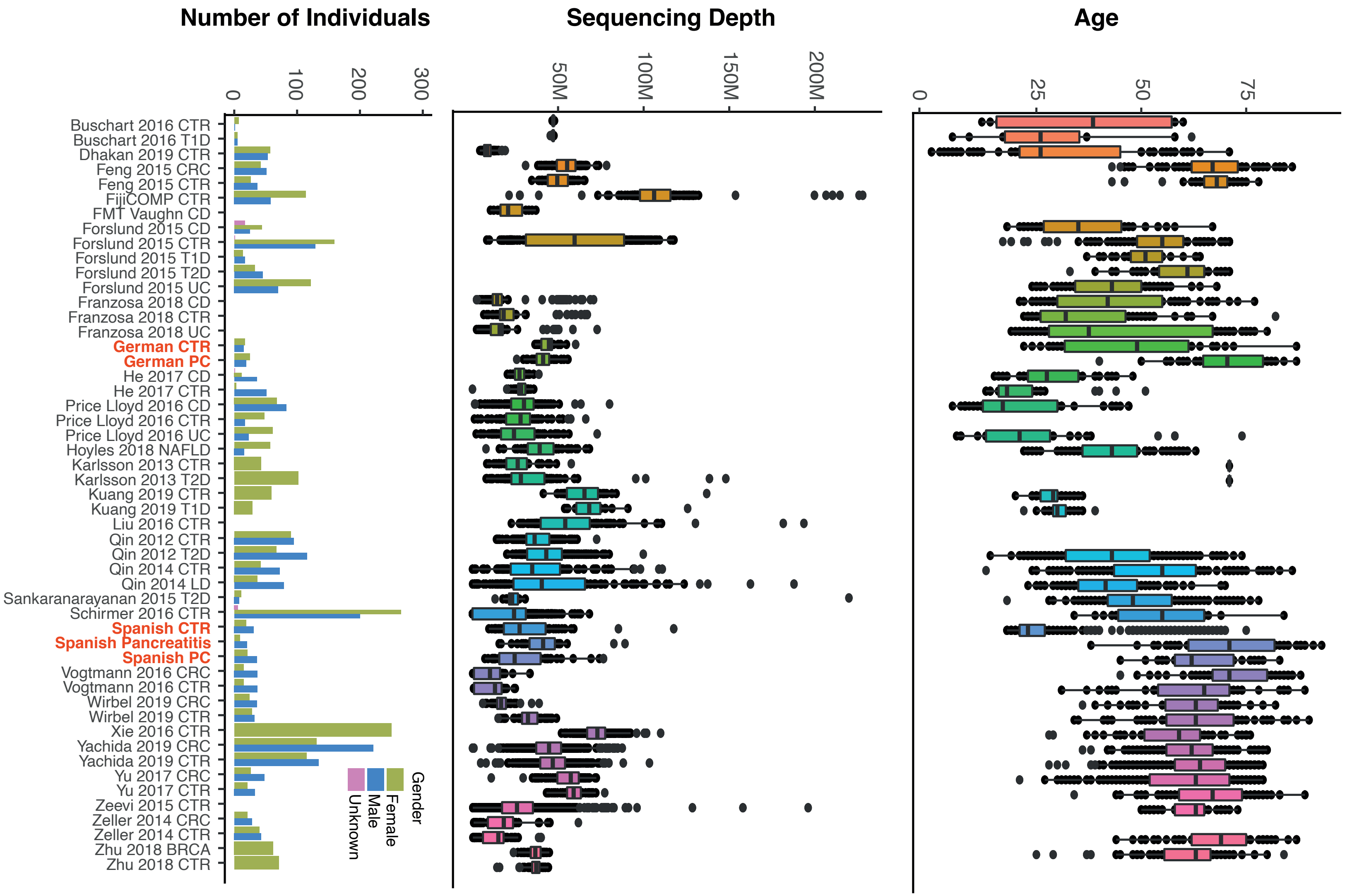

Figure S12. The overview of external validation cohorts. **(a)** Age distribution is shown for all external datasets per group. X-axis shows all the studies and y-axis displays the age distribution. **(b)** Sequencing depth is represented across cohorts. **(c)** Gender information is displayed if available as bar plot. Green is used for females while blue is for males for available studies. M:Million.

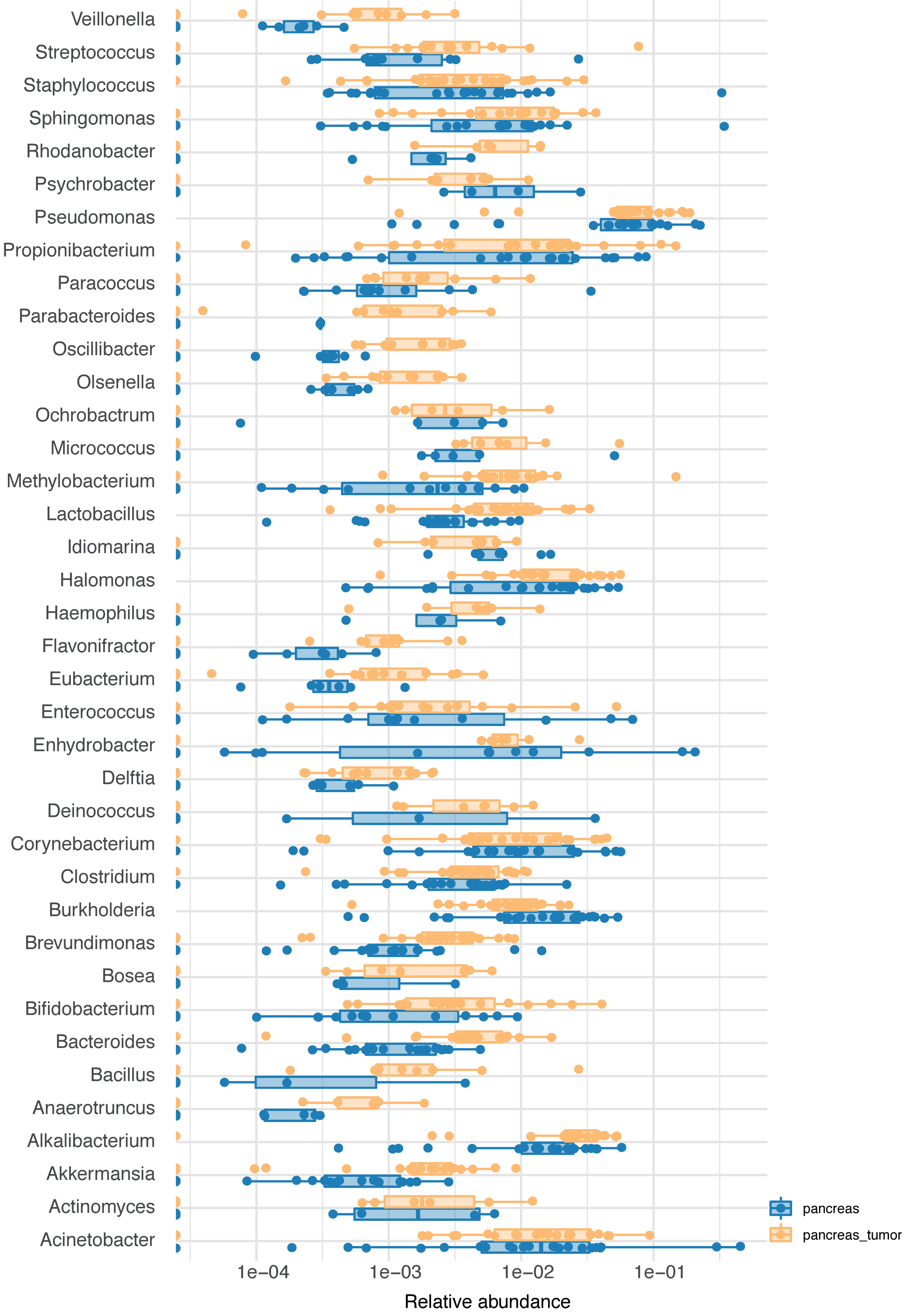

Figure S13. Relative abundance of genera in tumor and non-tumor pancreatic tissue.
Relative abundance of several genera is shown as bar plots. Orange is used to present the pancreatic tumor tissue, while blue is used for non-tumor tissue.

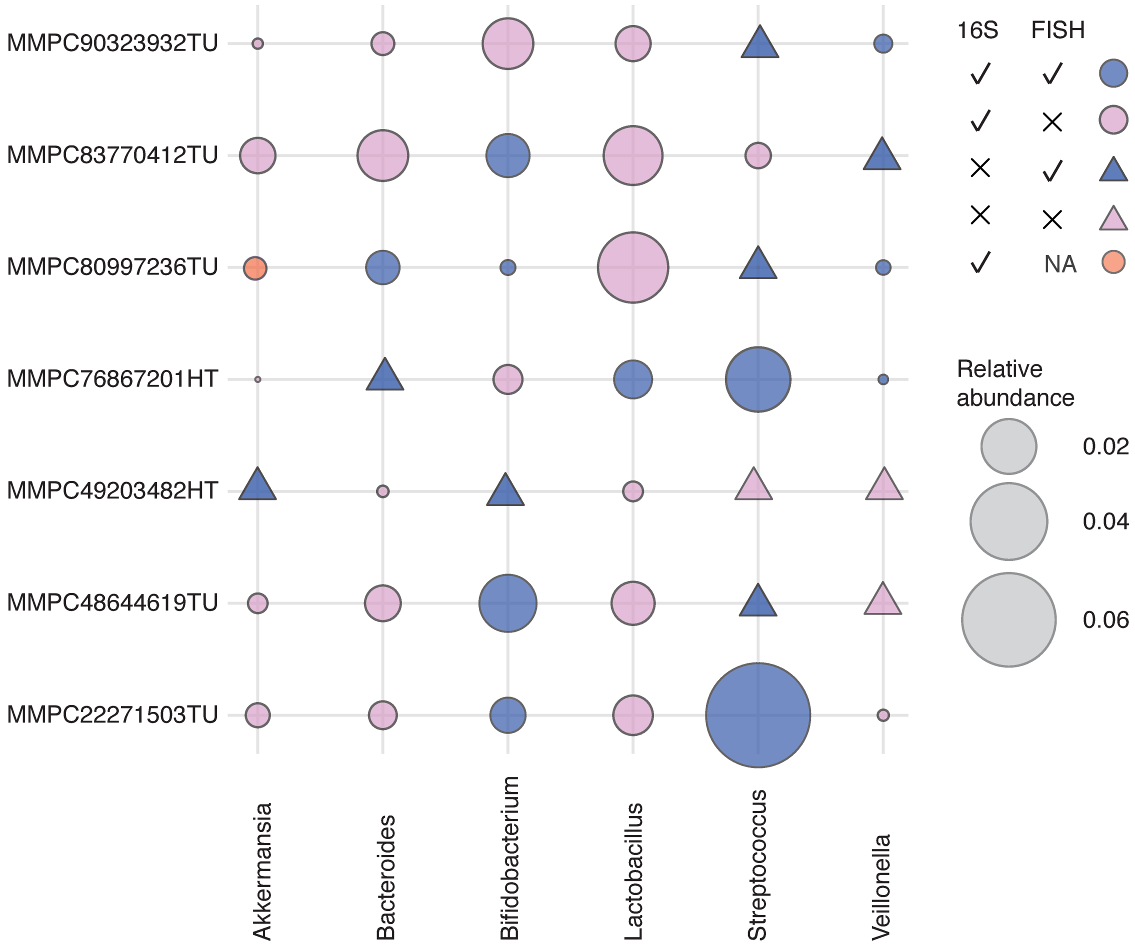

Figure S14. Detailed information of tested samples via in-situ hybridization (FISH).
Rows display the tested samples and columns show the tested genera. The size of the dot represents relative abundance of genus in the given sample. Triangles show that 16S was negative for given samples and color code displays if FISH was positive (blue) or negative (pink). One sample, displayed in orange, did not have enough tissue material for FISH testing.NA: Not available.

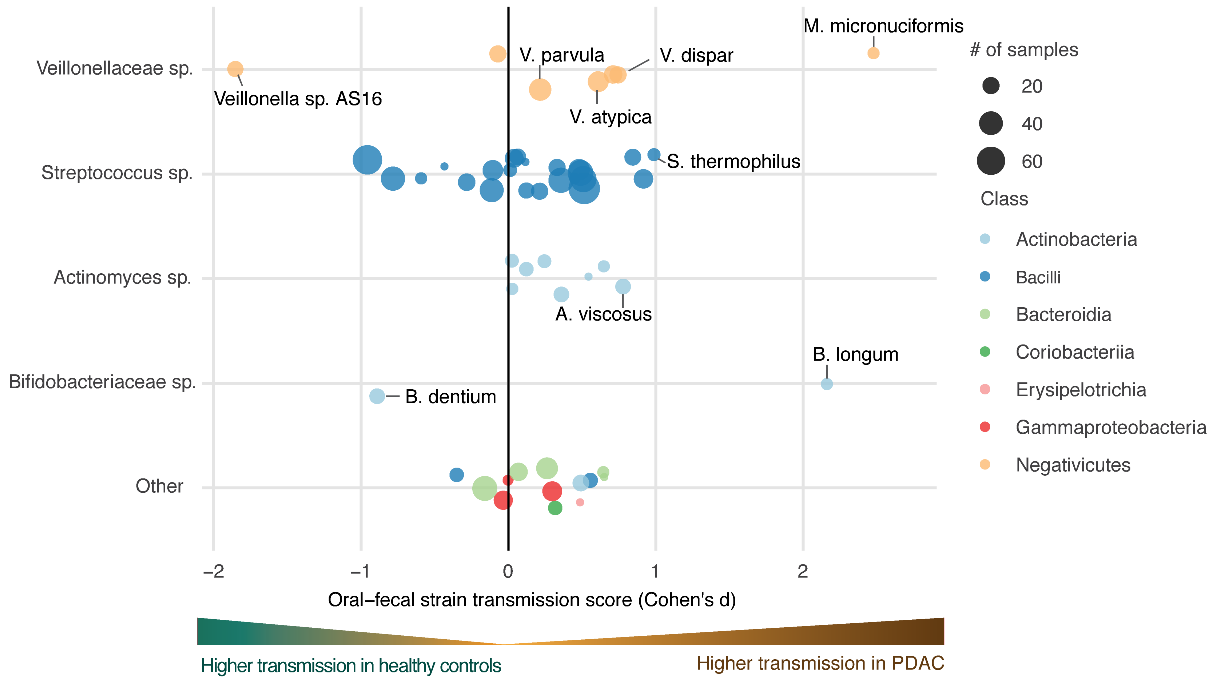

Figure S15. Oral-fecal transmission scores differ between PDAC cases and controls.
Oral-gut transmission scores (y-axis) of each species are displayed grouped by genus (x-axis). The number of subjects is represented by the size of the circle and the color represents the corresponding class group.
